# Exon junction complex components Y14 and Mago still play a role in budding yeast

**DOI:** 10.1038/s41598-018-36785-3

**Published:** 2019-01-29

**Authors:** Anita Boisramé, Hugo Devillers, Djamila Onésime, François Brunel, Juliette Pouch, Mathieu Piot, Cécile Neuvéglise

**Affiliations:** 1 Micalis Institute, INRA, AgroParisTech, Université Paris-Saclay, 78350 Jouy-en-Josas, France; 2 Genomic facility, Institut de Biologie de l’Ecole Normale Supérieure (IBENS), Ecole Normale Supérieure, CNRS, INSERM, PSL Université Paris, 75005 Paris, France

## Abstract

Since their divergence from Pezizomycotina, the mRNA metabolism of budding yeasts have undergone regressive evolution. With the dramatic loss of introns, a number of quality control mechanisms have been simplified or lost during evolution, such as the exon junction complex (EJC). We report the identification of the core EJC components, Mago, Y14, and eIF4A3, in at least seven Saccharomycotina species, including *Yarrowia lipolytica*. Peripheral factors that join EJC, either to mediate its assembly (Ibp160 or Cwc22), or trigger downstream processes, are present in the same species, forming an evolutionary package. Co-immunoprecipitation studies in *Y*. *lipolytica* showed that Mago and Y14 have retained the capacity to form heterodimers, which successively bind to the peripheral factors Upf3, Aly/REF, and Pym. Phenotypes and RNA-Seq analysis of EJC mutants showed evidence of Y14 and Mago involvement in mRNA metabolism. Differences in unspliced mRNA levels suggest that Y14 binding either interferes with pre-mRNA splicing or retains mRNA in the nucleus before their export and translation. These findings indicate that yeast could be a relevant model for understanding EJC function.

## Introduction

The metabolism of messenger RNA (mRNA) is a complex, multi-step process that involves various cellular processes, from the splicing of pre-messenger RNA (pre-mRNA) in the nucleus to their nuclear export, translation in the cytoplasm, and surveillance of cytoplasmic quality-control. A major part of this surveillance is the nonsense-mediated mRNA decay (NMD) pathway that was first described in yeast, nematodes, and then humans^1,2^. NMD ensures the rapid degradation of mRNA molecules that have premature translation termination codons (PTCs), thus preventing the accumulation of non-functional proteins in the cell. As characterization of the NMD process progressed, a question emerged concerning the discrimination between PTCs and normal stop codons by the surveillance complex. The link between nuclear splicing and cytoplasmic NMD was first established in mammals when the presence of a PTC more than 50 nucleotides upstream of an intron was shown to efficiently trigger mRNA degradation^3^. This led to the hypothesis that spliced mRNA carry a “mark” until their translation. Further biochemical studies identified the exon junction complex (EJC), a multiprotein complex that is deposited 20–24 nucleotides upstream of exon-exon junctions as a consequence of pre-mRNA splicing^4,5^.

In mammals, an EJC core is assembled during pre-mRNA splicing. The core is composed of the MAGO, Y14^6,7^ and eIF4A3 proteins^8,9^. The BTZ/CASC3/MLN51 protein was also found to be associated with the mammalian EJC^10^. EJC core formation requires peripheral factors. IBP160, an RNA helicase of the spliceosome may be required to recruit EJC core proteins to introns prior to EJC assembly^11^. More recently, CWC22, a component of the spliceosome complex, was shown to be responsible for deposition of the EJC core through its interaction with eIF4A3^12–14^. eIF4A3 fulfills its function in the EJC after being escorted to the spliced mRNA through its association with CWC22, which aside from promoting EJC assembly, plays an essential role in splicing. eIF4A3 also performs an essential function outside of the EJC. The *S*. *cerevisiae* orthologue, Fal1, is a nucleolar DEAD-box helicase that is required for the maturation of 18S rRNA. The EJC core persists on mRNA after transfer into the cytosol^6,15^ and various nuclear and cytoplasmic proteins are then recruited. The EJC has therefore been defined as a binding platform for factors that are involved in downstream mRNA metabolism, such as mRNA export, translation, and NMD^16^.

Spliced mRNAs leave the nucleus through their interaction with the TAP-p15 export receptor (Mex67-Mtr2 in *S*. *cerevisiae*). Aly/REF has been defined as a peripheral EJC component that connects RNA splicing to their export^17^. Aly/REF acts as an adaptor, which favors the assembly of the export complex on spliced mRNA. Mutations of amino-acid residues involved in Aly/REF binding to the core EJC have been shown to impair the export of intron-containing mRNA^18^.

The EJC also allows increased mRNA translation and at least two other peripheral components have been described as actors in this process^19^. One is MLN51, which has been shown to be involved in the activation of translation in metazoans^20^. The second is the PYM protein for Partner of Y14 and MAGO^21^. PYM is associated through its C-terminus to translating ribosomes. During the first round of translation, its N-terminus interacts with the MAGO-Y14 heterodimer bound to the translated mRNA leading to EJC disassembly and recycling of its core components to the nucleus^22^. More recently, Chuang and co-workers characterized a Y14 mutant, which is defective in translation enhancement and proposed that the mutation prevents the ability of Y14 to interact with PYM, thereby breaking the connection between the EJC and the translation machinery^23^.

UPF3 is the NMD factor that ensures the connection between mRNA nuclear splicing and quality control mechanisms. Indeed, it has been shown to join the EJC in the nucleus and its interaction with Y14 was shown to persist after mRNA export^16,24^. Kunz *et al*. further confirmed that the NMD-active UPF3 forms a complex with Y14, MAGO, eIF4A3 and BTZ^25^. The crystal structure of a MAGO-Y14-eIF4A3-BTZ-UPF3B-RNA complex elucidated the mechanism of recognition between the EJC and UPF3B^26^. UPF3 further recruits the other NMD factors, *i*.*e*. the UPF2 adaptor protein, which in turn bridges the UPF1 helicase to the EJC^27^. Baird *et al*. performed a whole genome RNA interference (RNAi) screen and identified ICE1 as a novel peripheral EJC factor required for EJC-enhanced NMD^28^.

The mRNA metabolism of budding yeasts has undergone regressive evolution, resulting in a considerable loss of introns and patchy distribution of RNAi in Saccharomycotina^29^. Indeed, many of their quality control mechanisms have been simplified or lost during evolution including the EJC in *Saccharomyces cerevisiae*^30^. Both NMD and the EJC are present in a very distant species, the fission yeast *Schizosaccharomyces pombe*, a representative of the Taphrinomycotina subphylum of Ascomycota, but may function independently^31^. To understand the main models of alternative splicing in the yeast *Yarrowia lipolytica*, Mekouar *et al*.^32^ showed that NMD was active in this yeast, but EJC components were not yet described in this early-branching Saccharomycotina species. This finding raises the question of the evolution of EJC in Ascomycota.

Here, we present a phylogenetic analysis of the EJC components and their associated peripheral factors in Ascomycota. After identification of these genes in several Saccharomycotina, we chose to use *Y*. *lipolytica* as a model to investigate the function of the EJC core factors. Indeed, the genomic sequences of some strains of this species are available^33–35^ and numerous genetic tools have been developed for this tractable yeast. Additionally, the NMD pathway has already been studied and NMD mutants are available^32^. We characterized the core complex *in vivo* and several peripheral components of the EJC. The mutant phenotypes and the results of mutant RNA-Seq analysis argue for a functional role of Y14 in the cell, and to a lesser extent, one for Mago.

## Results

### Some Saccharomycotina yeasts have retained genes of the EJC core

**Figure 1:**
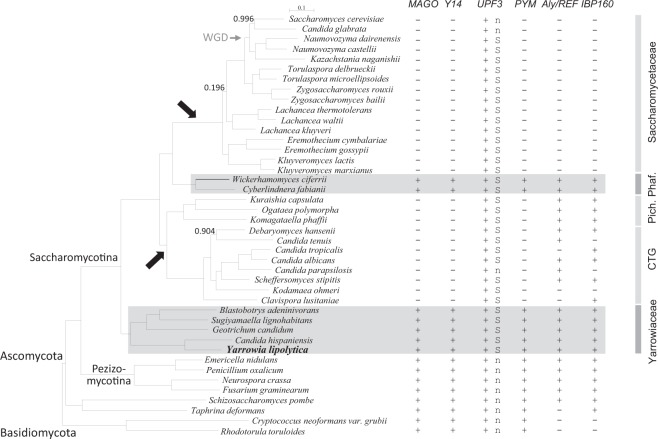
Multiple evolutionary losses of EJC components in yeast. Phylogenetic relationships of 42 yeast and fungi of the Ascomycota and Basidiomycota phyla. The tree is based on the concatenation of 90 groups of proteins (alignment of 32,405 residues) and was constructed by maximum likelihood with PhyML. Branch support was estimated using SH-like approximate likelihood-based method. Only values different from 1 were indicated next to the branches. Taxonomic families are indicated on the right: Pich. for Pichiaceae and Phaf. for Phaffomycetaceae. CTG refers to the clade of species with a genetic code alteration that reassigns CUG codons to serine instead of leucine. Large arrows represent loss of the EJC components *MAGO* and *Y14*, inferred by parsimony. Species in which a homologue of *MAGO*, *Y14*, *UPF3*, *PYM*, *Aly/REF* or *IBP160* has been detected are indicated as positive (+), whereas no significant homology is indicated as negative (−). “S” refers to the conservation of synteny between homologues of *UPF3* (YALI0A10142g) and the nuclear pore-associated protein *THP1* (YALI0A10164g). “n” denotes the absence of synteny conservation. WGD: whole genome duplication.

In metazoans, EJC is essentially a trimeric protein complex with MAGO, Y14 and eIF4A3. A fourth protein, MLN51, joins the complex after the pre-mRNA splicing step^36^. We therefore performed a homology search with the human proteins to find orthologues of these factors in 40 Ascomycota and 2 Basidiomycota genomes. The blast search of the EJC components was performed against annotated proteomes or yeast chromosomal sequences (Supplementary Tables S1 and S2). The results showed that *MAGO* and *Y14* have been widely lost from different lineages, notably Saccharomycetaceae including *S*. *cerevisiae*, and from the CTG clade, including the human pathogen *Candida albicans* (Fig. 1). However, both genes have been retained in early-branching species, including Yarrowiaceae (*Candida hispaniensis*, *Geotrichum candidum*, *Blastobotrys adeninivorans*, *Sugiyamaella lignohabitans* and *Yarrowia lipolytica*) and, unexpectedly, in Phaffomycetaceae (*Cyberlindnera fabianii* and *Wickerhamomyces ciferrii*). This finding shows that losses occurred independently in several lineages, at least two times, *i*.*e*. in the ancestor of Saccharomycetaceae, and before the divergence between CTG and Pichiaceae (Fig. 1).

We then investigated the conservation of the gene sequences in two steps. First, we compared model eukaryotes to *Y*. *lipolytica*, chosen as a Saccharomycotina model. Then, we estimated the level of amino acid conservation within the Saccharomycotina.

*Y*. *lipolytica* Mago and Y14 proteins were aligned with those of *Homo sapiens*, *Drosophila melanogaster*, *Caenorhabditis elegans*, and *Schizosaccharomyces pombe*. The Mago alignment showed that, except for a 45 amino-acid long N-terminal extension, the *Y*. *lipolytica* protein is well conserved, with 62.7% identity with the human protein over 150 amino-acid residues (Supplementary Fig. S1, Table 1). *Y*. *lipolytica* Y14 showed up to 43% identity over 89 amino acids. YlY14 lacks the 50 amino-acid N-terminal extension present in the human and metazoan orthologues, like that of *S*. *pombe* Y14 (Supplementary Fig. S2). Alignment of the *Y*. *lipolytica* Mago and Y14 proteins with the six proteins identified in Yarrowiaceae and Phaffomycetaceae revealed a high level of conservation between the proteins of these two yeast families (80% similarity between *Y*. *lipolytica* and *W*. *ciferrii* Mago and 65% similarity between *Y*. *lipolytica* and *C*. *fabianii* Y14; Supplementary Figs S1 and S2, and Table S3). This suggests that they derive from a common ancestor.

**Table 1:** Homologues of human EJC components and peripheral factors in *Y*. *lipolytica*.

Human (aa)	*Yarrowia lipolytica* (aa)	Identity*	Function
**EJC recruitment factors**			
IBP160 (1485)	YALI0C24079 (1220)	453/1310 (34.6%)	Intron binding
CWC22 (908)	YALI0D20790 (954)	250/547 (45.7%)	eIF4A3 spliceosome recruitment
**EJC core factors**			
eIF4A3 (411)	YALI0F02695/Fal1(397)	298/387 (77.0%)	EJC core component
Y14 (174)	YALI0E02530 (120)	38/89 (42.7%)	EJC core component
Mago (146)	YALI0D26664 (194)	94/150	EJC core component
MLN51/CASC3/BTZ (703)	/	/	EJC core component
**EJC peripheral factors**			
ICE1 (2266)	/	/	Upf3B EJC association
Upf3B (470)	YALI0A10142 (516)	33/143 (23.1%)	NMD factor
Aly-REF (257)	YALI0F07909 (222)	51/155 (31.9%)	mRNA export
PYM (203)	YALI0B01562 (244)	42/203 (20.7%)	EJC disassembly

*Identity between Human and *Yarrowia lipolytica* proteins.

We found eIF4A3, the third component of the EJC core, to be present in all the fungal species we studied, irrespective of the presence of the other EJC components. Moreover, the protein sequence is highly conserved, even with that of model eukaryotes (77% identity between *Y*. *lipolytica* and human homologues (Table 1, Supplementary Fig. S3).

In contrast, MLN51 orthologues were not identified in any of the yeast genomes whereas it is present in human, *Drosophila* and *C*. *elegans*^30^.

The function of the EJC requires its interaction with peripheral components involved in its recruitment to the mRNA (Cwc22, Ibp160) or the downstream steps, *i*.*e*. export from the nucleus (Aly/REF), translation (Pym), and the NMD pathway (Upf3, Ice1). We thus searched for the presence of these proteins in the fungal species under study.

### Peripheral factors are part of an evolutionary package with EJC components

We found a Cwc22 orthologue in each of the 42 species, which may be linked to its functional role in pre-mRNA splicing. The amino-acid residues of Cwc22 involved in its binding to eIF4A3^26^ are present in both the *Y*. *lipolytica* and *S*. *pombe* proteins, but are not conserved in the *S*. *cerevisiae* protein (Supplementary Fig. S4). This suggests that the two proteins no longer interact in *S*. *cerevisiae*, consistent with the absence of both Mago and Y14. More generally, these amino acids are conserved in all species containing EJC components, including *D*. *hansenii*. In contrast to Cwc22, Ibp160 showed patchy distribution in the phylogenetic tree. The gene is absent from Saccharomycetaceae, has been found in most of the CTG species, and is present in all other species (Fig. 1).

ICE1, a peripheral factor that facilitates association between Upf3B and EJC^28^ was not found in any genomes of Saccharomycotina or Basidiomycota.

Upf3 ensures the connection between the EJC and quality control mechanisms, such as the NMD pathway. We found *UPF3* in almost all the studied fungal species. Homology searches for a *Y*. *lipolytica* homologue using known *UPF3* nucleotide sequences or amino acids conserved between yeast, humans, drosophila, and plants were all unsuccessful. We addressed this surprising situation using a recurrent blast approach (see Material and methods) and identified YALI0A10142g as a putative *UPF3*. We then investigated the localization of YALI0A10142g in the genomic sequence and found YALI0A10142g and its flanking gene YALI0A10164g (the nuclear pore-associated protein *THP1*) to be conserved in synteny all along the Saccharomycotina tree, with few exceptions, including *S*. *cerevisiae*, *C*. *glabrata*, and *C*. *parapsilosis* (Fig. 1). We thus considered YALI0A10142g to be the putative *Y*. *lipolytica* Upf3. The amino-acid sequence of Upf3 is poorly conserved in yeasts and more generally in eukaryotes, even in the Smg4_UPF3 pfam domain, as shown in Supplementary Fig. S5. Furthermore, the stretch of 15 residues in the C-terminus of Upf3B, encompassing residues 418 to 432 in humans, which was shown to interact with a composite surface on the EJC^26^, is absent in *C*. *elegans* and *S*. *pombe*, for which the proteins are shorter. However, this domain is partially conserved in *Y*. *lipolytica* Upf3 (Supplementary Fig. S5). Reciprocally, amino acids involved in the interaction with Upf3B in HsMAGO (Asp66 and Glu68) and HsY14 (Tyr112) are conserved in both *Y*. *lipolytica* homologous proteins, namely Glu114 and Glu116 in YlMago and Tyr56 in YlY14 (Supplementary Figs S1 and S2).

A blast search of Pym in Saccharomycotina showed the presence of the protein in Yarrowiaceae and Phaffomycetaceae only, *i*.*e*. in the species possessing genes of the EJC (Fig. 1). Pym sequences from model organisms or yeasts are conserved exclusively in the Mago-binding domain (MBD), which extends from amino acids 12 to 38 in *Y*. *lipolytica* (Supplementary Fig. S6).

Aly/REF acts as an adaptor factor that facilitates assembly of the export complex on spliced mRNA. We identified this peripheral EJC component in a set of genomes larger than the seven EJC-containing Saccharomycotina species. Indeed, it is present in all species, except the Saccharomycetaceae and several CTG species (Fig. 1; Supplementary Fig. S7). Three domains are particularly conserved, a central 70-aa RNA binding domain and 10-aa N-terminal and 10-aa C-terminal domains. The function of the latter domains is unknown, but at least one may be involved in TAP-p15 binding.

The presence of EJC core factors, together with Aly/REF and Pym, in some yeast genomes supports a biological role for these factors. In *Y*. *lipolytica*, the amino acids involved in protein interactions are conserved (Supplementary Figs S1–S7). We thus experimentally investigated the putative interactions between these EJC core and peripheral components in *Y*. *lipolytica*.

### Mago and Y14 form a complex in *Y*. *lipolytica*

**Figure 2:**
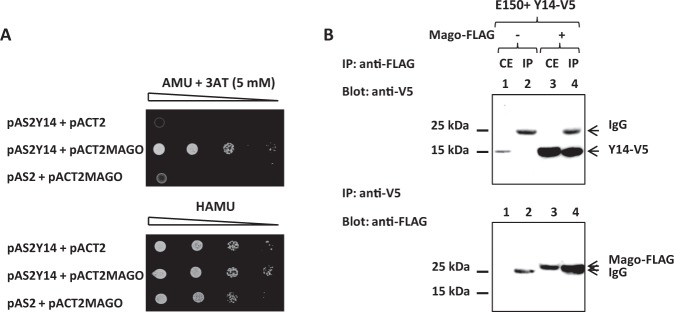
*Yarrowia lipolytica* Mago and Y14 proteins interact. (**A**) Two-hybrid interaction of Mago and Y14. Growth of serial dilutions of PJ69-4A strains were co-transformed with the plasmid combinations pAS2ΔΔ-Y14 + pACT2, pAS2ΔΔ-Y14 + pACT2-MAGO, or pAS2ΔΔ + pACT2-MAGO and tested on minimal medium lacking leucine and tryptophan (lower panel) and minimal medium lacking leucine, tryptophan and histidine + 5 mM 3-aminotriazole (upper panel). (**B**) Co-immunoprecipitation of Y14 and Mago in *Y*. *lipolytica*. Upper panel: anti-FLAG immunoprecipitation was performed on solubilized extracts from membrane-enriched fractions of the simple E150 + p*TEF1*-Y14-V5-FLAG-tagged (lanes 1 and 2) or double E150 + p*TEF1*-Y14-V5 + p*TEF1*-Mago-FLAG-tagged strains (lanes 3 and 4). Immunoprecipitates were eluted at 75 °C in sample buffer and resolved by SDS-PAGE (IP, lanes 2 and 4) along with crude extracts (CE, lanes 1 and 3). Proteins were then blotted with anti-V5. Lower panel, reciprocal experiment: Y14-V5 from the same samples was immunoprecipitated using anti-V5 antibodies and IP were resolved by SDS-PAGE, and blotted with anti-FLAG (lanes 2 and 4) along with CE (lanes 1 and 3). The numbers on the left indicate the molecular masses of the protein standards (Precision Plus ProteinTM Standards from BIORAD) revealed by EZblue staining (Sigma-Aldrich). The revealed proteins are mentioned on the right depending on the antibodies used.

We studied the association of the EJC core components in *Y*. *lipolytica*, by focusing on Mago and Y14 and determining whether they can form a heterodimer, as shown in other eukaryotes^37^. We first used the *S*. *cerevisiae* two-hybrid system. The *Y14* open-reading frame was expressed as a fusion protein with the Gal4p-binding domain in the pAS2ΔΔ plasmid and the *MAGO* open-reading frame was cloned in frame with the Gal4p-activating domain in the pACT2 plasmid. The two control strains that expressed only one of the two EJC proteins did not grow without histidine, but co-expression of both the Gal4BD-Y14 and Gal4AD-Mago fusion proteins allowed the yeast to grow on selective medium devoid of histidine (Fig. 2A, upper panel). Thus, *Y*. *lipolytica* Y14 and Mago can interact directly.

We tested the interaction of the two proteins in *Y*. *lipolytica* by performing a co-immunoprecitation assay with Mago and Y14. A FLAG tag was fused to the C-terminus of Mago and the V5 epitope to the N-terminus of Y14 (Supplementary Table S4). An anti-FLAG immunoprecipitation was performed on solubilized fractions derived from membrane-enriched pellets of both the Mago-FLAG/Y14-V5 double-tagged strain and the Y14-V5 control strain with no Mago-FLAG (Fig. 2B, upper panel). We detected Y14-V5 in the anti-FLAG precipitate from the double epitope-tagged strain only (compare lanes 4 and 2). This shows that Y14 forms a complex with Mago in *Y*. *lipolytica*. The reciprocal experiment confirmed that the two proteins associate. Indeed, Mago-FLAG was specifically detected in the anti-V5 precipitate from the Mago-FLAG/Y14-V5 double-tagged strain (Fig. 2B, lower panel: compare lanes 4 and 2).

The Y14-V5 signal detected for the control strain, which only over-expressed Y14, was weak relative to the strong signal observed for the double epitope-tagged strain (Fig. 2B upper panel, compare lanes 1 and 3). This suggests that Y14 is stabilized by the formation of a heterodimer with its Mago partner.

### The peripheral component Upf3 associates with the *Y*. *lipolytica* Y14-Mago complex

**Figure 3:**
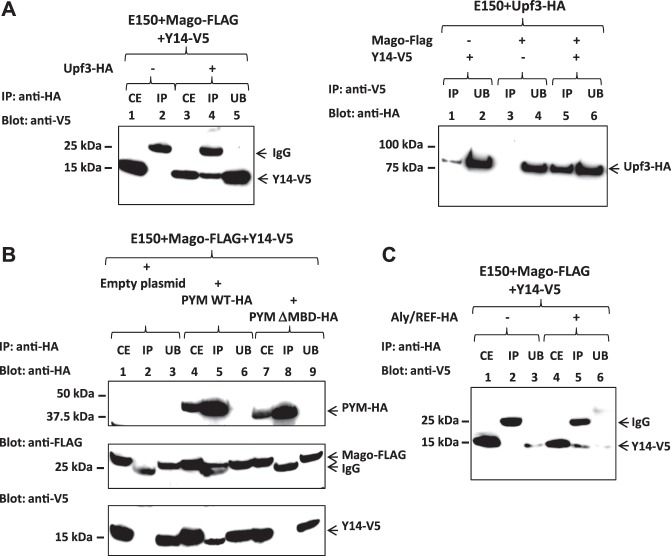
The peripheral factors Upf3, Pym, and Aly/REF associate with Y14 in *Y*. *lipolytica*. (**A**) Co-immunoprecipitation of Y14 with Upf3. Left panel: anti-HA immunoprecipitation was performed on solubilized extracts from membrane-enriched fractions of the double E150 + p*TEF1*-Y14-V5 + p*TEF1*-Mago-FLAG-tagged (lanes 1 and 2) or the triple E150 + p*TEF1*-Y14-V5 + p*TEF1*-Mago-FLAG + pTEF1-Upf3-HA-tagged (lanes 3-5) strains. Immunoprecipitates were resolved by SDS-PAGE (IP, lanes 2 and 4) along with crude extracts (CE, lanes 1 and 3) and unbound samples (UB, lane 5). Proteins were then blotted with anti-V5. Right panel: Y14-V5 from both solubilized samples of the E150 + p*TEF1*-Y14-V5 + pTEF1-Upf3-HA (lanes 1 and 2) and the E150 + p*TEF1*-Mago-FLAG + pTEF1-Upf3-HA (lanes 3 and 4) double tagged strains or the E150 + p*TEF1*-Y14-V5 + p*TEF1*-Mago-FLAG + pTEF1-Upf3-HA (lanes 5 and 6) triple-tagged strain was immunoprecipitated using anti-V5. Immunoprecipitates (IP, lanes 1, 3 and 5) resolved by SDS-PAGE were blotted with anti-HA along with unbound samples (UB, lanes 2, 4 and 6). (**B**) Co-immunoprecipitation of Y14 and Mago with Pym. Anti-HA immunoprecipitation was performed on cytoplasmic extracts of the double E150 + p*TEF1*-Y14-V5 + p*TEF1*-Mago-FLAG-tagged strain (lanes 1 to 3) or two triple-tagged strains: the first expressed a wild type copy of Pym-HA (lanes 4–6) and the second a ΔMBD (Mago Binding Domain) copy (lanes 7–9). Immunoprecipitates (IP, lanes 2, 5 and 8) were resolved by SDS-PAGE along with crude extracts (CE, lanes 1, 4 and 7) and unbound samples (UB, lanes 3, 6 and 9). Samples were loaded on two different gels: a 4–12% gel, which was blotted with anti-HA (upper panel) and a 10–20% gel blotted successively with anti-Flag (middle panel) and anti-V5 (lower panel). The three blots were individually delineated depending on the antibodies used. (**C**) Co-immunoprecipitation of Y14 with Aly/REF. Anti-HA immuno-precipitation was performed on solubilized extracts from membrane-enriched fractions of the double E150 + p*TEF1*-Y14-V5 + p*TEF1*-Mago-FLAG-tagged (lanes 1–3) or triple E150 + p*TEF1*-Y14-V5 + p*TEF1*-Mago-FLAG + pTEF1-Aly/REF-HA-tagged (lanes 4–6) strains. Immunoprecipitates (IP, lanes 2 and 5) were resolved by SDS-PAGE along with crude extracts (CE, lanes 1 and 4) and unbound samples (UB, lanes 3 and 6). Proteins were then blotted with anti-V5. The numbers on the left indicate the molecular masses of the protein standards (Precision Plus ProteinTM Standards from BIORAD) revealed by EZblue staining (Sigma-Aldrich). The revealed proteins are mentioned on the right depending on the strains and the antibodies used.

The interaction of Upf3 with the Y14 component of EJC was previously reported for human proteins^15^. We thus tested the association of YlUpf3 with the Y14 and Mago core proteins using a co-immunoprecipitation approach. We used the *Y*. *lipolytica* strain over-expressing two or three of the Y14-V5, Mago-FLAG, and Upf3-HA-tagged proteins (Supplementary Table S4). We first performed an anti-HA immunoprecipitation of solubilized proteins from membrane-enriched fractions of either the triple-tagged strain or a control strain that did not express the Upf3-HA protein. As shown in Fig. 3A (left panel, anti-V5 blot), Y14 was co-precipitated with Upf3 (lane 4), although unbound Y14 was still detected (lane 5). Y14 can thus be inferred to be a true partner of Upf3, as the presence of Y14 in the precipitate depended on the expression of the HA-tagged Upf3 protein (compare lanes 2 and 4). We next performed the reciprocal experiment with an anti-V5 precipitation on fractions from three strains over-expressing Upf3-HA, either in the presence of Y14-V5 alone, Mago-FLAG alone, or both the tagged EJC core proteins (Fig. 3A, right panel, anti-HA blot). Upf3 was efficiently precipitated when co-expressed with both the epitope-tagged Mago and Y14 proteins (compare lane 5 with lanes 1 and 3). All the Upf3 remained in the unbound fraction and was not detected in the precipitate in the absence of the Y14-V5 protein (lanes 4 and 3).

In the absence of Mago-FLAG, the amount of Upf3-HA co-precipitated with Y14-V5 was lower than in the triple tagged strain extracts (compare lanes 1 and 5). The endogenous *MAGO* and *Y14* promoters are weaker than those used to drive expression of the tagged proteins. Thus, the levels of Mago-Y14 heterodimers were lower in the strain that only over-expressed Y14 (Fig. 2B). This result suggests that the level of bound Upf3 correlated with the level of Y14-Mago complexes.

Overall, these data show that YALI0A10142g is the genuine *Y*. *lipolytica* Upf3 protein, with properties similar to those of its human counterpart. YlUpf3 associates with the complex comprised of the two Y14 and Mago core components and links this complex to the NMD pathway in *Y*. *lipolytica*, as described for the EJC in mammals. We thus investigated the other peripheral components, Pym and Aly/REF.

### The peripheral Pym and Aly/REF components also associate with the *Y*. *lipolytica* Y14-Mago complex

We explored the association of Pym with the Y14-Mago heterodimer by overexpressing an HA-tagged copy of Pym in the previously constructed Y14-V5, Mago-FLAG strain (Supplementary Table S4). An HA-tagged copy of a truncated version of Pym (ΔMBD) was also co-expressed with the Mago- and Y14-tagged proteins to verify that the predicted MBD was indeed involved in Pym binding. We performed anti-HA immunoprecipitations on soluble cytoplasmic fractions from these strains. As shown in Fig. 3B, both forms of Pym-HA, entire or deleted for the MBD, were efficiently precipitated: no further HA signals were observed in the unbound fractions (upper panel, compare lanes 5 and 8 to lanes 6 and 9). Mago and Y14 were detected as co-precipitated partners only when the anti-HA precipitation was performed using samples from the strain expressing the wild type HA-tagged Pym protein (middle and lower panels, compare lanes 5 and 2). Moreover, deletion of the MBD abolished the interaction of Pym with the Y14-Mago core complex. Indeed, neither of the two proteins was co-precipitated with the HA-tagged PymΔMBD protein (middle and lower panels, lane 8). Thus, Pym is a partner of the Y14-Mago heterodimer in *Y*. *lipolytica* and its association with the core complex involves the conserved MBD.

We then tested the association of Aly/REF with the Y14-Mago complex by co-expressing an HA-tagged copy of Aly/REF in the Y14-V5, Mago-FLAG double tagged strain (Supplementary Table S4). Y14 was specifically co-precipitated with the Aly/REF protein (Fig. 3C, compare lanes 5 and 2), thus indicating that this export adaptor protein joins the core complex in *Y*. *lipolytica*.

We thus provide evidence that *Y*. *lipolytica* Mago and Y14 proteins assemble *in vivo* and can be joined by at least three peripheral factors. We next investigated the function of these two components *in vivo* by studying *Δmago* and *Δy14* strains, as each factor has been described to be involved in different steps of mRNA expression (such as export, translation or degradation).

### *Y14* and *MAGO* deletion mutants are viable and show different phenotypes

**Figure 4:**
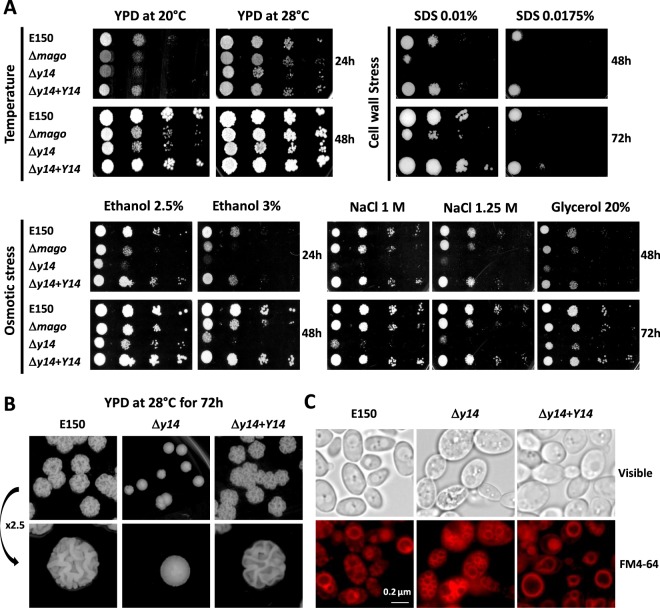
*Δmago* and *Δy14* phenotypes. (**A**) Sensitivity to cold temperature, osmotic, and cell-wall stresses. Serial 1:20 dilutions (5 µL) of the control Δku70LEU2, *Δmago*, *Δy14*, and complemented *Δy14* + *Y14* strains were spread on the surface of YPD plates or YPD plates containing either 0.01% or 0.0175% SDS, 2.5% or 3% ethanol, 1 M or 1.25 M NaCl, and 20% glycerol and further incubated at 20 °C or 28 °C for 24, 48, or 72 h. (**B**) Colony morphology. Cells of Δku70LEU2, *Δy14* mutant and complemented *Δy14* + *Y14* strains were plated on YPD and incubated for 72 h at 28 °C. (**C**) Vacuole visualization. For FM4-64 labeling, exponentially grown cells were resuspended at 40 OD_600_ U/ml in YEA and treated with 40 µM FM 4-64 (Molecular Probes Inc., Invitrogen) for 45 min at 28 °C. YEA rinsed cells were resuspended at 20 OD_600_ U/ml in fresh YEA and further incubated with shaking for 135 min at 28 °C before observation by fluorescence microscopy. Bar: 0.2 µm.

We wished to determine a putative functional role of the *Y*. *lipolytica* Y14 and Mago proteins. We thus first constructed deletion mutants of *Y*. *lipolytica* (Supplementary Table S4). Deletion of *Y14* or *MAGO* resulted in slower growth of the mutant strains than the control strain at low temperatures, such as 20 °C (Fig. 4A). Growth of the mutant strains was only very weakly impaired at the optimal temperature of 28 °C. The only major difference was the smooth appearance of the colonies formed by the *y14* mutant compared to the rough colonies of either the parental or the *Y14* complemented strains (Fig. 4B).

*Mago* and *y14* mutants showed altered resistance to osmotic stress when submitted to ethanol, salt, or glycerol (Fig. 4A). These growth conditions affected *Δy14* more than *Δmago*. Tolerance of the two mutants to cell-wall stress was also less than that of the control strain and again, *Δy14* was more sensitive to SDS than *Δmago*. Insertion of the coding sequence of *Y14* in *Δy14* restored the ability of the complemented strain to grow under the various stressful conditions. At the microscopic level, cells of *Δy14* appeared larger, with a very large number of small vacuoles, whereas cells of Δku70LEU2 (control strain with the same auxotrophy as the mutant strains) generally contained either a single large vacuole or two to four smaller ones (Fig. 4C).

The growth defects we observed under various stressful conditions show that Mago and Y14 have *in vivo* functions and suggest that Y14 may act differently than Mago.

### Transcriptomic analysis of global gene expression in mutant cells

**Figure 5:**
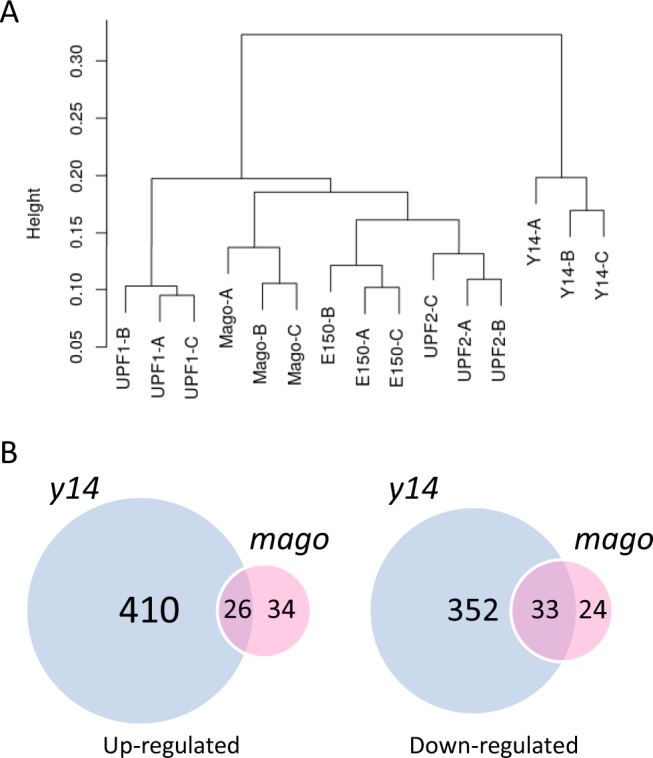
Transcriptome analysis of NMD and EJC mutants. (**A**) Clustering of the 15 libraries from the RNA-seq experiments based on the coordinates of a correspondence analysis (COA) on the count matrix, discretized considering five quantiles. Euclidean distance was used for the distance matrix and the average link method was selected for clustering. (**B**) Venn representation of the number of up-regulated and down-regulated genes in EJC mutants (*Δmago* and *Δy14*) relative to Δku70LEU2.

We investigated the global impact of Y14 and Mago core components at the mRNA level by performing RNA-Seq experiments on *Y*. *lipolytica Δy14* and *Δmago*, as well as Δku70LEU2, *Δupf1*, and *Δupf2*. We produced three replicates and obtained between 18.3 and 29.5 million reads per sample (Supplementary Table S5). Approximately 98% of the filtered reads mapped to the reference genome, with a strong correlation between replicates for protein coding genes (Fig. 5A). We used the DEseq2 package to estimate differential gene expression. We first used a cutoff of 40 reads per kilobase (RPK) to filter genes with low expression. After filtering, 542 protein-coding genes were removed, including *Y14* and *MAGO*, leaving 6,017 genes for analysis. Then, we used a log2 fold change of 1.5 and an adjusted *p-*value of 0.001 as cut-offs. DEseq2 thus identified 821 (436 up and 385 down) and 117 (60 up and 57 down) genes, which showed a significant change in expression relative to the reference strain for *Δy14* and *Δmago*, respectively. Only 26 upregulated and 33 downregulated genes overlapped between the two mutants (Fig. 5B, Supplementary Table S6). These results corroborate the phenotypic differences observed between *Δy14* and *Δmago*. Overall, the deletion of *Y14* had a stronger impact on gene expression than that of *MAGO*, which led to few changes. Interestingly, the levels of 11 mRNAs were higher in *Δmago* than Δku70LEU2, whereas they were lower in *Δy14* (Supplementary Table S6). This result, as well as the extent in the number of differentially expressed genes in *Δy14*, may be a consequence of a modification of gene regulation rather than a direct consequence of Y14 function on mRNA fate. Thus, we investigated changes in splicing in *y14* and *mago* mutants and compared them to those of NMD mutants.

### Intron splicing is affected in *y14* mutant cells

We more precisely characterized the mutant defects by assessing the involvement of *Y*. *lipolytica* Y14 and Mago core proteins in splicing by comparing the retention levels of introns with the expression level of the corresponding genes. We studied *Δy14* and *Δmago*, along with the two NMD mutants, *Δupf1* and *Δupf2*, and Δku70LEU2.

From the structural annotation of the parental strain, E150, we selected 1,372 introns with a minimal size of 50 bp from 1,179 different coding genes. We determined the read counts associated with these introns in the same way as those of the coding genes. Use of the same filtering procedure for low counts as for the coding genes removed 738 introns from the statistical analysis. We thus investigated the 634 remaining introns.

The retention levels of introns assessed with DESeq2 (log2 fold change >1.5 and an adjusted *p-*value < 0.001) led to the identification of 99 introns for which the retention level varied at least once in one of the four studied mutant strains (*Δy14*, *Δmago*, *Δupf1*, and *Δupf2*) from that of the control strain Δku70LEU2.

**Figure 6:**
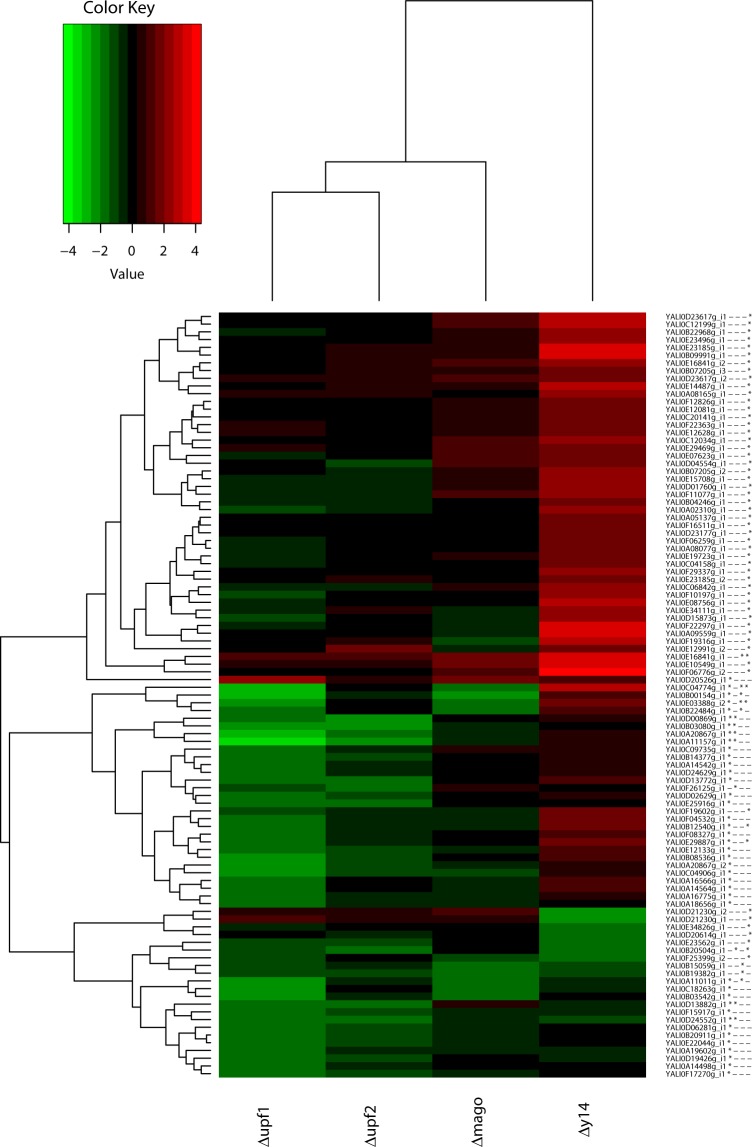
Heatmap of differentially retained introns in NMD and EJC mutants. Heatmap of the fold-changes (in log2 scale) of the 99 DR introns identified from the four pairwise comparisons between the mutant strains and the control strain. Red (green) indicates weaker retention (higher) in the mutant than in the control strain. Star and minus signs on the right, next to the intron names, indicate whether the observed variation is significant or not. Introns and mutants are clustered according to the fold-change values (distance: Euclidean, link: complete).

Clustering of the introns per mutant according to the fold-change values clearly showed a strong trend concerning differences in intron retention (Fig. 6). Most of the introns showing significant differential retention (59 introns) in *Δy14* were mainly retained less than in the control strain (52 red cells). In contrast, *Δupf1* had mostly introns that were more highly retained (40 of 41) than in ΔKu70LEU2. This suggests that the main activity of *Y14* is different from that of *UPF1*. Two genes were tested to validate the data by q-RT-PCR, *YRA1* (YALI0A20867g) and *SUB2* (YALI0A11157g). Both contain significantly differentially retained (DR) introns in *Δupf1* and *Δupf2* (Fig. 6) and previously showed unspliced transcripts subject to NMD^32^. Indeed, intron retention was 27 and 47 times higher in *Δupf1* than in Δku70LEU2 for *YRA1* and *SUB2*, respectively, whereas these values were only 0.58 and 0.48 in *Δy14* (Supplementary Fig. S8). This experimental validation confirms that knocking-out *Y14* has a different impact on mRNA processing than that observed for NMD mutants.

Only a few introns were significantly DR in *Δupf2* and *Δmago* relative to the control strain (eight DR introns for both), with eight and seven highly retained introns in *Δupf2* and *Δmago*, respectively, and one less retained in *Δmago* (Fig. 6). A comparison between DR introns in *Δy14* and *Δmago* suggests that the role of Y14 in intron splicing could be independent of its association with Mago. We further explored this hypothesis by lowering the significance criterion to 1 for the log2 fold-change (in absolute values) instead of 1.5. This led to 199 DR introns (Supplementary Fig. S9). Among them, 106 were less retained in *Δy14*, whereas only 12 were less retained in *Δmago*, 11 of which were in both mutants.

**Figure 7:**
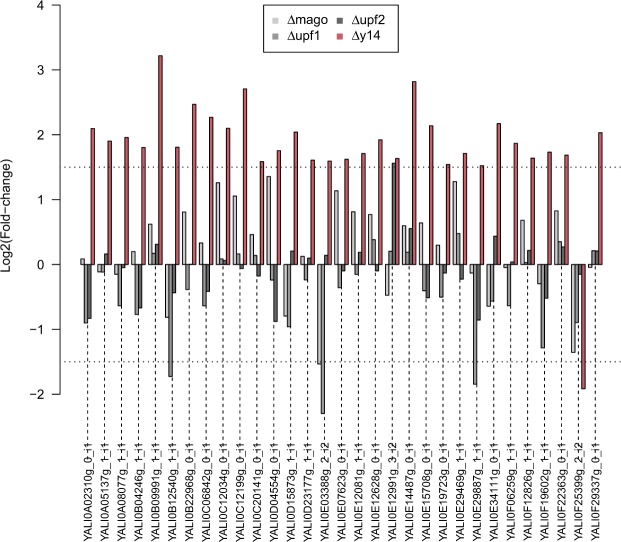
Fold-changes of differentially retained introns in NMD and EJC mutants. Fold-changes (in log2 scale) of the 31 DR introns in Δ*y14* relative to the control strain and for which the corresponding genes are not differentially expressed. In red, the fold-change values between Δ*y14* and Δku70LEU2; in grey scale, the fold-change values for the three other pairwise comparisons. Horizontal dotted lines indicate the fold-change threshold considered to be significant (1.5).

At this step, the level of intron retention was not normalized to the expression of the corresponding genes. The level of intron retention in *Y*. *lipolytica* was low or negligible relative to gene expression, with only a few exceptions. Thus, we eliminated DR introns due to modifications in gene expression by restricting the analysis to DR introns associated with non-differentially expressed genes and assessed whether these introns still followed the observed trends described above. We identified 31 DR introns for *Δy14* (of 59) for which the corresponding genes were not differentially expressed (Supplementary Table S7). The fold-changes (log2 scale) for the four mutants are provided in Fig. 7. Among the 31 introns, 30 were less retained in *Δy14* than in the control strain, confirming that introns in *Δy14* are more efficiently spliced than in Δku70LEU2. Similarly, we examined the DR introns from *Δupf1* that were not associated with differential gene expression (Supplementary Fig. S10). Again, we identified 31 DR introns (of 41). All were more highly retained in the NMD mutant than in the control strain. Three introns were DR in differentially expressed genes in both *Δy14* and *Δupf1* mutants, but with opposite retention levels (YALI0B12540g, YALI0E03388g, and YALI0E29887g).

We attempted to identify correlations between DR introns and various structural characteristics of the genes, such as intron length, transcript length, and the distance between the intron and the 3′ and 5′ ends of the transcript (Supplementary Fig. S11). We did not observe any obvious trends.

## Discussion

### Exon Junction Complex components in budding yeasts

We showed by blast search of the EJC core components against annotated proteomes and yeast chromosomal sequences that eIF4A3 was present in all genomes we studied, but that Mago and Y14 have been lost in at least three yeast families (Saccharomycetaceae, Debaryomycetaceae and Pichiaceae). Indeed, Mago and Y14 were retained in Basidiomycota, Taphrinomycotina, and Pezizomycotina, as well as early-branching Saccharomycotina species, including Yarrowiaceae, and Phaffomycetaceae. This finding shows that the loss of *Y14*, and *MAGO* occurred independently in several lineages, at least two times, *i*.*e*. in the ancestor of Saccharomycetaceae, and before the divergence between CTG and Pichiaceae, unless they have been regained in Phaffomycetaceae (Fig. 1). However, a phylogenetic analysis of fungal *MAGO* and *Y14*, as well as *PYM*, their interacting partner, indicated a clear Saccharomycotina origin of Phaffomycetaceae genes. Moreover, analysis of gene localization revealed that *PYM* was conserved in synteny in both Phaffomycetaceae and *Y*. *lipolytica* (data not shown). These findings rule out the horizontal transfer hypothesis.

In contrast to *MAGO* and *Y14*, the ubiquity of eIF4A3 may be linked to its essential function in the maturation of 18S rRNA, a function highly conserved in eukaryotes, as demonstrated by the complementation of the *S*. *cerevisiae fal1* (null) mutant by its human orthologue^38^.

Our phylogenetic analysis complements the work of Bannerman *et al*., who found a positive correlation between genes of the EJC and intron density^30^. It is commonly accepted that introns of Saccharomycotina yeasts were lost during the early stage of their evolution, with quantitative differences in the various lineages that correlate with their evolution rate^39–41^. Here, we found EJC components in basal species, such as *G*. *candidum*, which contains up to 35% intron-containing genes^42^, and *Y*. *lipolytica*. This correlation may suggest a link between the evolution of splicing and other steps of mRNA metabolism. What is still to be understood is whether there is a causative link between the loss of introns and the regressive mRNA evolution in yeasts.

### EJC core components and peripheral factors constitute an evolutionary package

In this study, we investigated the presence of peripheral factors associated with the EJC core, *i*.*e*. Cwc22, Ibp160, Upf3, Pym, Aly/REF, and ICE1. ICE1, as the core component MLN51, was not found in any of the species studied, suggesting that both genes have been lost early in the evolution of Fungi. Indeed, Bannerman *et al*. reported the presence of MLN51 only in animals. Mammalian MLN51 has two known functions. It stimulates both eIF4A3 helicase activity^43,44^ and mRNA translation through its interaction with the initiation factor eIF3^20^. These functions might be either dispensable in *Y*. *lipolytica* and all other fungal EJC-containing species or carried out by other yet unknown factors. ICE1, a conserved protein known for its role in the initiation step of snRNA gene transcription^45^, was recently identified as a peripheral human EJC factor. Its role is limited to promoting the link between Upf3B and EJC^28^. Here, we show that the EJC-interaction domain of Upf3B is partially conserved in YlUpf3, despite the presence of a validated Y14-Mago-Upf3 complex. The determinants of the protein interaction may thus differ in fungi and either rely on other components or do not require additional factors.

Among the peripheral factors investigated here, two are present in almost all the species we studied, *i*.*e*. Cwc22 and Upf3. These factors have other functions outside the EJC, which may explain their conservation. In *S*. *cerevisiae*, Cwc22 is an essential protein, but intriguingly, the amino-acid residues involved in the interaction of Cwc22 with the EJC component eIF4A3^46^ are conserved almost exclusively in the species containing Mago and Y14. The only exception is *D*. *hansenii*. Substitutions occurred in all other species, suggesting that the selective pressure acting on these amino acids has been lost with the loss of the EJC components. Consequently, substitutions should also occur in *D*. *hansenii*. In fungi, Cwc22 protein sequences are as highly conserved as the Upf3 sequences are divergent. In *Y*. *lipolytica*, Upf3 is completely unusual and recurrent blast and synteny conservation searches were necessary for its identification. Similarly, the identification of *C*. *elegans* SMG-4 (homolog of UPF3) was difficult^47^. More generally, Causier *et al*. reported that UPF3 is poorly conserved; they were unable to identify orthologs in at least five of 13 eukaryotic lineages^48^; the gene is either absent from these genomes or too divergent.

The three last peripheral factors we studied presented a patchy distribution in the phylogenetic tree. Aly/REF and IBP160 play a dispensable role of facilitator, Aly/REF in mRNA export and IBP160 in EJC factor recruitment. Additionally, a role of IBP160 in snoRNP assembly on intron-hosted snoRNA was reported^49^. The loss of IBP160 in Saccharomycetaceae may correlated with the innovation of an alternative snoRNA processing pathway and the concomitant migration of snoRNA from intronic to exonic structures^50^.

Finally, Pym is the only component exclusively present in species with Y14 and Mago components. Up to now, the only known function of Pym was linked to dissociation of the Y14-Mago complex, explaining their concomitant retention through evolution. The patchy distribution of the other peripheral factors suggests that Mago, Y14, and Pym were lost first, followed by progressive loss of secondary factors, which were then under less selective pressure. Thus, the EJC core components and peripheral factors constitute an evolutionary package. The question arises as to the conserved role of this package during evolution.

### The Y14-Mago complex does not trigger NMD in *Y. lipolytica*

Our phenotypic results for the *Δy14* and *Δmago* mutants clearly show that these two proteins play a functional role *in vivo*. Although their role as an EJC, i.e. as a complex associated with RNA as a consequence of pre-mRNA splicing, has not been demonstrated, this is the first evidence that EJC core components act in yeast. Indeed, until our study, fungal Y14 and Mago orthologues were only described in the fission yeast *S*. *pombe*, where they were shown to have no impact on triggering the decay of nonsense containing mRNAs^31^.

Our RNA-seq data designed to establish a putative link between EJC components and NMD, indicate that Mago and Y14 are not required by *Y*. *lipolytica* for the degradation of unspliced PTC-containing transcripts by the NMD machinery, contrary to the EJC model described in metazoans. Although YlUpf3 associates with the Mago-Y14 heterodimer, as in mammals, this association appears to be dispensable for triggering the NMD process. This suggests that recognition of PTC and subsequent degradation of the unspliced mRNA occur independently of the presence of any EJC. This has been described in the budding yeast *S*. *cerevisiae*, in which transcripts containing a PTC are subject to NMD, despite the absence of the EJC, *i*.*e*. without recruitment of Upf1 and Upf2 through EJC-bound Upf3. In this yeast, the important determinant for distinguishing PTC from a normal stop codon is the distance between the nonsense codon and the 3′ end, in accordance with the “faux” 3′-UTR model^51^. Similarly, NMD was shown to be independent of EJC in *D*. *melanogaster*, *C*. *elegans* as in *S*. *pombe* in which degradation of PTC-containing mRNA is not affected by the deletion of *MAGO*^52,53^. Recent publications now report that the EJC is not linked to NMD in other unicellular eukaryotes^54,55^.

### An EJC-independent role of Y14 in *Y*. *lipolytica*

Y14 and Mago assemble in a complex, but we showed that knocking them out in *Y*. *lipolytica* had different consequences both at the phenotypic and mRNA level. The expression of many genes was much more strongly affected in *Δy14* than *Δmago*. Moreover, the levels of some mRNAs were higher than in the control strain in one mutant, whereas it was lower in the other. This may be an indirect consequence due, for example, to modification in the expression of transcriptional regulators. Finally, only 59 of the 6,559 genes in the E150 genome were co-differentially expressed in *Δy14* and *Δmago*. These results suggest two hypotheses. First, the involvement of Y14 and Mago in the function of the complex they form may differ. Inside the core complex, Y14, which contains an RNA binding motif, may carry out the main functional role, whereas Mago may have an auxiliary function, such as a chaperone. Indeed, we observed that the levels of Y14 were clearly higher when Mago was co-expressed, suggesting that the protein is stabilized through association with its partner. Consequently, Y14 could still play its role in the absence of its chaperone, notably through the recruitment of peripheral factors as the main interacting partner. The alternative hypothesis is that Y14 may have an additional function apart from the complex that does not require its interaction with Mago. Such a putative role is yet to be determined.

EJC plays a predominant role in splicing^56–58^ and alternative splicing^59–61^. We thus examined the effect of knocking-out *Y14* and *MAGO* on intron splicing. Once again, *Δy14* showed a stronger effect, with higher splicing efficiency than the control strain. We thus hypothesized that Y14 might interfere with pre-mRNA maturation and/or unspliced mRNA degradation in two ways. The first possibility is that Y14 binds to nascent transcripts and competes with the spliceosome, resulting in reduced splicing efficiency. Thus, in the absence of *Y14*, the spliceosome machinery may act more efficiently on the pre-mRNA and the level of spliced mRNA increases without affecting the overall transcript levels. Alternatively, Y14 may favor the retention of unspliced mRNA in the nucleus through its interaction with a component of the spliceosome, as was shown for *Caenorhabditis elegans*^62^. In the absence of *Y14*, unspliced mRNA could escape from the nucleus and rapidly be targeted and eliminated by NMD in the cytoplasm. Therefore, overall transcript levels may be reduced as a function of the number of transcripts exported from the nucleus. In this hypothesis, the number of intronic reads counted for the control strain should correspond to mRNA sequestered in the nucleus. Both mechanisms may operate in the cell depending on the intron characteristics, such as the length, position and number in the mRNA, and conservation of intron boundaries.

## Conclusions

Here, we describe, for the first time, the identification in budding yeasts of three core components of the EJC. By tracing the evolution of these components in Ascomycota, we showed that their loss in *S*. *cerevisiae* and related species was concomitant with the loss of peripheral factors that join EJC in metazoans, either to mediate its assembly or trigger downstream processes. We used biochemical approaches, using *Y*. *lipolytica* as a model, to show that Mago and Y14 have retained the capacity to form heterodimers and that they constitute a platform for the binding of the peripheral factors Upf3, Aly/REF and Pym, as in metazoans. We explored the functional role of Mago and Y14 by characterizing knockout mutants at the phenotypic and transcriptomic level. The results showed that Mago and Y14 may be involved in mRNA metabolism and that Y14 may play a role in splicing, either by interfering with pre-mRNA splicing factors or by retaining mRNA molecules in the nucleus before their export and translation. These findings contribute to our understanding of EJC evolution and promote the use of yeast as a new model to decipher the function of the EJC.

## Materials and Methods

### Strains and culture media

The *Y*. *lipolytica* strain E150/CLIB122 (*MatB*, *his-1*, *ura3-302*, *leu2-270*, *xpr2-322*) was used as a backbone for gene deletion and for expression of epitope-tagged proteins. *S*. *cerevisiae* strain PJ694α (*MAT*α, *trp1*-901, *leu2*-3,112, *ura3*-52, *his3*-200, *gal4*Δ, *gal80*Δ, *LYS2::GAL1-HIS3*, *GAL2-ADE2*, *met2::GAL7-lacZ*) was used for the two-hybrid experiment^63^. Overall, strains used and constructed in this study are listed in Additional File 11.

Yeast strains were usually grown at 28 °C in YPD (10 g/L yeast extract, 10 g/L bacto-peptone, 10 g/L glucose) with 12 g/L pastagar (Difco, Detroit, USA) in solid medium. For *Y*. *lipolytica* transformant selection, minimal medium (YNB) was made of 1.7 g/L yeast nitrogen base without amino acids and ammonium sulfate (YNBww; Difco), 5 g/L NH_4_Cl, 50 mM phosphate buffer (pH 6.8) and 10 g/L glucose, with 14 g/L pastagar (Difco) in solid medium. Minimal medium was supplemented, as required, with 2 g/L casamino acids, 100 mg/L uracil, 200 mg/L histidine or 400 mg/L leucine. YEA (Yeast extract 5 g/L, glucose 10 g/L) supplemented with 75 mg/L of Hygromycin was used for selection of *Y*. *lipolytica* transformants. *S*. *cerevisiae* transformants were selected on minimal medium made of 6.7 g/L yeast nitrogen base without amino acids and with ammonium sulfate (Difco) supplemented with histidine, methionine, uracil, and adenine at a final concentration of 100 mg/L.

For phenotypic analysis, parental, mutant and complemented strains were grown overnight in YEA at 28 °C and diluted to OD_600nm_ of 0.5 U/mL. 5 µL drops of serial 1:20 dilutions were spotted either on YPD plates or YPD containing SDS 0.01% and SDS 0.0175%, Ethanol 2.5% and 3%, NaCl 1 M and 1.25 M and Glycerol 20%,. Plates were incubated for 24, 48 and 72 h at 20 °C or 28 °C. For vacuole observation, 10 OD_600nm_ Units of exponentially grown cells were resuspended in 250 µL of YEA (40 OD U/mL). 25 µL of a 400 µM FM4-64 solution diluted from a 16 mM stock solution in DMSO were added and cells were further incubated for 45 min at 28 °C. Cells were then washed twice with YEA and incubated for 135 min at 28 °C before microscopic observation using an Olympus System Microscope Model BX51 with 512-nm excitation and 565-nm emission filters on an Olympus 100X oil immersion objective with 10x oculars.

### *In silico* identification of EJC components and peripheral factors

In order to find homologues of Mago, Y14, Upf3, Pym and Aly/REF in a set of yeast and fungal genomes, we proceeded by BLASTp, either on the GRYC website (http://gryc.inra.fr) or at NCBI (https://www.ncbi.nlm.nih.gov/) using *Y*. *lipolytica*, *S*. *cerevisiae* and *Cyberlindnera fabianii* proteins as queries. As Y14 and Mago are small proteins that could be missed during the genome annotation process, homologues were also searched by tBLASTn on chromosomal sequences.

A first BLASTp search of *S*. *cerevisiae* Upf3 sequence against *Blastobotrys adeninivorans* proteome identified ARAD1D01606g as a putative homolog of *UPF3*. The product of this gene has a conserved Smg4/UPF3 domain (pfam03467 domain) with a low but significant E-value (2.42e-12). Its amino acid sequence was then used for a BLASTp search against the proteome of both *Y*. *lipolytica* and *Candida hispaniensis*, which is a species closely related to *Y*. *lipolytica* (Fig. 1), and whose genome has been sequenced and annotated in the lab. A single hit was found in *C*. *hispaniensis*, OLHI0D06370g, but none in *Y*. *lipolytica*. OLHI0D06370g also had a conserved pfam03467 domain (E-value of 4.56.e-08). YALI0A10142g was identified as a putative homolog of OLHI0D06370g with 27% identity covering 80% of the sequence length.

### Phylogenetic analyses

Phylogenetic analyses were conducted on a set of 42 yeast and fungi that represent the dikarya (Supplementary Table S1). The tree was based on the concatenation of different proteins. A first set of 104 protein sequences was chosen with the following criteria: i) genes are singleton in all of the species considered, ii) alignment of the protein sequences with MAFFT v6.903b^64^ subsequently cleaned with Gblocks v.0.91b^65^, in which the options −b3 = 12 and −b4 = 6 were applied, represents at least 70% of the initial alignment, iii) the resulting alignment is longer than 100 amino acids. These criteria were applied to the 34 Saccharomycotina genomes. Then, homologues in 8 strains of Pezizomycotina, Taphrinomycotina and Basidiomycota were searched by BLASTp using *Y*. *lipolytica* proteins as baits. A total of 14 groups of homologues were discarded due to missing data or non-singleton members. Finally, 90 MAFFT alignments were performed and further concatenated, leading to a final 32,405-residue alignment. Phylogenetic trees were constructed by maximum likelihood, with PhyML v3.0^66^ and a LG substitution model corrected for heterogeneity between sites by a Γ-law distribution, with four different categories of evolution rates. The proportion of invariable sites and the α-parameter of the Γ-law distribution were optimized according to the data. Branch support was estimated using SH-like approximate likelihood-based method from PhyML software.

### Other bioinformatics analyses

Local synteny around *YlUPF3* was analyzed by BLAST search of YALI0A10142g and YALI0A10164g homologues, followed by chromosomal localization in genomes listed in Supplementary Table S1. Multiple sequence alignments of NMD factors, EJC compounds and peripheral proteins were performed with multalin^67^. The NLStradamus server was used to determine the presence of a nuclear localization signal^68^.

### Two-hybrid

The *Y14* (YALI0E02530g) and *MAGO* (YALI0D26664g) open reading frames were amplified with primer pairs Y14_171_*Nco*I-Y14_497_*Bam*HI (primers 1–2) and Mago_0_*Nco*I-Mago_585_*Bgl*II (primers 3–4) and cloned in frame with the DNA binding domain of Gal4p in pAS2ΔΔ (*TRP1*, Amp^r^) and with the Gal4p-activating domain in pACT2 (*LEU2*, Amp^r^), respectively. After restriction of the two amplified fragments with *Nco*I/*Bam*HI or *Nco*I/*Bgl*II, the digested products were ligated with the pAS2ΔΔ or pACT2 vector that had been restricted with *Nco*I/*Bam*HI. The resulting recombinant plasmids were used in *S*. *cerevisiae* transformations. A complete list of all the primers used in this study is available in Supplementary Table S8.

### Y14, Mago, Pym, Aly-REF and Upf3 epitope-tagging

In order to express the coding sequences at a high level and constitutively in *Y*. *lipolytica* cells, each open reading frame was cloned in expression vectors containing the *TEF1* promoter^69^. Since a stretch of acidic amino acid is present at the C-terminus of Y14 (RSRSPGRRR) that might be a nuclear import signal, we decided to add the V5 tag (GKPIPNPLLGLDST) just downstream from the ATG codon. For this purpose, two pairs of primers were designed (5–6 and 7–8). The two Y14_3_V5F and Y14_4_V5R oligonucleotides (6 and 7) hybridized over 18 nucleotides and contained the V5 tag coding sequence. No predictive localisation sequence was characterized at the end of the Mago protein, the FLAG tag (DYKDDDDK) was thus added upstream of the STOP codon using primers 9–10. In the same way, the HA tag (YPYDVPDYA) was added upstream of the STOP codon of the Pym protein, (YALI0B01562p) using the two primers 11–12. Two other primers were designed (13–14) and used in two separate amplifications with respectively primers 11 and 12 to create a Pym copy deleted of the twelve amino-acids that correspond to the Mago Binding Domain (MBD). The same vector, which contains the HA tag downstream a unique restriction site was then used to clone in frame successively the *ALY-REF* (YALI0F07909g) and the *UPF3* (YALI0A10242g) coding sequences after amplification with the two couples of primers 15–16 and 17–18. The amplified sequences were cloned into three different plasmids containing *LEU2*, *URA3* or *HPH* selection genes using the *Bam*HI and *Avr*II restriction sites. The recombinant plasmids were digested with the *Not*I enzyme before *Y*. *lipolytica* transformation. Integration of the expression cassette was confirmed using primers TEF1_Prom_F and LIP2_Term_R (19–20). The transformed strains thus expressed two copies of the coding sequence, the wild type copy under the control of its own promoter and the epitope-tagged construct under the *TEF1* promoter.

### Preparation of soluble protein fractions and immunoprecipitation

*Y*. *lipolytica* cells were grown overnight in YEA and harvested by centrifugation. TE-washed cells were resuspended in lysis buffer (Phosphate Buffer Saline) containing protease inhibitors (complete EDTA-free from Roche) and disrupted through addition of glass beads in a Bead-Beater 24™ (MP Biomedicals, California, USA) four times for 20 s each with 5 min incubations in ice between each round. Subsequently, the lysate was collected and centrifuged at 4,000 g for 10 min at 4 °C to collect a ‘low-speed supernatant’ (S_4000_). To obtain either a cytosolic or an organelle-enriched fraction, the low-speed supernatant was further centrifuged at 17,000 g for 30 min at 4 °C. This ‘high-speed supernatant’ (S_17000_) that mainly contains the soluble cytosolic proteins was used to perform immunoprecipitation on cytosolic extracts. To solubilise nuclear proteins, the high-speed pellet (C_17000_) that consists mainly of intracellular organelles was resuspended in lysis buffer containing 2% TritonX100 and 0.5% SDS and incubated for 30 min at room temperature to solubilise the membranes and liberate the luminal content of the organelles. After a 15 min centrifugation at 17,000 g, the non-solubilized material was discarded and the supernatant further conserved at −20 °C for immunoprecipitation on nuclear fractions. For immunoprecipitation, 500 μL of these samples were diluted five times in PBS and incubated either with anti-HA agarose (SIGMA) or anti-V5 agarose (SIGMA) for 4 hours at 4 °C. Agarose beads were washed three times with 15 mL of PBS and precipitates were eluted in 100 μL of sample buffer (100 mM Tris-HCl pH 6.8, 2% 2β-mercaptoethanol, 20% glycerol, 4% SDS, 0.02% Bromophenol blue) for 15 min at 75 °C.

### Western blot analysis

Extracted or immunoprecipitated proteins were separated by SDS-polyacrylamide gel electrophoresis on NuPAGE^R^Novex Bis-Tricine 4–12% or Tris-Glycine 10–20% pre-cast gels (Invitrogen, Cergy Pontoise, France) in NuPAGE^R^Novex MOPS buffer (Invitrogen, Cergy Pontoise, France) using the XCell Mini-Cell system (Invitrogen, Cergy Pontoise, France). The proteins were transferred onto nitrocellulose membranes (Amersham Protran) for Western blot analysis. Following transfer, membranes were rinsed in PBS and blocked in PBST (PBS plus 0.1% Tween 20) + 2% skimmed milk from Difco for one hour at room temperature. The membranes were then incubated overnight at 4 °C in PBST containing a 1:5000 dilution of either a monoclonal anti-V5 antibody (Invitrogen, Cergy Pontoise, France) or a polyclonal anti-HA antibody (MP BioMedicals, France). After 3 washes in PBST, one-hour incubations in the presence of peroxidase-conjugated anti-mouse IgG antibodies or peroxidase-conjugated anti-rabbit IgG antibodies (GE Healthcare) were performed. The membranes were washed three times before detection of the signal using the Enhanced Chemi Luminescence (ECL)^+^ detection system (GE Healthcare).

### Gene deletion

*KU70* (YALI0C08701g) deletion was performed using the deletion cassette *ylKU70* PUT purified on agarose gel (QiaQuick gel extraction kit, Qiagen, Courtaboeuf, France) from *Not*I-digested plasmid JME1264^70^. Transformation of *Y*. *lipolytica* E150 by the lithium acetate method was performed as described previously^71^. Ura + transformants were selected on YNB supplemented with casamino acids. Gene deletion was checked by PCR amplification of purified DNA, with primers Ku70Ver1 and Ku70Ver2 (21–22), followed by an *Eco*RV restriction.

*UPF1* and *UPF2* deletions in ∆*Ku70* were performed as previously described^32^. Deletion cassettes for *MAGO* (YALI0D26664g) and *Y14* (YALI0E02530g) were constructed by a two-step PCR amplification. A first PCR was performed for the promoter (P) and terminator (T) regions of each gene and for the *URA3* or *LEU2* markers with primers (23 to 28, 31 to 36, 39–40 and 41–42 respectively). Primers MagoP2ura, Y14P2ura, MagoT1ura, and Y14T1ura have a 3′-end complementary to 22 bp at the extremities of the *URA3* PCR product, and similarly, MagoP2leu, Y14P2leu, MagoT1leu, and Y14T1leu to the extremities of the *LEU2* PCR product. All PCR products were purified from agarose gel with the QiaQuick gel extraction kit (Qiagen) to remove residual primers. A second PCR amplification was performed with P, T and the *URA3* or *LEU2* marker with the following program: 2 min at 94 °C, then 5 cycles of 30 s at 94 °C, 30 s at 60 °C, 1.5 min at 72 °C, followed by 25 cycles of 30 s at 94 °C, 30 s at 60 °C, 3 min at 72 °C. Primer pairs 23–28 or 31–36 were added after the 5 first cycles. The resulting PCR products were purified from agarose gel and about 400 ng of the purified cassettes was used to transform Δ*ku70*. Transformants were selected on YNB complemented with histidine and leucine or uracil depending on the marker used. Gene deletion was controlled by PCR amplification with primers external to the disruption cassette, i.e. upstream from P1 primers and downstream from T2 primers (Supplementary Table S8).

### *Y14* complementation

In order to complement the *Δy14* mutant, a *Y14* copy expressed under its own promoter was constructed by a two-step PCR amplification. Two primers, 43–44, were designed and used with the two previous 6–7 oligonucleotides to first amplify 1 kb of the promoter of *Y14* (43 and 7) and the *Y14* open reading frame followed by 265 pb of the terminator (44 and 6), respectively. In a second step, the two fragments were hybridized using the 18 nucleotides overlapping sequences of 6 and 7 and the complete sequence was amplified using the two external primers 43 and 44. The amplified sequence was cloned at the *Cla*I/*Sac*II sites of a *Y*. *lipolytica* integrative plasmid after a *Cla*I/*Sac*II restriction. The *LEU2* gene was replaced after a *Cla*I/*Stu*I restriction and cloning of a *Cla*I/*Eco*RV restriction fragment containing the *HPH* resistance gene. The resulting plasmid was cuted by *Xho*I before transformation of the *Δy14* mutant.

### Extraction of RNA, DNase treatment and reverse transcription

To prepare RNA samples, cells were grown at 28 °C in YPD medium at a starting OD_600_ of 0.25 to an OD_600_ between 1.5 and 2.0 (4.5 to 6.10^7^ cells/mL). 10^8^ cells were harvested by centrifugation. The cell pellet was immediately frozen in liquid nitrogen and stored at −80 °C. Three biological replicates were performed. The RNeasy Midi Kit (Qiagen, Courtaboeuf, France) was used to extract total RNA from cells. Purified RNA was quantified at 260 nm using a NanoDrop ND-1000 spectrophotometer (NanoDrop Technologies, Wilmington, DE). The quality of the RNA was analyzed with a 2100 Bioanalyzer (Agilent, Palo Alto, CA) using RNA 6000 Nano chips according to the manufacturer’s instructions. For q-RT-PCR analyses, DNase treatment was performed with a Turbo DNA-free kit (Invitrogen, Cergy Pontoise, France) according to the manufacturer’s instructions. 500 ng of DNase-treated RNA were then converted to cDNA with a SuperScript Vilo cDNA Synthesis kit (Invitrogen, Cergy Pontoise, France). cDNA was then diluted 1:5 in RNase-free water for qPCR analysis. Absence of DNA contamination, in RNA samples, was confirmed with non-reverse-transcribed assays.

### Quantitative PCR

Oligonucleotide primers were designed using LightCycler probe design software (v1.0; Roche Applied Science, Mannheim, Germany) and synthesized by Eurogentec (Seraing, Belgium). Two sets of primers were designed. One set was used to amplify the target transcripts in which the intron has been spliced while another set of primers was used to amplify transcripts with unspliced intron. The primers 45 to 54 were used for quantitative PCR.

Quantitative PCR was performed in a LightCycler 1.5 instrument (Roche, Meylan, France) using the LightCycler FastStart DNA Master^Plus^ SYBR Green I kit (Roche, Meylan, France), according to the instructions of the manufacturer. The thermocycling program consisted of an initial denaturation at 95 °C for 8 min, followed by 45 cycles of denaturation (95 °C, 10 s), annealing (59 °C, 7 s), and extension (72 °C, 6 s). Fluorescence was measured (530 nm) at the end of each extension. After real-time PCR, a melting-curve analysis was performed by measuring fluorescence during heating from 65 to 95 °C at a transition rate of 0.1 °C/s.

The threshold cycle (*CT*) values were determined with LightCycler software (version 3.3), using the second derivative method. Standard curves were generated by plotting the *CT* values as a function of the initial RNA concentration log. PCR efficiency (*E*) was then calculated using the following formula: *E* = 10^−1^/slope. The Pfaffl method (Pfaffl, 2001) was used to calculate the fold change in transcript abundance normalized to the *YlTFC1 (*YALI0E14663g) gene encoding RNA polymerase III transcription initiation factor IIIC subunit. *YlTFC1* was used as internal control since it was shown to have constant expression level by RNA-seq analysis. Three independent replicates, prepared from independent biological samples, were analyzed. A student’s test was performed (two tailed distribution), using GraphPad Prism, in order to compare each sample values with wild-type values of the same group (retained or spliced) and *p*-values were calculated.

### RNA-Seq analysis

Library preparation and Illumina sequencing were performed at the Ecole normale supérieure Genomic Platform (Paris, France). Messenger (polyA+) RNAs were purified from 1 µg of total RNA using oligo(dT) beads from Illumina. The libraries were prepared using the strand non-specific RNA-Seq library preparation TruSeq RNA Sample Prep Kits v2 (Illumina). Quality of the libraries was determined on a Bioanalyzer device, using the DNA 1000 kit from Agilent. The libraries were quantified on the Qubit system from Invitrogen using the dsDNA HS Assay Kit and loaded on two flowcell lanes and subjected to 50 bp single-read sequencing on a HiSeq. 1500 device.

RNA-Seq data were first cleaned and mapped against the reference genome of *Y*. *lipolytica* E150 (Genome annotation available on http://gryc.inra.fr). These preliminary steps were performed with Trimmomatic tools (v. 0.33)^72^ that clip technical adaptors and remove low quality regions from reads. Then, Tophat2 aligner tool (v. 2.1.0)^73^ was used to map reads against the reference genome of E150. Last, two count matrices were built with HTSeq-Count (v. 0.6.0)^74^, using the mode “intersection-strict”, the first one considering all the complete coding gene loci, the second one considering only intron positions. To build this latter, a dedicated GFF file containing only the intron coordinates from the E150 reference annotation (see above) was prepared in order to maximize the number of reads attributed to introns. Indeed, considering both the introns and the exons at the same time in HTSeq-Count would yield to classify all reads that overlap exon/intron boundaries as ambiguous.

Statistical analysis was performed on the two count matrices under the R environment (v 3.3.3) [www.r-project.org] with the package DESeq2 (v. 1.20)^75^ and different “in-house” R scripts. First, read count matrix on coding genes was treated. A preliminary filtration of genes with low counts (<=40 RPK, Read Per Kilobase, in at least 10 out of the 15 libraries) to ensure the statistical reliability of the analysis. Genes with low counts were manually investigated to identify high expression contrast between the compared mutants. Read counts of the remaining genes were treated with the DESeq2 procedure. We considered all the pairwise comparisons, Δku70LEU2 versus mutant strains (4 comparisons). Two criteria were considered for a differentially expressed gene: 1) the adjusted *p*-value of the DESeq2 statistical test must be below 0.001; 2) The fold change, expressed in log2, between the scaled mean read counts of Δku70LEU2 and the scaled mean read counts of the considered mutant strain must be higher than 1.5 in absolute value. The read count matrix based on introns was investigated similarly, except the normalization step. Indeed, because the number of reads that mapped on introns corresponds to a very limited subset of the total number of reads, it is more reliable to normalize the read counts considering the libraries’ depths of the complete dataset rather than the depths of the limited subset. Determination of the DR introns between strain Δku70LEU2 and the four mutant strains was done using the same criteria with the same thresholds.

### Equipment and settings

Digitalized images for Figs 2, 3 and 4 were treated accordingly to editorial integrity policies.

## Electronic supplementary material

SupplementaryFiguresS1–11_TablesS1S3–5S8

SupplementaryTableS2

SupplementaryTableS6

SupplementaryTableS7

## Data Availability

The RNA-Seq reads have been deposited at the European Nucleotide Archive (ENA) under the study project PRJEB26722. The processed data tables and results from statistical tests (Supplementary Tables S5–S7 and Fig. S8) are included as additional files for this article.
