# Supplementary material for: Exon junction complex components Y14 and Mago still play a role in budding yeast
Source: Sci Rep. 2019 Jan 29;9:849. doi: 10.1038/s41598-018-36785-3 (PMC6351623; doi:10.1038/s41598-018-36785-3)
Supplement: Supplementary file 1 — SupplementaryFiguresS1–11_TablesS1S3–5S8 [file 41598_2018_36785_MOESM1_ESM.pdf]

Exon junction complex components Y14 and Mago still play a role in budding yeast

Anita Boisramé, Hugo Devillers, Djamila Onésime, François Brunel, Juliette Pouch, Mathieu Piot, and Cécile Neuvéglise

Supplementary Figures and Tables

Supplementary Figure S1. Sequences and alignment of Mago proteins.

Supplementary Figure S2. Sequences and alignment of Y14 proteins.

Supplementary Figure S3. Sequences and alignment of eIF4A3 proteins.

Supplementary Figure S4. Sequences and alignments of CWC22 proteins.

Supplementary Figure S5. Sequences and alignment of Upf3 proteins.

Supplementary Figure S6. Sequences and alignment of Pym proteins.

Supplementary Figure S7. Sequences and alignment of Aly-REF proteins.

Supplementary Figure S8. Intron retention in NMD and EJC mutants.

Supplementary Figure S9. Heatmap of the fold-changes (in log2 scale) of the 199 differentially retained introns between the mutant and control strains.

Supplementary Figure S10. Fold-changes (in log2 scale) of the 31 DR introns between Δ*upf1* and the control strain and whose corresponding genes are not differentially expressed.

Supplementary Figure S11. Intron structural parameters with respect to intron retention categories.

Supplementary Table S1. Genome sequences and annotation used in this study.

Supplementary Table S3. Percentages of identity and similarity between *Y. lipolytica* Mago, Y14 and Pym proteins and proteins of Yarrowiaceae and Phaffomycetaceae families.

Supplementary Table S4. Yeast strains used in this study.

Supplementary Table S5. Quantitative data on RNA-Seq experiments.

Supplementary Table S8. List of primers used in this study.

References for supplementary files

Supplementary Figure S1: Sequences and alignment of Mago proteins

A. Sequence alignment of Mago in model genomes

```
>HsMago [Homo sapiens] (NP_002361.1)
MESDFYLRYYVGHKGKFGHEFLEFEFRPDGKLRYANNSNYKNDVMIRKEAYVHKSVMEELKRIIDDSEITKEDDA
LWPPPDRVGRQELEIVIGDEHISFTTSKIGSLIDVNQSKDPEGLRVFYYLVQDLKCLVFSLIGLHFKIKPI

>DmMago [Drosophila melanogaster] (AAF46677.1)
MSTEDFYLRYYVGHKGKFGHEFLEFEFRPDGKLRYANNSNYKNDTMIRKEAFVHQSVMEELKRIIIDSEIMQEDD
LPWPPPDRVGRQELEIVIGDEHISFTTSKTGSLVDVNRSKDPEGLRCFYYLVQDLKCLVFSLIGLHFKIKPI

>CeMago [Caenorhabditis elegans] (NP_493025.1)
MSGEEEKAADFYVRYYVGHKGKFGHEFLEFEFRPNGSLRYANNSNYKNDTMIRKEATVSESVLSELKRIIEDSEI
MQEDDDNWPEPDKIGRQELEILYKNEHISFTTGKIGALADVNNSKDPDGLRSFYYLVQDLKCLVFSLIGLHFKIK
PI

>SpMago [Schizosaccharomyces pombe 972h-] (NP_596666.1)
MSDFYVRYYSGHHGRFGHEFLEFDYHSDGLARYANNSNYRNDSLIRKEMFVSELVLKEVQRIVDDSEIIKESDES
WPPENKDGKQELEIRMNGKHIMFETCKLGSLADVQNSDDPEGLKVFYYLIQDLKALCFSLISLNFKLRPVKN

>YlMago [Yarrowia lipolytica CLIB122] (XP_503327.1)
MAEEEEYSIATRPGEDVQMEEELEVVEPEDDLIEKPLSLRVDAKEEAFYLQYYSGHTGRFGHEFLEFDFQLLDQG
RSALLRYANNSNYKNDTLIRREVAVSPVVVKVLKKMIKESEILKENDAKWPPKNRDGKQELEIKFANYHIAFETG
RIGSLSDVQRSDDPEGLRTFYYLVQDIKALVFSLVGLHFKIKPI
```

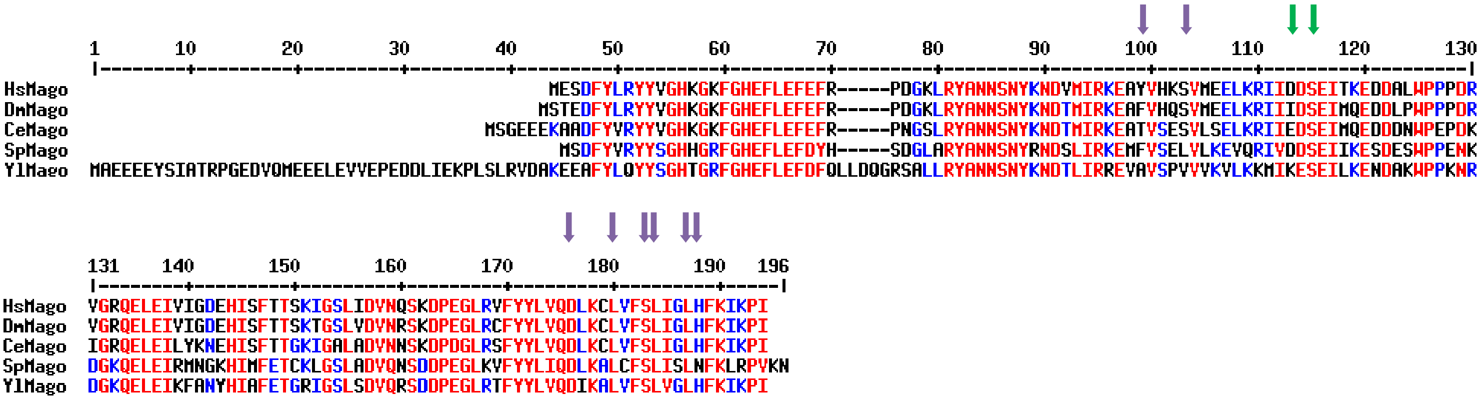

Alignment was performed with the Multalin server (http://multalin.toulouse.inra.fr/multalin/)

⬇ (purple arrow) Amino acid residues involved in the interaction of the *D. melanogaster* Mago protein with the Y14 protein (V_{53}, V_{57}, D_{129}, L_{133}, S_{136}, L_{137}, L_{140}, H_{141}) (Fribourg *et al.;* 2003)

⬇ (green arrow) Amino acid residues involved in the interaction of the *H. sapiens* Mago protein with the Upf3 protein (D_{66}, E_{68}) (Buchwald *et al.;* 2010)

B. Sequence alignment of Mago in Saccharomycotina

```
>YlMago [Yarrowia lipolytica CLIB122] (XP_503327.1)
MAEEEEYSIATRPGEDVQMEEELEVVEPEDDLIEKPLSLRVDAKEEAFYLQYYSGHTGRFGHEFLEFDFQLLDQG
RSALLRYANNSNYKNDTLIRREVAVSPVVVKVLKKMIKESEILKENDAKWPPKNRDGKQELEIKFANYHIAFETG
RIGSLSDVQRSDDPEGLRTFYYLVQDIKALVFSLVGLHFKIKPI

>ChMago [Candida hispaniensis CBS 9996] (OLHI0E23200g)
MENVGELEGQAVTQTESKSESQSELQTEIQPKPQTEPQVDSQIDLHASQGNPQADSNVDSESASHTEQHTAPDSH
LDSYPQTEIDNHREISTNTKRINIDVDRKEDEFSIHYYAGHQGRFGHEFLEFSVEVMGNGSFALLRYANNSNYKN
ETLIKREVAISPLVIKGFKKIICESEILRESDKDWPERNRDGKQELQVKIGQYSCNLETCRIGSLAEIRDTLDPE
GLRVFYYLVQDLKAFLFSLISLHFKIKPI
```

>BaMago [Blastobotrys adeninivorans LS3] (ARAD1D25212g)
MEVDLEGKELGTRAPMSSDLDAGDKNTDSGVESGERMLEDKEASQIPIDISKADDAFYVRYYSGHQGKYGHEFLE
FDLRAQANGRFATLRYANNSNYRNDTLIKKETMVSPSVIKEFKKIIADSEILKEDDERWPEKNRDGKQELEVRVG
KYHISFETAKIGSLNDVQNSDDPEGLRVFYYLVQDLRAFVFSLISLHFKIKPI

>GcMago [Geotrichum candidum CLIB 918] (emb|CDO57545.1)
MTSVINKHQSEPLYIRYYAGHQGRYGHEFLEFDIRVQDDGKSAILRYANNSNYKNDNLIRKEATISPTVVEEIRR
IITESEIVHEDDKKWPPKNRDGKQELELRLGSFHISFETAKIGSLSDIQNSDDPEGLRVFYYLVNDLKAFVFSLI
SLHFKIKPI

>WcMago [Wickerhamomyces ciferrii NRRL Y-1031 F-60-10] (this study)
MSQEENKSIKNAEIDVNDAGQAFYLRYYSGHQGRFGHEFLEFDVELNEEKTSALVRYTNNSNYRRDDLIRKEFTI
SVAVVKQLKQIISDSEILKEDDSKWPEKNADGKQELEIKSGRYHISFETTKIGSLTDVRNSDDPEGLRVFYYFVQ
DIKALIFSLINLHFKVKPL

>CfMago [Cyberlindnera fabianii YJS4271] (CYFA0S05e03620g, this study)
MDIEDTEKTGDLLPDEKEQLEGHSASLSQESDFFLKYYSGHQGQFGHEFLEFEVRLAPDGKSASVSYTNNTNYRR
EAIIRKQFAVSRTVAKELQRFVEEAEIMRETDKNWPTKNREGAQELTISMGGAHVRLATCKIGSLADVKNSADPD
GLRAFYYFVQDVKAFMFSLISLHFKIKPY

```
        1       10        20        30        40        50        60        70        80        90       100       110       120       130
        |--------+---------+---------+---------+---------+---------+---------+---------+---------+---------+---------+---------+---------|
YlMago                                                             MAEEEEYSIATRPGEDVQMEEELEVVEPED-DLIEKPLSLRVDAKEEAFYLQYYSGHTGRFGHEFLEFDFQ
ChMago  MENVGELEGQAVTQTESKSESQSELQTEIQPKPQTEPQVDSQIDLHASQGNPQADSNVDSESASHTEQHTAPDSHLDSYPQTEIDNHREISTNTKRINIDVDRKEDEFSIHYYAGHQGRFGHEFLEFSVE
BaMago                                          MEVDLEGKELGTRAPMSSDLDAGDKNT-----DSGVESGERMLEDKE------ASQIPIDISKADDAFYVRYYSGHQGKYGHEFLEFDLR
GcMago                                                                                                         MTSVINKHQSEPLYIRYYAGHQGRYGHEFLEFDIR
WcMago                                                                                               MSQEENKSIKNAEIDVNDAGQAFYLRYYSGHQGRFGHEFLEFDVE
CfMago                                                                                 MDIEDTEKTGDLLPDEKEQLEGHSASL--SQESDFFLKYYSGHQGQFGHEFLEFEVR

        131     140       150       160       170       180       190       200       210       220       230       240       250 254
        |--------+---------+---------+---------+---------+---------+---------+---------+---------+---------+---------+---------+---|
YlMago  LLDQGRSALLRYANNSNYKNDTLIRREVAVSPVVVKVLKKMIKESEILKENDAKWPPKNRDGKQELEIKFANYHIAFETGRIGSLSDVQRSDDPEGLRTFYYLVQDIKALVFSLVGLHFKIKPI
ChMago  VMGNGSFALLRYANNSNYKNETLIKREVAISPLVIKGFKKIICESEILRESDKDWPERNRDGKQELQVKIGQYSCNLETCRIGSLAEIRDTLDPEGLRVFYYLVQDLKAFLFSLISLHFKIKPI
BaMago  AQANGRFATLRYANNSNYRNDTLIKKETMVSPSVIKEFKKIIADSEILKEDDERWPEKNRDGKQELEVRVGKYHISFETAKIGSLNDVQNSDDPEGLRVFYYLVQDLRAFVFSLISLHFKIKPI
GcMago  VQDDGKSAILRYANNSNYKNDNLIRKEATISPTVVEEIRRIITESEIVHEDDKKWPPKNRDGKQELELRLGSFHISFETAKIGSLSDIQNSDDPEGLRVFYYLVNDLKAFVFSLISLHFKIKPI
WcMago  LNEEKTSALVRYTNNSNYRRDDLIRKEFTISVAVVKQLKQIISDSEILKEDDSKWPEKNADGKQELEIKSGRYHISFETTKIGSLTDVRNSDDPEGLRVFYYFVQDIKALIFSLINLHFKVKPL
CfMago  LAPDGKSASVSYTNNTNYRREAIIRKQFAVSRTVAKELQRFVEEAEIMRETDKNWPTKNREGAQELTISMGGAHVRLATCKIGSLADVKNSADPDGLRAFYYFVQDVKAFMFSLISLHFKIKPY
```

Alignment was performed with the Multalin server (http://multalin.toulouse.inra.fr/multalin/)

⬇ Conserved amino acid residues involved in the interaction with the Y14 protein (V_{100}, V_{104}, D_{176}, L_{180}, S_{183}, L_{184}, L_{187} and H_{188} in the *Y. lipolytica* Mago protein)

⬇ Amino acid residues involved in the interaction with the Upf3 protein (E_{114} and E_{116} in the *Y. lipolytica* Mago protein)

Supplementary Figure S2: Sequences and alignment of Y14 proteins

A. Sequence alignment of Y14 in model genomes

>HsY14 [Homo sapiens] (NP_005096.1)
MADVLDLHEAGGEDFAMDEDGDESIHKLKEKAKKRKGRGFGSEEGSRARMREDYDSVEQDGDEPGPQRSVEGWIL
FVTGVHEEATEEDIHDKFAEYGEIKNIHLNLDRRTGYLKGYTLVEYETYKEAQAAMEGLNGQDLMGQPISVDWCF
VRGPPKGKRRGGRRRSRSPDRRRR

>DmY14 [Drosophila melanogaster] (AAF58987.1)
MADVLDIDNAEEFEVDEDGDQGIVRLKEKAKHRKGRGFGSDSNTREAIHSYERVRNEDDDELEPGPQRSVEGWIL
FVTSIHEEAQEDEIQEKFCDYGEIKNIHLNLDRRTGFSKGYALVEYETHKQALAAKEALNGAEIMGQTIQVDWCF
VKGPKRVKKSEKRRR

>CeY14 [Caenorhabditis elegans] (NP_497891.1)
MSDNEVEMEDVVANAERSRGRGLAQSRNRERITYDVVDEESSATSGAPQRSVEGWIVFVTNIHEEATEDDVHDKF
SEYGKIKNIHLNLDRRTGFLKGYALVEYETQKEANEAIDQSNDTDLLGQNVKVDWCFVKGKKTSGKR

>YlY14 [Yarrowia lipolytica CLIB122] (XP_503462.2)
MGITEDEPTPLRATEGWIIIVDNLHPEISEEDLTDFYSTYGDVQVAHVNLDRRTGYAKGYALLQFATKDDADTAI
AETNGVEFLEHNLEVSFAFHEADTSISQHRIRGSENRSRSPGRRR

>SpY14 [Schizosaccharomyces pombe 972h-] (NP_594439.1)
MRPAKSVEGYIIIVTGVHPEATEEQVEDLFADFGPVKNLHLNLDRRTGYVKGYALIEYATLEQAQKAVDEKNLSL
LDEKLEVDFAFLEPPERAPRPSISTRSRSQSPEVQHRDRDVAMAEP

```
       1       10        20        30        40        50        60        70        80        90        100       110       120      130
       |--------+---------+---------+---------+---------+---------+---------+---------+---------+---------+---------+---------+---------|
HsY14  MADVLDLHEAGGEDFAMDEDGDESIHKLKEKAKKRKGRGFGSEEGSRARMREDYDSVEQDGDE--PGPQRSVEGWILFVTGVHEEATEEDIHDKFAEYGEIKNIHLNLDRRTGYLKGYTLVEYETYKEAQ
DmY14  MADVLDIDNA--EEFEVDEDGDQGIVRLKEKAKHRKGRGFGSDSNTREAIHSYERVRNEDDDELEPGPQRSVEGWILFVTSIHEEAQEDEIQEKFCDYGEIKNIHLNLDRRTGFSKGYALVEYETHKQAL
CeY14                   MSDNEVEMEDVVANAERSRGRGLAQ---SRNRERITYDVVDEESSATSGAPQRSVEGWIVFVTNIHEEATEDDVHDKFSEYGKIKNIHLNLDRRTGFLKGYALVEYETQKEAN
YlY14                                                             MGITEDEPTPLRATEGWIIIVDNLHPEISEEDLTDFYSTYGDVQVAHVNLDRRTGYAKGYALLQFATKDDAD
SpY14                                                                    MRPAKSVEGYIIIVTGVHPEATEEQVEDLFADFGPVKNLHLNLDRRTGYVKGYALIEYATLEQAQ

       131     140       150       160       170       180    188
       |--------+---------+---------+---------+---------+-------|
HsY14  AAMEGLNGQDLMGQPISVDWCFVRGPPKGKRR---GGRRRSRSPDRRRR
DmY14  AAKEALNGAEIMGQTIQVDWCFVKGPKRVKKS---EKRRR
CeY14  EAIDQSNDTDLLGQNVKVDWCFVKGKKTSGKR
YlY14  TAIAETNGVEFLEHNLEVSFAFHEADTSISQHRIRGSENRSRSPGRRR
SpY14  KAVDEKN-LSLLDEKLEVDFAFLEPPERAPRPSI-STRSRSQSPEVQHRDRDVAMAEP
```

Alignment was performed with the Multalin server (http://multalin.toulouse.inra.fr/multalin/)

Amino acids that match a conserved RNA recognition motif found in RNA-binding protein RBM8A, RBM8B and similar proteins (cd12324)

Amino acid residues involved in the interaction of Y14 protein with the Mago protein (I_{74}, F_{76}, H_{103}, N_{105}, K_{114}, Y_{116}, L_{118}, and F_{150} for the *D. melanogaster* Y14 protein, Fribourg *et al.;* 2003)

Amino acid residue involved in the interaction with the human Upf3 protein (Y_{112}, Buchwald *et al.*; 2010)

Amino acid residue involved in Cap binding and translation enhancement (W_{73}, Chuang *et al.*; 2016)

B. Sequence alignment of Y14 in Saccharomycotina

>YlY14 [Yarrowia lipolytica CLIB122] (XP_503462.2)
MGITEDEPTPLRATEGWIIIVDNLHPEISEEDLTDFYSTYGDVQVAHVNLDRRTGYAKGYALLQFATKDDADTAI
AETNGVEFLEHNLEVSFAFHEADTSISQHRIRGSENRSRSPGRRR

>ChY14 [Candida hispaniensis CBS 9996] (OLHI0A05776g)
MEPLQSTQGWILMVNNVHPEMNQEDLMDYFSTFGTVDNIHLNLDRQSGYAKGYALIEYASKEEADTAIEEANGVE
FVDRVLQVDYAFLSVPEDSISRSRARGSFQRRSSRSPGR

>CfY14 [Cyberlindnera fabianii YJS4271] (CYFA0S08e00980g)
MSSRMDVDEKNDSIDNENDNVVPSTKDGLIAAKSIQGYVLIVTNVHEEAAEEDVTDFFSDFGRVRGVHLNLDRQS
GYVKGYALVEFREKSEAEEAVREADGSELLGTIINVDFAFVESPEQTNETKPKQRERSPDRGGRYESRQERDQW

>WcY14 [Wickerhamomyces ciferrii NRRL Y-1031 F-60-10] (XP_011276841.1)
MSDAGEAMDVDQPSTSTIPTTKQGIPIAKSIEGYVIIVQNVHEEATEEDVQEFFEEFGPVKNIHLNLDRQTGYVK
GYALIEYTELQNAIRAVQEGDGEELLGHILSIDYAFVHSDLELKRGKRDKDEEKVRDKSPERS

>GcY14 [Geotrichum candidum CLIB 918] (emb|CDO54368.1)
MSEQNESAPVINEESASAEVAYSAMEVEMTDSNNAAIDSERLPVKSIEGWIIIATNIHEEASEEDVSDFFSEWGQ
VENLHLNLDRRTGYVKGYALVEYPTYEEAERAVREANNVEFLGQALSVAFAFVQGPSESTVIPTARPRRRRNRSN
SRSISPERSPSQSRSPRRRRDSQSQSRSRSRTPDYRSD

>BaY14 [Blastobotrys adeninivorans LS3] (ARAD1D42097g, this study)
MSRQMDVDDGGEAPAIRSVEGWIVLVSNLHEEAAEEDVTDFFSDFGEVQNIHLNLDRRTGYVKGYALIEYATADE
AQRAVTEANGAEMLGKRLEVDFTFVAAPSSYSEKNNRSRSASPTRDARRD

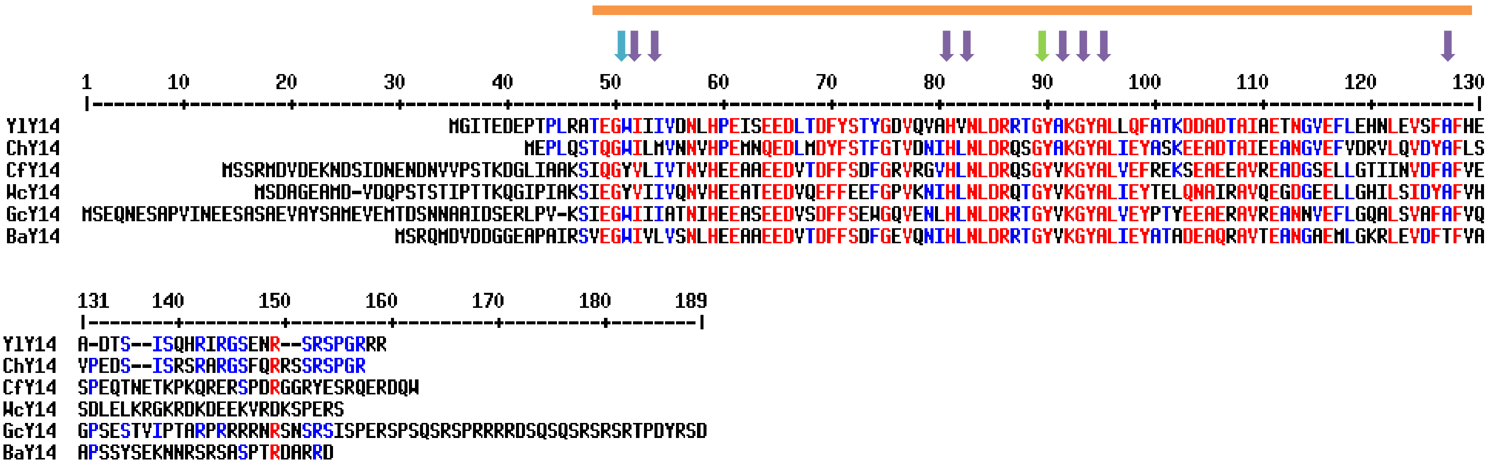

Alignment was performed with the Multalin server (http://multalin.toulouse.inra.fr/multalin/)
Amino acids that match a conserved RNA Binding Domain (cd12324)
Conserved amino acid residues involved in the interaction of the Y14 protein with the Mago protein (I_{18}, I_{20}, H_{47}, N_{49}, K_{58}, Y_{60}, L_{62} and F_{94} for the *Y. lipolytica* Y14 protein)
Conserved amino acid residue involved in the interaction with the Upf3 protein (Y_{56} for the *Y. lipolytica* Y14 protein)
Conserved amino acid residue involved in Cap binding (W_{17} for the *Y. lipolytica* Y14 protein)

Supplementary Figure S3: Sequences and alignment of eIF4A3 proteins

A. Sequence alignment of eIF4A3 in model genomes

```
>Hs_eIF4A3 [Homo sapiens] (NP_055555.1)
MATTATMATSGSARKRLLKEEDMTKVEFETSEEVDVTPTFDTMGLREDLLRGIYAYGFEKPSAIQQRAIKQIIKGRDVIAQSQ
SGTGKTATFSISVLQCLDIQVRETQALILAPTRELAVQIQKGLLALGDYMNVQCHACIGGTNVGEDIRKLDYGQHVVAGTPGR
VFDMIRRRSLRTRAIKMLVLDEADEMLNKGFKEQIYDVYRYLPPATQVVLISATLPHEILEMTNKFMTDPIRILVKRDELTLE
GIKQFFVAVEREEWKFDTLCDLYDTLTITQAVIFCNTKRKVDWLTEKMREANFTVSSMHGDMPQKERESIMKEFRSGASRVLI
STDVWARGLDVPQVSLIINYDLPNNRELYIHRIGRSGRYGRKGVAINFVKNDDIRILRDIEQYYSTQIDEMPMNVADLI

>Dm_eIF4A3 [Drosophila melanogaster](NP_649788.2)
MARKNAQAEDLSNVEFETSEDVEVIPTFNAMNLKEELLRGIYAYGFEKPSAIQQRSITPIVKGRDVIAQAQSGTGKTATFSIS
ILQSLDTTLRETQVLCLSPTRELAVQIQKVILALGDMMNVQCHVCIGGTNLGEDIRKLDYGQHIVSGTPGRVFDMIKRRVLRT
RAIKMLVLDEADEMLNKGFKEQIYDVYRYLPPATQVVLISATLPHEILEMTSKFMTDPIRILVKRDELTLEGIKQFFVAVERE
EWKFDTLCDLYDTLTITQAVIFCNTKRKVDWLTEKMREANFTVSSMHGDMPQKERDEIMKEFRAGQSRVLITTDVWARGIDVQ
QVSLVINYDLPNNRELYIHRIGRSGRFGRKGVAINFVKSDDIRILRDIEQYYSTQIDEMPMNVADLI

>Ce_eIF4A3 [Caenorhabditis elegans] (NP_490761.2)
MAENKKKNDDMATVEFESSEEVSIIPTFDKMGLREDLLRGIYAYGFEKPSAIQQRAIPAILKARDVIAQAQSGTGKTATFSIS
VLQSLDTQVRETQALILSPTRELAVQIQKVVLALGDYMNVQCHACIGGTNLGEDIRKLDYGQHVVSGTPGRVFDMIRRRNLRT
RAIKLLVLDEADEMLNKGFKEQLYDIYRYLPPGAQVVLLSATLPHEILEMTSKFMTDPIRILVKRDELTLEGIKQFFVAVDRE
EWKFDTLIDLYDTLTITQAVLFCNTRRKVDWLTDKMKEANFTVSSMHGDMEQKDRDEVMKEFRAGTTRVLISTDVWARGLDVP
QVSLVINYDLPNNRELYIHRIGRSGRFGRKGVAINFVKQDDVRILRDIEQYYSTQIDEMPMNIADII

>Sp_eIF4A3 [Schizosaccharomyces pombe 972h-] (Q10055.1)
MADEIMENVELTTSEDVNAVSSFEEMNLKEDLLRGIYAYGYETPSAVQSRAIIQICKGRDVIAQAQSGTGKTATFSIGILQSI
DLSVRDTQALILSPTRELAVQIQNVVLALGDHMNVQCHACIGGTSVGNDIKKLDYGQHVVSGTPGRVTDMIRRRNLRTRNVKM
LILDEADELLNQGFKEQIYDIYRYLPPGTQVVVVSATLPQDVLEMTNKFTTNPVRILVKRDELTLEGLKQYFIAVEKEEWKFD
TLCDLYDTLTITQAVIFCNSRRKVDWLTEKMREANFTVTSMHGEMPQKERDAIMQDFRQGNSRVLICTDIWARGIDVQQVSLV
INYDLPANRENYIHRIGRSGRFGRKGVAINFVTNEDVRILRDIEQYYSTVIDEMPMNIGDMV

>Yl_eIF4A3 [Yarrowia lipolytica CLIB122] (XP_504915.1)
MAEFDRDLDDELEFKTSKDVDVTPTFESMDLKDDLLRGIYAYGFEAPSAIQSRAITQIIKGRDTIAQAQSGTGKTATFSISML
EVIDTKHRETQAMVLSPTRELATQIQSVILALGDYMNVQCHACIGGTSLSVDMKKLEAGQQVVSGTPGRCLDMIKKGCLRTKN
LKMLILDEADELLNKGFQEQIYDIYRYLPAATQVVVVSATLPHSVLEMTSKFMTDPVRILVKRDELTLEGLKQYFIAVEQEEW
KFDTLCDLYDTLTITQAVIFCNTKKKVDWLTQQMKDNNFTVCSMHGDMAQKDRDSIMNEFRSGRSRVLISTDVWARGIDVQQV
SLVINYDLPPNRENYIHRIGRSGRFGRKGVAINFATNDDITTLRDIEQYYSTQIDEMPVNVTDMM
```

```
           1       10        20        30        40        50        60        70        80        90        100       110       120       130
           |--------+---------+---------+---------+---------+---------+---------+---------+---------+---------+---------+---------+---------|
Hs_eIF4A3  MATTATMATSGSARKRLLKEEDMTKVEFETSEEVDVTPTFDTMGLREDLLRGIYAYGFEKPSAIQQRAIKQIIKGRDVIAQSQSGTGKTATFSISVLQCLDIQVRETQALILAPTRELAVQIQKGLLALG
Dm_eIF4A3             MARKNA-QAEDLSNVEFETSEDVEVIPTFNAMNLKEELLRGIYAYGFEKPSAIQQRSITPIVKGRDVIAQAQSGTGKTATFSISILQSLDTTLRETQVLCLSPTRELAVQIQKVILALG
Ce_eIF4A3              MAENKKKNDDMATVEFESSEEVSIIPTFDKMGLREDLLRGIYAYGFEKPSAIQQRAIPAILKARDVIAQAQSGTGKTATFSISVLQSLDTQVRETQALILSPTRELAVQIQKVVLALG
Sp_eIF4A3                   MADEIMENVELTTSEDVNAVSSFEEMNLKEDLLRGIYAYGYETPSAVQSRAIIQICKGRDVIAQAQSGTGKTATFSIGILQSIDLSVRDTQALILSPTRELAVQIQNVVLALG
Yl_eIF4A3                MAEFDRDLDDELEFKTSKDVDVTPTFESMDLKDDLLRGIYAYGFEAPSAIQSRAITQIIKGRDTIAQAQSGTGKTATFSISMLEVIDTKHRETQAMVLSPTRELATQIQSVILALG

           131      140       150       160       170       180       190       200       210       220       230       240       250       260
           |--------+---------+---------+---------+---------+---------+---------+---------+---------+---------+---------+---------+---------|
Hs_eIF4A3  DYMNVQCHACIGGTNVGEDIRKLDYGQHVVAGTPGRVFDMIRRRSLRTRAIKMLVLDEADEMLNKGFKEQIYDVYRYLPPATQVVLISATLPHEILEMTNKFMTDPIRILVKRDELTLEGIKQFFVAVER
Dm_eIF4A3  DMMNVQCHVCIGGTNLGEDIRKLDYGQHIVSGTPGRVFDMIKRRVLRTRAIKMLVLDEADEMLNKGFKEQIYDVYRYLPPATQVVLISATLPHEILEMTSKFMTDPIRILVKRDELTLEGIKQFFVAVER
Ce_eIF4A3  DYMNVQCHACIGGTNLGEDIRKLDYGQHVVSGTPGRVFDMIRRRNLRTRAIKLLVLDEADEMLNKGFKEQLYDIYRYLPPGAQVVLLSATLPHEILEMTSKFMTDPIRILVKRDELTLEGIKQFFVAVDR
Sp_eIF4A3  DHMNVQCHACIGGTSVGNDIKKLDYGQHVVSGTPGRVTDMIRRRNLRTRNVKMLILDEADELLNQGFKEQIYDIYRYLPPGTQVVVVSATLPQDVLEMTNKFTTNPVRILVKRDELTLEGLKQYFIAVEK
Yl_eIF4A3  DYMNVQCHACIGGTSLSVDMKKLEAGQQVVSGTPGRCLDMIKKGCLRTKNLKMLILDEADELLNKGFQEQIYDIYRYLPAATQVVVVSATLPHSVLEMTSKFMTDPVRILVKRDELTLEGLKQYFIAVEQ

           261      270       280       290       300       310       320       330       340       350       360       370       380       390
           |--------+---------+---------+---------+---------+---------+---------+---------+---------+---------+---------+---------+---------|
Hs_eIF4A3  EEWKFDTLCDLYDTLTITQAVIFCNTKRKVDWLTEKMREANFTVSSMHGDMPQKERESIMKEFRSGASRVLISTDVWARGLDVPQVSLIINYDLPNNRELYIHRIGRSGRYGRKGVAINFVKNDDIRILR
Dm_eIF4A3  EEWKFDTLCDLYDTLTITQAVIFCNTKRKVDWLTEKMREANFTVSSMHGDMPQKERDEIMKEFRAGQSRVLITTDVWARGIDVQQVSLVINYDLPNNRELYIHRIGRSGRFGRKGVAINFVKSDDIRILR
Ce_eIF4A3  EEWKFDTLIDLYDTLTITQAVLFCNTRRKVDWLTDKMKEANFTVSSMHGDMEQKDRDEVMKEFRAGTTRVLISTDVWARGLDVPQVSLVINYDLPNNRELYIHRIGRSGRFGRKGVAINFVKQDDVRILR
Sp_eIF4A3  EEWKFDTLCDLYDTLTITQAVIFCNSRRKVDWLTEKMREANFTVTSMHGEMPQKERDAIMQDFRQGNSRVLICTDIWARGIDVQQVSLVINYDLPANRENYIHRIGRSGRFGRKGVAINFVTNEDVRILR
Yl_eIF4A3  EEWKFDTLCDLYDTLTITQAVIFCNTKKKVDWLTQQMKDNNFTVCSMHGDMAQKDRDSIMNEFRSGRSRVLISTDVWARGIDVQQVSLVINYDLPPNRENYIHRIGRSGRFGRKGVAINFATNDDITTLR

           391      400        411
           |--------+----------+|
Hs_eIF4A3  DIEQYYSTQIDEMPMNVADLI
Dm_eIF4A3  DIEQYYSTQIDEMPMNVADLI
Ce_eIF4A3  DIEQYYSTQIDEMPMNIADII
Sp_eIF4A3  DIEQYYSTVIDEMPMNIGDMV
Yl_eIF4A3  DIEQYYSTQIDEMPVNVTDMM
```

Alignments were performed with the Multalin server (http://multalin.toulouse.inra.fr/multalin/).

Amino acid residues involved in the interaction with CWC22 (D_{270}, D_{273}, T_{276}, I_{277}, N_{301}, F_{302} and T_{303} in the Human eIF4A3protein, Steckelberg *et al.;* 2012)

B. Sequence alignment of eIF4A3 (Fal1) in Saccharomycotina

```
>Yl_eIF4A3 [Yarrowia lipolytica CLIB122] (XP_504915.1)
MAEFDRDLDDELEFKTSKDVDVTPTFESMDLKDDLLRGIYAYGFEAPSAIQSRAITQIIKGRDTIAQAQSGTGKTATFSISML
EVIDTKHRETQAMVLSPTRELATQIQSVILALGDYMNVQCHACIGGTSLSVDMKKLEAGQQVVSGTPGRCLDMIKKGCLRTKN
LKMLILDEADELLNKGFQEQIYDIYRYLPAATQVVVVSATLPHSVLEMTSKFMTDPVRILVKRDELTLEGLKQYFIAVEQEEW
KFDTLCDLYDTLTITQAVIFCNTKKKVDWLTQQMKDNNFTVCSMHGDMAQKDRDSIMNEFRSGRSRVLISTDVWARGIDVQQV
SLVINYDLPPNRENYIHRIGRSGRFGRKGVAINFATNDDITTLRDIEQYYSTQIDEMPVNVTDMM

>Cf_eIF4A3 [Cyberlindnera fabianii YJS4271] (CYFA0S08e03972g)
MSFDRQQDASLEFKTSKNVDIVASFEEMNLKEDLLRGIYSYGFEAPSAIQSRAILQIISGRDTIAQAQSGTGKTATFSIGMLE
VLDTKNRETQAIVLSPTRELAVQIQNVIKSLGSYMNVQCHACIGGTSVGQDIKQLAKGQHIVSGTPGRVLDMIKRRNLNTRNV
KMMILDEADELLSKGFKEQIYDIYRQLPASTQVVVVSATLPRDVLDMTNKFTTDPVKILVKRDELTLEGIKQYFIDVEKEDWK
FDTLCDLYDSLTIMQSVIFCNTKKKVDWLTEKMRGANFTVVAMHGDMKQDERDQIMNDFRLGNSRVLISTDVWARGIDVQQVS
LVVNYDLPYDRENYIHRIGRSGRFGRKGVAINFCTNEDIQALHDIEQYYSIQIDEMPANIESML

>Kc_eIF4A3 [Kuraishia capsulata CBS 1993] (T00003499001)
MEFDKELDSTLRFKSSKNIPVVASFEDMKLKDDLLRGIYDYGFEEPSAIQSRAIKPIIAGRDTIAQAQSGTGKTATFGIGILQ
VCDTKSRETQAIVLSPTRELALQIQKVLKSLGSFMNLHCHACIGGTSVGEDMKQFGKGQQVVSGTPGRVVDMIKRRTLNTRNI
KMLVLDEADELLNKGFQEQIYEVYRYLPPSTQVVVVSATLPNEVLSMTKKFMNDPVKILVKRDDITLEGISQFYIDVDKEDWK
FDTLCDLYDSMTISQAVIFCNTKKKVDWLCEAMRKANFTVVAMHGDMKQEERDKIMQEFRSSQSRVLISTDVWARGIDVQQVS
LVINYDLPYDREMYVHRIGRAGRFGRKGVSINFVTKEDKPELHALERYYSVKISEMPADINNLL

>Ca_eIF4A3 [Candida albicans SC5314] (C1_05640C_A)
MDDFDRDLDNELEFSHKSTKGIKVHRTFESMNLKPDLLKGIYAYGFETPSAIQSRAIMQIISGRDTIAQAQSGTGKTATFSIG
MLEVIDTKSKECQALILSPTRELAIQIQNVVKHLGDYMNIHTHACIGGKNVGEDVKKLQQGQQIVSGTPGRVIDVIKRRNLQT
RNIKVLILDEADELFTKGFKEQIYEIYKHLPPSVQVVVVSATLPSEVLEMTSKFTTDPVKILVKRDEISLLGIKQYYVQCESE
DWKFDTLCDLYDNLTITQAVIFCNTKLKVNWLADQMKKQNFTVVAMHGDMKQDERDSIMNDFRRGNSRVLISTDVWARGIDVQ
QVSLVINYDLPTDKENYIHRIGRSGRFGRKGTAINLITKDDVVTLKEFEKYYSTKIKEMPMNINDIM

>Dh_eIF4A3 [Debaryomyces hansenii CBS 767] (DEHA2D04048g)
MDDFDRDVDNELEFSAKSTKNIKVHATFESMNLKTDLLKGIYGYGFEAPSAIQSSAIMQIISGKDTIAQAQSGTGKTATFSIG
MLEVIDTKSKDCQALILSPTRELAQQIQSVVKHLGDYMNVHTHACIGGTHVGEDIKKLQQGQQIVSGTPGRVVDMIKRSNLAT
SNIKMMILDEADELMTKGFKEQIYEIYRYLPPGVQVVVVSATLSREVLEVTGKFTTDPVKILVKRDDITLEGIKQYHIQCEKE
DWKFDTLCDLYDSLTITQAVIFCNTKVKVNWLTDQMKKANFTVVAMHGDMKQDERDSIMNDFRTGNSSVLISTDVWARGIDVQ
QVSLVINYDLPTDKENYVHRIGRSGRFGRKGVAINLVTKEDVDELSDIESFYSIRIKEMPVNVNDIM

>Ba_eIF4A3 [Blastobotrys adeninivorans LS3] (ARAD1D20790g)
MEDELEFRSSEGVGVYGSFEAMNLHEDLLRGIYAYGFEAPSAIQSRAITQIMSGRDVIAQAQSGTGKTATFSIGMLDVLDIRS
RSTQALVLSPTRELAVQIQKVVLALGDYMNAQCHACIGGKSVGEDIRALERNPHVVSGTPGRVADMIRRSYLSTRSVKMLILD
EADTLLDMGFQSQIYEIYRYLPPATQVVLVSATLPPDVLSLTSKFMNDPVRILVKRDELTLEGLKQYFIAVEKEDWKFDTLCD
LYDTLTITQAVIFCNTKRKVDWLTDKMREANFTVSSMHGDMDQKERDQVMNEFRGGKSRVLISTDVWARGIDVQQVSLVINYD
LPSHRENYLHRIGRSGRFGRKGVAINFVTDDELKILHDIEQFYSTQIEEMPININDML

>Sc_eIF4A3 [Saccharomyces cerevisiae S288c] (NP_010304.3)
MSFDREEDQKLKFKTSKKLKVSSTFESMNLKDDLLRGIYSYGFEAPSSIQSRAITQIISGKDVIAQAQSGTGKTATFTIGLLQ
AIDLRKKDLQALILSPTRELASQIGQVVKNLGDYMNVNAFAITGGKTLKDDLKKMQKHGCQAVSGTPGRVLDMIKKQMLQTRN
VQMLVLDEADELLSETLGFKQQIYDIFAKLPKNCQVVVVSATMNKDILEVTRKFMNDPVKILVKRDEISLEGIKQYVVNVDKE
EWKFDTLCDIYDSLTITQCVIFCNTKKKVDWLSQRLIQSNFAVVSMHGDMKQEERDKVMNDFRTGHSRVLISTDVWARGIDVQ
QVSLVINYDLPEIIENYIHRIGRSGRFGRKGVAINFITKADLAKLREIEKFYSIKINPMPANFAELS

>Lk_eIF4A3 [Lachancea kluyveri CBS 3082] (SAKL0D03960g)
MSFDRENDSKLKFKTSTKLKVSPTFESMKLKEDLLRGIYSYGFEAPSAIQSRAITQIISGRDVIAQAQSGTGKTATFTIGMLQ
AIDVKSKDLQSLVLSPTRELATQISQVVSNLGDYMSISAHACTGGKTLQADIKKLSKSCQVVSGTPGRVLDMIKRRLLNTRNV
KMLVLDEADELLSETLGFKQQIYDIFTKLPTSVQVVVVSATMNKDILEITKKFMSDPVKILVKRDEISLEGIKQYHVNVDKEE
WKFDTLCDLYDSLTITQCVIFCNTKKKVDWLSHKLAQANFAVSAMHGDMKQEERDKVMNDFRNGTSRVLISTDVWARGIDVQQ
VSLVINYDLPEILESYIHRIGRSGRFGRKGVAINFITKEDVQKLKEIEKCYSIRIKPMPANIGDLS

>Kl_eIF4A3 [Kluyveromyces lactis CBS 2359] (KLLA0A10659g)
MSFNRDDDSKLKFKTSKKLKVSATFESMNLKPDLLRGIYFYGFEYPSSIQSRAISQIISGKDVIAQAQSGTGKTATFTIGLLQ
AIDSKSKELQALVLSPTRELASQSESVISNLGDYLNVTAHACTGGKALQQDIKKVSKNCQVVSGTPGRVLDMIKRQVLNVRNC
KILVLDEADELLGETLGFKQQIYDIFTKLPPTIQVVVVSATMSKDILEITKKFMSDPVKILVKRDEISLDVIKQYYVDVEKEE
WKFDTLCDLYDSLTITQCVIFCNTRKKVDWLSRKLTQTNFSVSSMHGDMKQEERDQVMNDFRSGKARVLISTDVWARGIDVQQ
ISLVINYDIPDNLENYIHRIGRSGRFGRKGVAINFITKEERPKLKEIESHYRIKIKPTPANLEELS
```

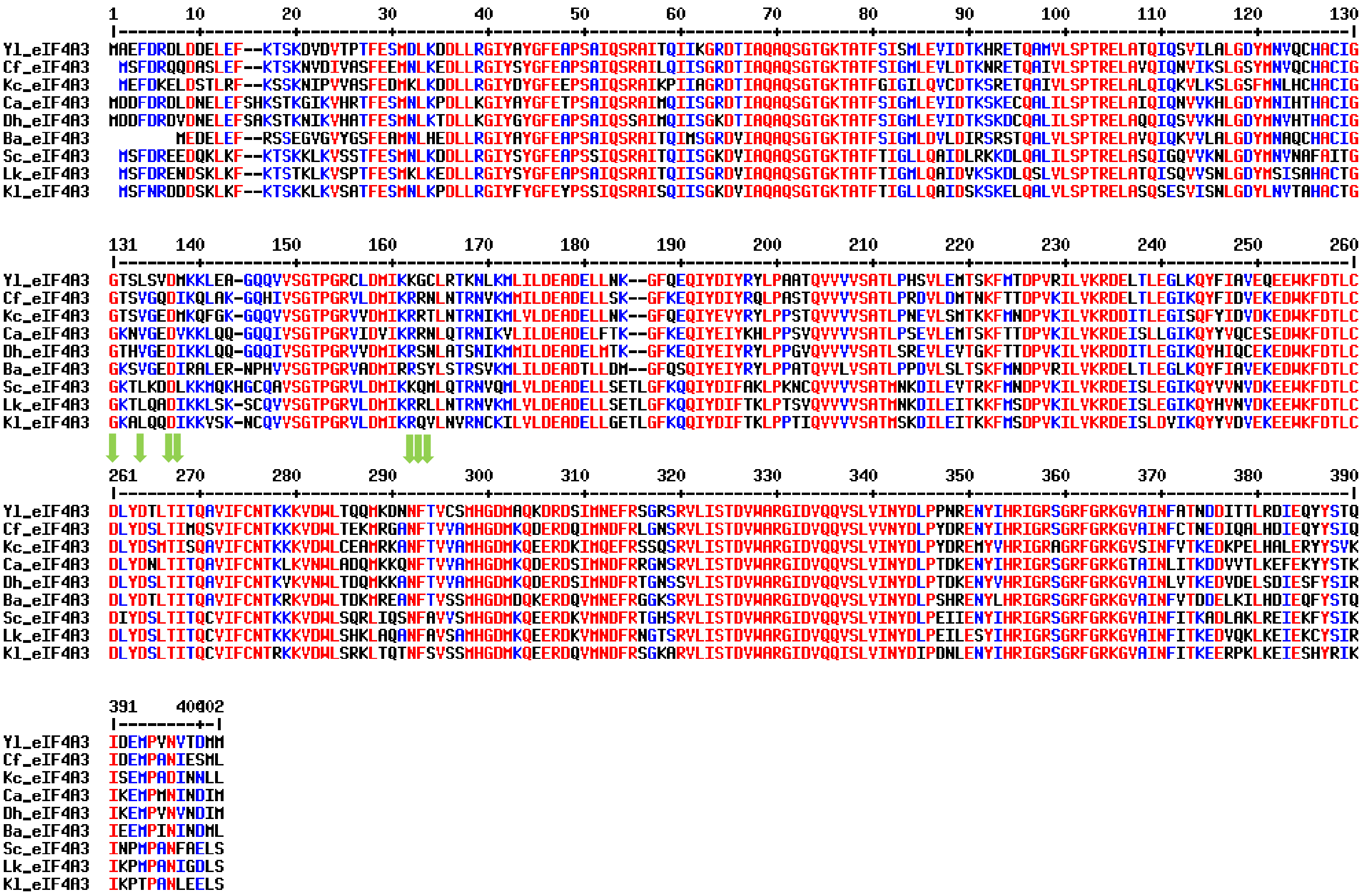

Alignments were performed with the Multalin server (http://multalin.toulouse.inra.fr/multalin/). Sequences for *Cyberlindnera fabianii* (Cf), *Kuraishia capsulata* (Kc), *Candida albicans* (Ca), *Debaryomyces hansenii* (Dh), *Blastobotrys (Arxula) adeninivorans* (Ba), *Saccharomyces cerevisiae* (Sc), *Lachancea kluyveri* (Lk) and *Kluyveromyces lactis* (Kl) were extracted from GRYC (http://gryc.inra.fr/)

Amino acid residues involved in the interaction with CWC22 (D_{256}, D_{259}, T_{262}, I_{263}, N_{287}, F_{288} and T_{289} in the *Y. lipolytica* eIF4A3 protein).

Supplementary Figure S4: Sequences and alignments of Cwc22 proteins

A. Sequence alignment of Cwc22 in model genomes

```
>HsCwc22  [Homo sapiens] (NP_065994.1)
MKSSVAQIKPSSGHDRRENLNSYQRNSSPEDRYEEQERSPRDRDYFDYSRSDYEHSRRGRSYDSSMESRNRDREKRRERERDTDRKRSRKSPSPGRRNPETSVTQSSSA
QDEPATKKKKDELDPLLTRTGGAYIPPAKLRMMQEQITDKNSLAYQRMSWEALKKSINGLINKVNISNISIIIQELLQENIVRGRGLLSRSVLQAQSASPIFTHVYAAL
VAIINSKFPQIGELILKRLILNFRKGYRRNDKQLCLTASKFVAHLINQNVAHEVLCLEMLTLLLERPTDDSVEVAIGFLKECGLKLTQVSPRGINAIFERLRNILHESE
IDKRVQYMIEVMFAVRKDGFKDHPIILEGLDLVEEDDQFTHMLPLEDDYNPEDVLNVFKMDPNFMENEEKYKAIKKEILDEGDTDSNTDQDAGSSEEDEEEEEEEGEED
EEGQKVTIHDKTEINLVSFRRTIYLAIQSSLDFEECAHKLLKMEFPESQTKELCNMILDCCAQQRTYEKFFGLLAGRFCMLKKEYMESFEGIFKEQYDTIHRLETNKLR
NVAKMFAHLLYTDSLPWSVLECIKLSEETTTSSSRIFVKIFFQELCEYMGLPKLNARLKDETLQPFFEGLLPRDNPRNTRFAINFFTSIGLGGLTDELREHLKNTPKVI
VAQKPDVEQNKSSPSSSSSASSSSESDSSDSDSDSSDSSSESSSEESDSSSISSHSSASANDVRKKGHGKTRSKEVDKLIRNQQTNDRKQKERRQEHGHQETRTERERR
SEKHRDQNSSGSNWRDPITKYTSDKDVPSERNNYSRVANDRDQEMHIDLENKHGDPKKKRGERRNSFSENEKHTHRIKDSENFRRKDRSKSKEMNRKHSGSRSDEDRYQ
NGAERRWEKSSRYSEQSRESKKNQDRRREKSPAKQK


>DmCwc22 [Drosophila melanogaster] (NP_609877.2)
MGESDAESDSSSNSSSSDTSSGSDSDARSESSSSESSGREEEEAKQEESAKDAKKTDDTDRGEKRAKERDAGQDEQPTEQKKTPAAEPRSERQHISHSAGVEKQQEEAV
AAAEAESEKLNEAKKVETPVQRKEEAEASSVTKELNSPKAQEENAARELEERRKDEEQPVTTNGSSKESPVEAAAETVKPPTADHIEEGEITDKDEDDLPTKEEKKAVA
SKSPPKETQRKQSRSPDGKRRRPRSSSRSPSPSSRRRRRSRSKGSRTRSRSKSPIRRRSNSLERRRVERQRRHEERDKRDEERAKEREKRHQKGEPTSSRRRDDSREKK
RSPERKRDRSSSTPKSKSSKTPRHTETTETNADNETVTEPAAKITERQRKTVDVLTSRTGGAYIPPAKLRMMQSQITDKSSAAYQRIAWEALKKSIHGYINKVNVTNIA
IITRELLRENIVRGRGLLSRSIIQAQAASPTFTHVYAALVSIINSKFPNIGELLLKRLVIQFRRAFRRNDKMVCMSATRFIGHLVNQRVAHEILALEILTLLVETPTDD
SVEVAIAFLKECGMKLTEVSSKGIGAIFEMLRNILHEGKLDKRVQYMIEVLFQIRKDGFKDHQAVVPELELVEEDDQFTHLMMLDEATETEDILNVFKFDDNYAENEDK
YKGLSREILGSDDGSSSGSGSGSDSDSDSDGESGSDAEKKAEAGDIIDSTETNLIALRRTIYLTINSSLDYEECAHKLMKMQLKPGQEIELCHMFLDCCAEQRTYEKFY
GLLAQRFCNINKIYIPPFEEIFKDTYQTTHRLDTNRLRNVSKFFAHLLFTDAISWDVLECIQLNEDDTTSSSRIFIKILFQELAEYMGLGKLNAKLKDDVLVESIAGLF
PKDNPRNTRFSINFFTSIGLGGLTDDLRRFLKNAPKSVPAINAEILANAGGNPFRDGSAPAGNTKVAPSSSSSSSSSSDTDSEDSSEEDSSSDSSSESSSSDSSSEPKK
KRKRKDKDKKKSKKATKEKSKKTKNKKKKKKAEKEQEKEKEKQRKSKKEKEKDKKRKKEEKKAAKKKSKHRRKSQESSDSSGSEDSDKSTSESSDSSNSSSDESDAEPQ
AKIKRQEHVEKNKFRGRTQDSDEFNLEGPGSKNRFQPNGNGQRRRDNSTGRERNRENSSYDRERNRGNSSYDRERKRGNSSYDRERNRESSYDKERKNRNAVAHDRQRK
RDRSRSYERPTIRENSAPREKRMESSRSEKDSRRGDRSSRNERSDRGERSDRGERSDRGERSDRGERSDRGERSDRGERSDREKERSRAKERERDRDRDLKGQRERKRE
RDDGSRDRSRRERSSRRSKGRS

>CeCwc22 [Caenorhabditis elegans] (NP_496363.1)
MSSSRSQSPETPKAASEEREEEEKESSEQPDTPKTSSEKSASRSQSPRESREVSQETETSENQEKIKEKDDGDDQPGTPNSYRSRETSPAPKRSKETRESESPEKSPVR
SRSPRRSSARSPSRSPRRRRERSSERKQSEEPAPLPEKKKKEPLDILRTRTGGAYIPPAKLRLMQQQISDKQSEQYQRMNWERMKKKIHGLVNRVNAKNLVQIVRELLQ
ENVIRSKGLLCRDIIQAQAFSPGFSNVYAALAAVINSKFPHVGELLLRRLIVQFKRSFRRNDRGVTVNVIKFIAHLINQQVAHEVLALEIMILMLEEPTDDSVEVAIAF
LKECGAKLLEIAPAALNSVYDRLRAILMETERSENALDRRIQYMIETAMQIRKDKFAAYPAVIEDLDLIEEEDQIIHTLNLEDAVDPENGLNVFKLDPEFEKNEEVYEE
IRKEIIGNADISDEDGGDELDDEEEGSDVEEAPKKTTEIIDNTDQNLTAFRREVYLTMQSSLDYQEAAHKLLKMKIPDSMQNELCAMLVDCCAQQRTYERFYGMLIERF
CRLRLEYQQYFEKLCQDTYSTIHRIDITKLRNLARLIAHLLSTDAIDWKILADMKMTEEDTTSSGRIYIKYIFNELVEAMGMVKLHSRVTDPTLAHCFVGLFPRTNPNS
ARFSINFFTMIGLGGLTLELREWLAKGLKKKKGMLDQLKAESSSDSSSSSDSSDSSDSSDSDDSSDSSSDSSSSSESEPEPPKKKKKKNSEESSKKKEKENIGRRDRGD
KRAERHRDQSVENKDKDRRRRQDSDENRRPERGDDRKDRSKDRRRQDSDDEDRKGRERREDSGERRRGDRDRRDRNKDQEDHREDRRDRSKDREDRRDRRRHDSDDDRK
TRRDRSEERGGRRREVESDDRRRRR

>SpCwc22  [Schizosaccharomyces pombe 972h-] (NP_596256.4)
MEKEDKSFGIGMLDYNRENPESSGHSRVAVIRKQKTEQENNNLSWEDRHYIPDLHKSNKIKTPTSLADEKKSHNELDPKAQIKKLMETRSGGTYIPPAKLKALQAQLTD
VNTPEYQRMQWEALKKSINGLINKVNKSNIRDIIPELFQENIIRGRALYCRSIMKAQAASLPFTPIYAAMTAVINTKFPQIGELLLTRLIVQFRKSFQRNDKSMCISSS
SFIAHLINQKIAHEIVGLQILAVLLERPTNDSIEIAVMLLREIGAYLAEVSTRAYNGVFERFRTILHEGQLERRTQFIIEVLFQTRKDKFKNNPTIPQELDLVEEEDQI
THYISLDDNLDVQESLGIFHYDPDYEENEKKYDAIKHEILGEEDDDENEEDEEDSEETSESEEDESVNDEKPQVIDQTNASLVNLRKSIYLTIMSSVDFEECCHKLLKI
DLPEGQEIELCNMVIECNSQERTYAKFYGLIGERFCKLSRTWRSTYEQCFKNYYETIHRYETNRLRNIALFFANLLSTDSIGWEVYDCVRLTEDDTTASSRIFLKIMFQ
EIVEALGLKSLVERLHDPNLVPYLHGLFPVDEARNVRFSINYFTSIGLGALTEEMREYLLTMPKSEPKEQDSEGYSSGSETGSTYSSSYSSTYSRGRSYSRSTRSYSKS
RSYSRSRSVTPINNINHKKYIRKDRELSPRGRERSSNRNSYSDLSRSSSLSRGRSRSYTPEGRLIESEDKGYRSRSSSPASRKYRSRQRYRRSYAGSTSRGRSFSRSPS
YRRRLSMSCSVSYSRSPSPARPISRSPARNKRSYDSLSYNRQYSPKVSRKRSNESRSPSPYTLRKQKTYHLNKRNEDFVVKMDKKGSESPVILAPWLREVDDGELYKDR
NSESDSVRKQPRAD

>YlCwc22 [Yarrowia lipolytica CLIB122] (XP_503087.1)
MSDKERERERSQSRERSRTRERSRTRDSKRDHSDSRSLRRRSYRGDRSRSPSRSRSRSYSPSRSPRRKRRRHRTSRRSRRSRREGDGRRKDGPRGHDDRSPLAPREVED
VTVKREDSTNDKESTHDKESTNDKERNVSMEDRKDTSEDVVEDVEEGDPSEEALEKKVKNPEDDLEAAAEELKKLMELKSGGRYVPPAKIRALQKLLVQDKTSKEFQKI
QFDNLKKAINSLVNKVSAQNIRDIAGEIFTHNLIRGRGLFCRSVMTAQSLALPYTPVYACLTAIVNSKLPQVGELLVRRLILQFRRGYKRNQKDVCLSSVTFLAHLCNY
HVAHEVLVLQLLHLLLETPTDHSVEVAVAFIKESGAFLAEVSPAANNGVFERLRAVLHDGELEKRTQYMIETLFQIRKDGYENYPVVQEELDLVDEEDYVTHMTGLDDK
FTDDKLLNYFVMDPDYEANEEKYDLLKKEILGDSDDEEEDDSEAEEEADDEEEEEGDEEEEAQASTSAVRDLTGTELATLRKKIYLTVMSTMSIDEIVHKLVKLSRTVI
EIPEGLPEDQALILRLKRTQEVTNMLVECCAQEKIYNKIYGGTGERLLRLSREWRTNFENTFGFFYSVIHRYEPNQIRNIATFFGYLLASDSLSWKVLEAVSLTEEDSN
PSNRIFLKIMFTEMRQELGMDLLKERLSKPFVQQYIAGMFPKTNASHVRFAINYFTAINLGPLTDGMREILPGLVEEEERLKKEEEEQKREYDSYDSYDSYDSYDSYDS
YDSYDSYDSYDSYDSYDSYDSYDSYDSVSEDDRGRRRRRGVGSRRRSLTRSPGSRSRPRSRPRSRSRSRSLSRDRTGSPPRGRRRSRSYSRSPSRSYSRSRSYSRS
PSPRPKSKGAKGRSSYRAASYSRSRSPVRRVGYSRSRSPSVSGGSSRSPSRSPCRSPNKGRAPTPEKGLSPPRKRVRASDLF
```

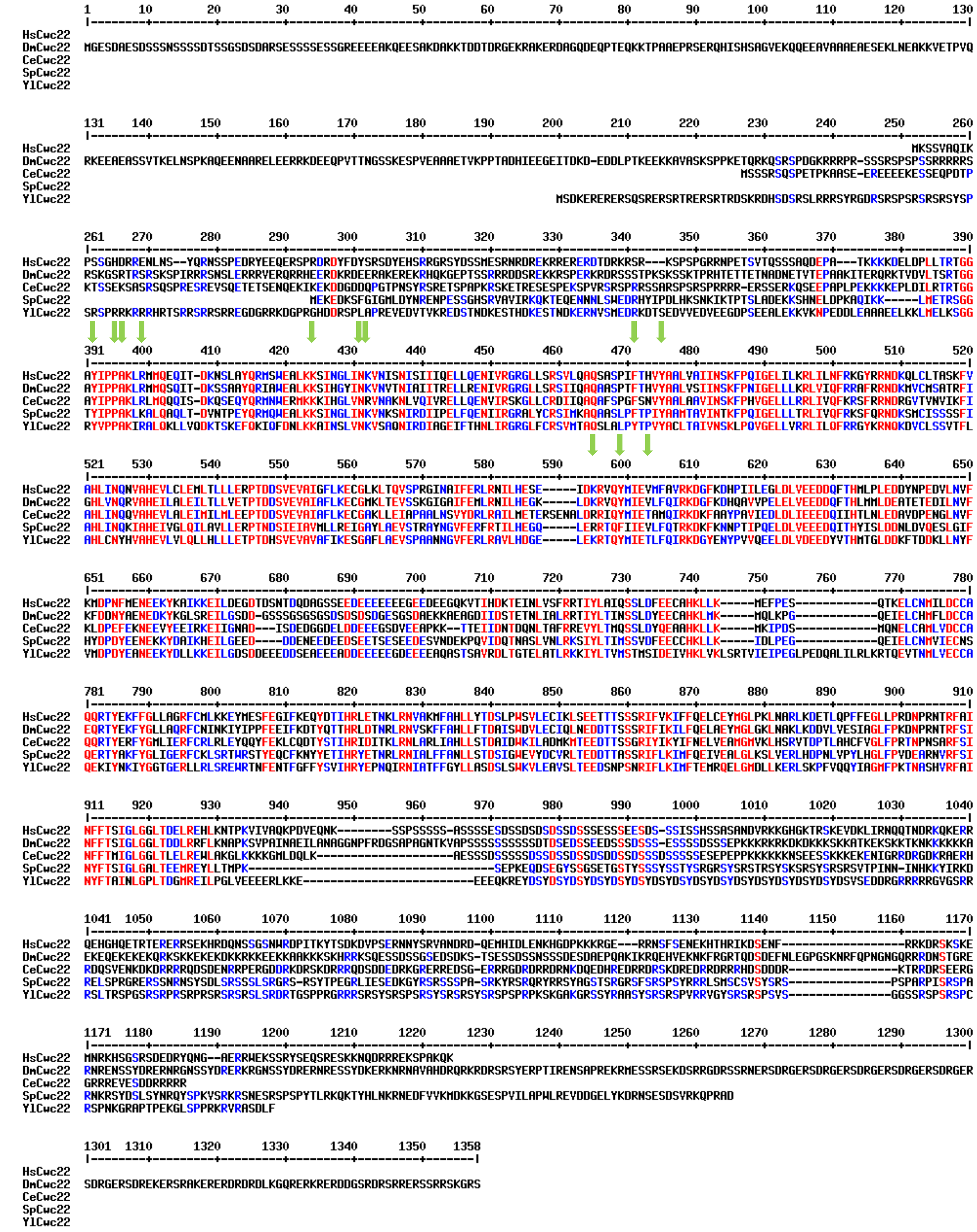

Alignment was performed with the Multalin server (http://multalin.toulouse.inra.fr/multalin/)

Amino acid residues involved in the interaction with eIF4A3 in Human (Y_{133}, P_{136}, A_{137}, R_{140}, K_{164}, N_{171}, K_{172}, F_{211}, Y_{215}, K_{330}, Y_{334} and V_{338} ; Steckelberg *et al.*; 2012, Buchwald *et al.*; 2013)

B. Sequence alignment of Cwc22 in Saccharomycotina

>YlCwc22 [Yarrowia lipolytica CLIB122] (YALI0D20790g)
MSDKERERERSQSRERSRTRERSRTRDSKRDHSDSRSLRRRSYRGDRSRSPSRSRSRSYSPSRSPRRKRRRHRTSRRSRRSRREGDGRRKDGPRGHDDRSPLAPREVED
VTVKREDSTNDKESTHDKESTNDKERNVSMEDRKDTSEDVVEDVEEGDPSEEALEKKVKNPEDDLEAAAEELKKLMELKSGGRYVPPAKIRALQKLLVQDKTSKEFQKI
QFDNLKKAINSLVNKVSAQNIRDIAGEIFTHNLIRGRGLFCRSVMTAQSLALPYTPVYACLTAIVNSKLPQVGELLVRRLILQFRRGYKRNQKDVCLSSVTFLAHLCNY
HVAHEVLVLQLLHLLLETPTDHSVEVAVAFIKESGAFLAEVSPAANNGVFERLRAVLHDGELEKRTQYMIETLFQIRKDGYENYPVVQEELDLVDEEDYVTHMTGLDDK
FTDDKLLNYFVMDPDYEANEEKYDLLKKEILGDSDDEEEDDSEAEEEADDEEEEEGDEEEEAQASTSAVRDLTGTELATLRKKIYLTVMSTMSIDEIVHKLVKLSRTVI
EIPEGLPEDQALILRLKRTQEVTNMLVECCAQEKIYNKIYGGTGERLLRLSREWRTNFENTFGFFYSVIHRYEPNQIRNIATFFGYLLASDSLSWKVLEAVSLTEEDSN
PSNRIFLKIMFTEMRQELGMDLLKERLSKPFVQQYIAGMFPKTNASHVRFAINYFTAINLGPLTDGMREILPGLVEEEERLKKEEEEQKREYDSYDSYDSYDSYDSYDS
YDSYDSYDSYDSYDSYDSYDSYDSYDSYDSVSEDDRGRRRRRGVGSRRRSLTRSPGSRSRPRSRPRSRSRSRSLSRDRTGSPPRGRRRSRSYSRSPSRSYSRSRSYSRS
PSPRPKSKGAKGRSSYRAASYSRSRSPVRRVGYSRSRSPSVSGGSSRSPSRSPCRSPNKGRAPTPEKGLSPPRKRVRASDLF

>BaCwc22 [Blastobotrys adeninivorans LS3] (ARAD1B08140g)
MVDEGNAVDPEAEYKRLLELRKEGKYVPPAKLRALEQQLASSQSEKDRQRIEWDNLKKSINRLVNQVSPENIRSVVVELFKHNIIRGRGLFARSVIRAQGQLLSYTNVY
AALVAVINSKLPQVGELVVNRLVIQFRRAFKRNNKATCLATSQFLAHLCNQQVAHEIISLQLLHLLLERPTDDSTEIAVGFVKEVGSYLSEVSPTALRGVFDRFRVILQ
EGTLEKRTQYMIEVLFQLRRKGLDQTPPVREDLDLVEEEEQITHMISLDDDDLKSQDALNVFKYDDHYEETEAKYEEIKKDILGDSDEEESESESESSSSSDDEEEPEPEP
QGESQRSGATTKITDMTNADFVNLRKTIYLTIMSAMSVPEMTHKLLKLAVPDEMQIEKVNMLIEACAQEKTYNKSYAGVANSLCRVSWHWQNRFKEAFKRYYETIHRYD
TNKLRNIATMFGSLLAGDGLSWEVFETVHMNEQDTNPSTRIFLMILLEEMRRELGIKELAQRFKDPIYQEALQDMFPKKGAEETRFAINYFTAIKLGVLTEDMREYLKN
LPPEPESDSSSSRDSRGRSYSRSSRDSRGRSYSRSPDSRGRSYSRSSRDSRARSYSRSRSYSRSSRDSRGRSYSRSRSYSRSSRDSRGRSYSRSRSYSRSSRDSRGRSY
SRDSRGRSYSRSRSYSTDSRGRSYSRGRSYSRSSRSPSTSKPRPPRPSARSPQAPRSPGRPTEANSNPDPRKPVPDNPANSTIEKGLRAAPHDERREKRPLPRDAPEEG
SRHNNGEFARKRRARASDFLD

>CfCwc22 [Cyberlindnera fabianii YJS4271] (CYFA0S09e04390g)
MSEHRGSLTSAEEEYKRLQELRSGGTYVPPAKLRALLETINFEKGSQEMQRMEWDNLKKKINGLVNKANKSNLKSIVVELFKLNLNRGLGLLVRTLMKAQSQALGFTHV
YAALVAILNSKLPQVGELLISRLVLQFKKAFKRNNKVLCLSSTTFLAHLTNNQVCHEIIVLELLHLLLNNATDDSVDICCGIVKVSGQYLSEVSANAISQVFEKLRQIL
QEGRVEKRTQYMIEVLFQIRRDGYKDFPVVDEELDLVEEEDQITHKVRLDDKLKAQDGLNVFKFDPDYDLHEAEYATVRNEILGREAEDVEEYGDVEVEEDQVLEEQKE
AEEQNADGGKTPSTLEIKDMTDTNLTNFRKTIYLILKGSMSPDEAVHKILKLGIRHEDEDKVVDMLVKACSQENTYSKYYGVTGEKLCGQTKSWASAFAFVFRENYETI
YRLESNPLRNVSKFWGHMLSSDALGWEALDIITLTETDTSSAGRIFIKFVFQELVEELGIPELNERLQEDYMQQHLKGLFPTENSEHLQFSINYFTAIGLGILTERMRE
SLERLKQKRLEEDRGRSRSRSRSGSFSRTTSRSRSSSRGRSYSRTPSRSRSYSRSRSYSDSRSRSGSYSRSGSYSRSRSYSRSRSYSRSRSYSRSRTYSRTPSKSPRRS
RYSRTPSPRRNSRSPSYSRSPPRRNHSRSPSPPRDVPRPRGPLPDQNSAYRSSGFTHGGGRAGGYNQGSGKFRR

>KcCwc22 [Kuraishia capsulata CBS 1993] (T00004580001)
MSEKHTDAQKEYQRLVALKSSGTYVPPAKLRTVLAQINVSKDSEEYQKLQWEALRKSISGLVNKVSASNIKTIVVDLFKLNLQRGRAIFVRAILRAQIASPTMTRVYAA
LVAIVNSKIPEIGELMLARLILQFRSGYKRKDKQQVLATTTLIAHLVNYRVSHEIVALQVLHFLLESSSDDAVEISVNLMTEVGATLTETSSRACGVILERFRVVLQEG
MVNKRVQHLIQVLFQERREEFKDHPSVVKELDLVEEEDQTTHLVGLDDELDANAGLDEYKFDADYEENEKKYNRLKHDILGSDDEDDQSGSESGSESDSESGSASESES
ESESDEDKDDKVKMEITDLTAADLANFQKMVYLTIMSSMSHEEVVHKLLKLKPLDENRKEYMMVDMVAKCCSQEKTYSKYYGLIGENLARLGKRWDLAFRECFQEYYGN
AHRLENNPLRNVSMFWGHMFASDTVGWEALACVKITDEDTTSAGRIFLKFMFEKMRQELGLEKMLVRIKEPYIRPFLKGCFYPEKEDDLRYSINFFTAIKLGALTEDMR
ETLNNLPEAIEETESRGRSRSRSGSFTGSNSSYSHSSSRSGSYSRSRSASYSRSPSPRPKKSRSGSRSGSRSTSRSRSYSRSRSPTSRRPELPPHLRDQKDAFEEYSRN
RERSPPRRNFSKSGRGRGNFNPRARR

>DhCwc22 [Debaryomyces hansenii CBS 767] (DEHA2C12870g)
MSNNSAEYKELLDLKSSGKYVPPAKLKALQTKINNSSESTTEEYQVLQWEQLKRAINRQVNKCNVSNISEIVVELFKLNLQRGKGLLIRSIMKAQLTDLIFTPIYASLI
AVLNSKIPEVGELILNSLLLQFRKNYIKNKKNCISSAIFIVHLINQRVCSEILILQILQLLLENPTNDSIEICVEIMNQVGKYLQENSVAANNMIFNRLRSILHENEDI
NDRSQFLIENLFKTRKNGYSEYPIIRKELDLVDLDDQETHLLELDAKVKSNDQLNIFQFDEQYDENEKLYDNVRKDILGDSDEEDDESEAEESEEDNKEILEIKDMTES
NLLNYQKTVYLTVMSSMSSDEAVHKLIKLNFKKSNEEKYKNNEILVDMIIKCCSQEKTYSKYYGVIGEKLCSMNKLWHTIFIDTFKKYYSTIHQFETNSLRNIGKFFGH
LFALDKLAIERSWNVIRLTEEETNSASRIFIKFIFQEMIEEIGIKGLQERLDDDLIRQETNGLFPRQGVTYRNAEDIRFSINFFTAIGLGILTEEMRDVLKNLPPEERG
RSRSVSGSSRSGSSSYSRSRSYSRSSGSYSRSRSRSRSRSRSSSRTGSRGSTMSRDRSKSFSRSRSTSRSRGESFSRSPSSLPTGSKRFKPN

>CaCwc22 [Candida albicans SC5314] (C2_10100W_A)
MENSTTTTTTTTVTQPDESYQRTKWIETKKNIKQLLQQLTPSNIKQTVLQLFQINLLRYQGLFIREIMKQQIRITTNAELYGSLISIINSKIPEIGELLINRLVLQFKK
NYLQNNKNLINSSIIFICQLINQQVLNEILILQILQMLLESNVPNNNNNNNNNNIELAIMVLKQTGLYLFKHSNTALIMILNRLKDILQDGANANANGGNGGVGLSSWN
RKSIEYILKLARNDFKNISIIKNGLDLVETEDKETHVITLEDKLYSRDHLNVFSVDEEYLDHENEYIELKKEILGETDHEDENENEIQVIETTKNYEEKITDMSQSELL
QYQKTVYLTIMSSMSSDEAVHKLLKLNFKSKIKNKTKNKTKTKTNSNDNEILADMVIKCCSQEKTYLKYYGIIGEKLISRNDHWHNLFIKLFKYYYDIIENFETNSLRN
LGKFFGHLFASDKLALDQAWSNIKLTEQDTNPAKRILLKFIFQEMIEELGINEVKERLINDDYLKPYIKGIFPVINVDGKDADAIRFSINFFTAIGLGVLTEEMRYVLD
NLSEPEEEDDSGRSRSRSYSRSASSSSYNSRSRSYSSSQSRSSRSPNPRGRQRFKTKKHNHNESRTPSRENLKRKSSESVTDRNGNNNNNNNNNLQDLLNNLK

>ScCwc22 [Saccharomyces cerevisiae S288C] (YGR278W1)
MSTATIQDEDIKFQRENWEMIRSHVSPIISNLTMDNLQESHRDLFQVNILIGRNIICKNVVDFTLNKQNGRLIPALSALIALLNSDIPDIGETLAKELMLMFVQQFNRK
DYVSCGNILQCLSILFLYDVIHEIVILQILLLLLEKNSLRLVIAVMKICGWKLALVSKKTHDMIWEKLRYILQTQELSSTLRESLETLFEIRQKDYKSGSQGLFILDPT
SYTVHTHSYIVSDEDEANKELGNFEKCENFNELTMAFDTLRQKLLINNTSDTNEGSNSQLQIYDMTSTNDVEFKKKIYLVLKSSLSGDEAAHKLLKLKIANNLKKSVVD
IIIKSSLQESTFSKFYSILSERMITFHRSWQTAYNETFEQNYTQDIEDYETDQLRILGKFWGHLISYEFLPMDCLKIIKLTEEESCPQGRIFIKFLFQELVNELGLDEL
QLRLNSSKLDGMFPLEGDAEHIRYSINFFTAIGLGLLTEDMRSRLTIIQEVEDAEEEEKKLREEEELEKLRKKARESQPTQGPKIHESRLFLQKDTRENSRSRSRSPFTVE
TRKRARSRTPPRGSRNHRNRSRTPPARRQRHR

>LkCwc22 [Lachancea kluyveri CBS 3082] (SAKL0H00462g)
MNPDKISKDIENDVVIQKENWERLATHIATVVGSLKPSTVEQCYRNLLQSNLIRGKEILASSVLKYQTDFNQGMASAALVALVNSEFPEFGDLVIREAIARFISGYKSN
DKRECYSMMSLISYSFCYQVVHEILILQLIHLLMERPTNDSVDLVISLLRQCGKSLVEVSKVAHTVTYEKLRDLLQEGRLSKRTAHSVEDLFELRRFNYDSVSLNLISL
PAHKINTHTIILDLGKEESFKQNMGLAEFQYDPFFFENESKYELFKKQLLETDAEEDTIPITTVKDMTNNDEVAFKKTIYLILKSSLSGDEAAHKLLKLRASDGQKHKI
VDILVSSCSQEATYSKFYGITAERLCSSHRSWKPAFSKIFADNYENVTQFEPFQLRNVGKFWGHILASDYIGFEVFEQVHMNEEETTSSGRVYLKFLFQELVADLGIKE
LVERLREEYIQPFLANIFPLDNPEKTRFSINYFTAIGLGALTENMRRELMRTEEEETLRGKSNVDTLLHEGRLARPVRGRNEVSISLGSQNINRSPTPVKRRDRSITPP
SRNRNRSITPTKRSNRSITPPRRQRSRSPPRNRPTTSNRFSNSPSDFARPRDNGWGDRPNKKNR

>KlCwc22 [Kluyveromyces lactis CBS 2359] (KLLA0C14025g)
MNSEEHQQHEWEQSRLILSHVLTNIDSANILGNLRDLIEVNILRHRRLFVATLLSLQCENDKDSAFAGLVKLIDHYLPSIGFLTGRECVLRLIDAVYRSDKKVYFNMAS
LLSFLIIDEVIDEGTAFALIYFLLRNKTDDNIILICHILCLLGQQLQAFDKESEADIAEKLRLIYQDRYTQKHTYKALNRLFDTRRRSYRNVIKNVNIPTVENNVHEVV
VDFSNDHPAMDLDGFHLDDKYELINEQYEEIKSEIIKELLESKDVKNSLNDMTDSENTEFKKKVYLILKGSLSGDEAAHKLLQLRLNDAKKNEVADTITKACAQEPTYS
KFYGILTERLTSFHVSWQHSFSAVFKENYQTIADNDPSNIRNLGKFWGHALATECIPFEVFEIVHMNERDSNAANRVFLKFLFQEMVVNLGIDALKKKLEAVELQPFLS
NLFPREAQDDIMFSINYFTAIGLGALTDNMRNILQQAEEEQRKRYSNAIKNSEVSERDNPRVPRMHPDRLRRNRSRSPISRRNRSRTPPRRRQ

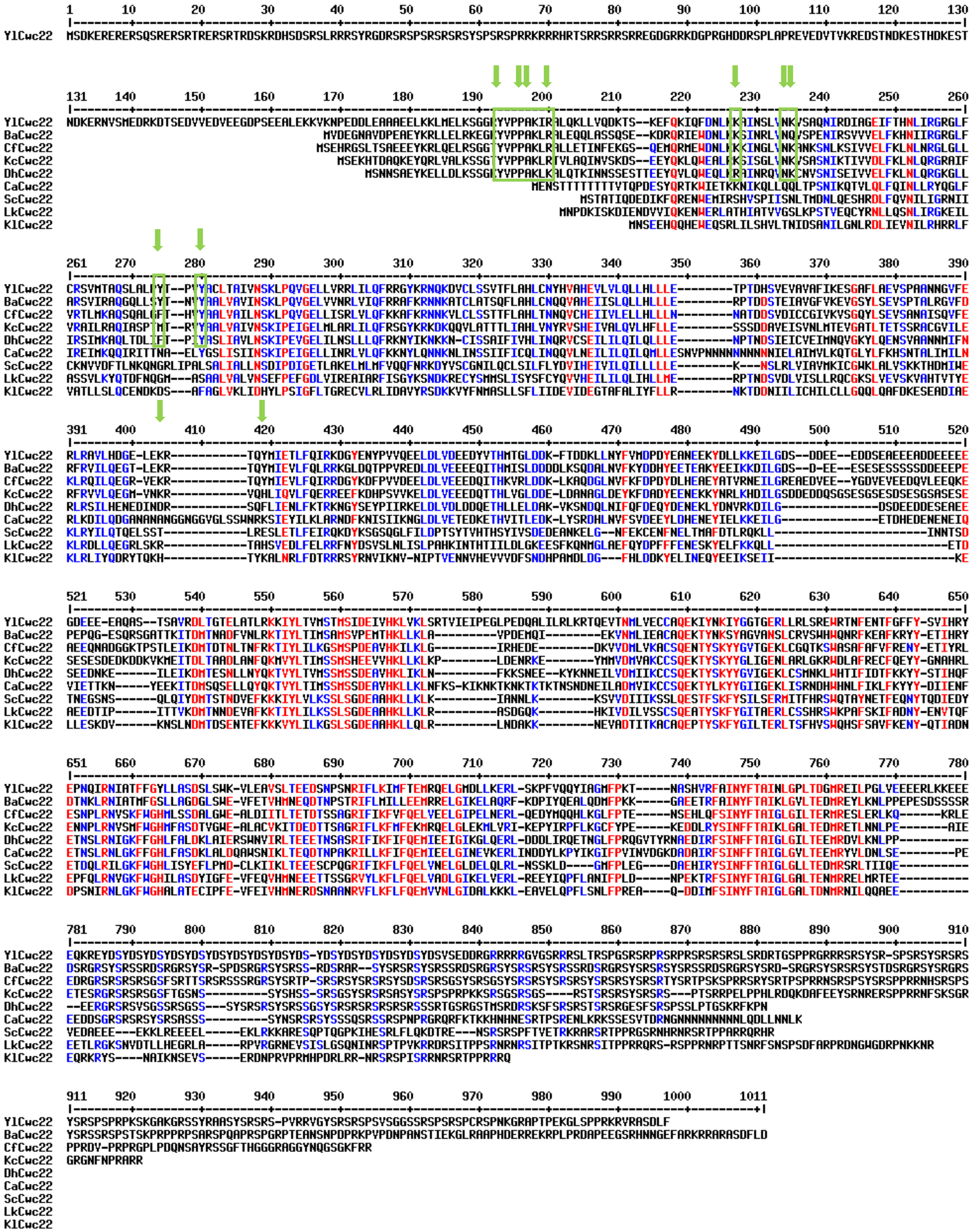

Alignment was performed with the Multalin server (http://multalin.toulouse.inra.fr/multalin/). Sequences for *Blastobotrys (Arxula) adeninivorans* (Ba), *Cyberlindnera fabianii* (Cf), *Kuraishia capsulata* (Kc), *Debaryomyces hansenii* (Dh), *Candida albicans* (Ca), *Saccharomyces cerevisiae* (Sc), *Lachancea kluyveri* (Lk) and *Kluyveromyces lactis* (Kl) were extracted from GRYC (http://gryc.inra.fr/)

↓ Conserved amino acid residues involved in the interaction with eIF4A3 (Y_{193}, P_{196}, A_{197}, R_{200}, K_{225}, N_{232}, K_{233}, Y_{272}, Y_{276}, K_{391}, and Y_{395} in *Y. lipolytica* Cwc22)

▯ Conservation of the amino acids involved in the interaction with eIF4A3 in the five first yeast species

Supplementary Figure S5: Sequences and alignment of Upf3 proteins

A. Sequence alignment of Upf3 in model genomes

```
>HsUpf3B [Homo sapiens] (AAI21018.1)
MKEEKEHRPKEKRVTLLTPAGATGSGGGTSGDSSKGEDKQDRNKEKKEALSKVVIRRLPPTLTKEQLQEHLQPMPEHDYFEFF
SNDTSLYPHMYARAYINFKNQEDIILFRDRFDGYVFLDNKGQEYPAIVEFAPFQKAAKKKTKKRDTKVGTIDDDPEYRKFLES
YATDNEKMTSTPETLLEEIEAKNRELIAKKTTPLLSFLKNKQRMREEKREERRRREIERKRQREEERRKWKEEEKRKRKDIEK
LKKIDRIPERDKLKDEPKIKLLKKPEKGDEKELDKREKAKKLDKENLSDERASGQSCTLPKRSDSELKDEKPKRPEDESGRDY
REREREYERDQEHILRERERLKRQEEERRRQKERYEKEKTFKRKEEEMKKEKDTLRDKGKKAESTESIGSSEKTEKKEEVVKR
DRIRNKDRPAMQLYQPGARSRNRLCPPDDSTKSGDSAAERKQESGISHRKEGGEE

>DmUpf3 [Drosophila melanogaster] (NP_726375.1)
MAETFAEEKPKEKSKASRRKDKKDKTNQIVKIVMRHLPPTMTEAQFLDQVGPLPENDSYYYCKADWSLGQEATCRAYIDMSSK
DIGEVVQFRDRFDGYVFVDHKGVEYMAIVEYAPFQCFLKNKARNDDSKVNTIESEPHYQEFIKRLAQEREEASRMGDVKIDFN
FERRTEEKVKSTPLLQYLANKKEKRREEARRRNEEKRKQREEQKLLRLAAQSDASKLKEAEGGGGGGDTKKPAKKDVKDPQSS
GSQANDAKASRSKRRTERDQRRREEHEQRKLVKRDKKKDDGQGKQDKQDKQDKGKPSSGKQNKNSKSMDIVILKKANKPESNE
SLDVPSTSNAAEASKEKPATENTETPEAAVKVFTPAARGGRKEKRTGADKQPAGSAHETAEEAIKAAQFRASEERRIRNKDRP
SIAIYQPKARIRASDELPQGAGGKDGSDGEASVVEEKTSKRNKRPNRRNKPKPKEVKECELETRRFSKSSESSTSVK

>YlUpf3 [Yarrowia lipolytica CLIB122] (XP_499932.1, this study)
MEFDRSQIFAPVKADTVPRFAQRPRPAAPGLLDQLPSTDIGQPKNNDRLRILVRGVPDYVSSEEVEKHLIKPFQELVDSWYLI
RQDFVGGLDLDLDQESLTESCTRVYVQLHNREGVQKFHASVNGDVVIAKNWSQSPDEKSDTETISDNIPTPLKVEYSPLYYFT
PDPTTTDIMAGTIESDPLYIKFCENPDQLLFEGGEGRILEPVIAPTDFEVGSIVPVGVPLAKIDIPEEERVTLPEEKKSNKKK
TKKGNKDKEKKLGPTDDSKRDSKHKEKDSTRVTSDDSSRESTPSEDKKKKRSRKKKLVTSDGANGTAPPSAPSAGTRETSDSV
PSNVEPPLPSVAISTIGAAPNNSNSTTSTQGKSKTKKKRVREPKDSRNSRESPAPREPSRESLTPRELGDSTPKDLRDTQTNR
KQKKKSDKPKKDSKAEANDSKPEPRLKQQPGKENQSKSKPRSQPQSQPAVDIPQTTNQSAAIQNGNERAPPKKKRTRTKRPAA
NGAAATPSDKASSAPAPQ

>CeUpf3 [Caenorhabditis elegans] (NP_741600.1)
MTDSKDGHVKVVLRRLPKYMTEHEVLEQISPLPEEVIGTYFHPANFSFDRCAYATLTVNFSEYCDSMMEFERRFDGYIFVDSR
GNDSAAVVEAASNQNFAKCDRNRMKEDTRVGAILTDKYYLDFCKKLEEERAIPILTLEQQIRKLNQPDDARTQIDKMETPLVK
YFFEKETGKRRDYDARRQRRDEKRAEKRDKVDKFEKHKNVLDILMKPSVPMASTSATTSKKDLKKEKPMTEKEKERWEKQDAK
RKERNMIRKQKFLDEKKKKHEEREQDGPRVPRDKKKERLAKPPRPSPAKTVGEQQGEDWIKKLTDPKSAPMKKKHDLSVTTKL
NKETDTRPRTAPSSTKLPTRATTSNHPKRPSTAPNH

>SpUpf3 [Schizosaccharomyces pombe 972h-] (NP_593705.1)
MAPDISKKRLPCKVLVFNLPPTLPEQVFLQSINSFLPHVEWHRFSKGKATVGTRSELLSFAYLKFQSATAVQEFFRVYQGHTF
IDKKNNTYRAIVTIAPYQKIPPSKVKADSLEGSLEQDPKFQEFKVQRESYSQTASNDDVIEKLQTSTPLLQYLAEKKNAVVEK
GKSKPSKKSVKAKKKLRLAEKPASNNSKAGKSSQESKKSSKAPAESAAAVIKEDKVSDRKKSKKKPKKTPVSNSTASQASENA
SDKKTKEKKSSGKQKIASKKKDQLTTDNV
```

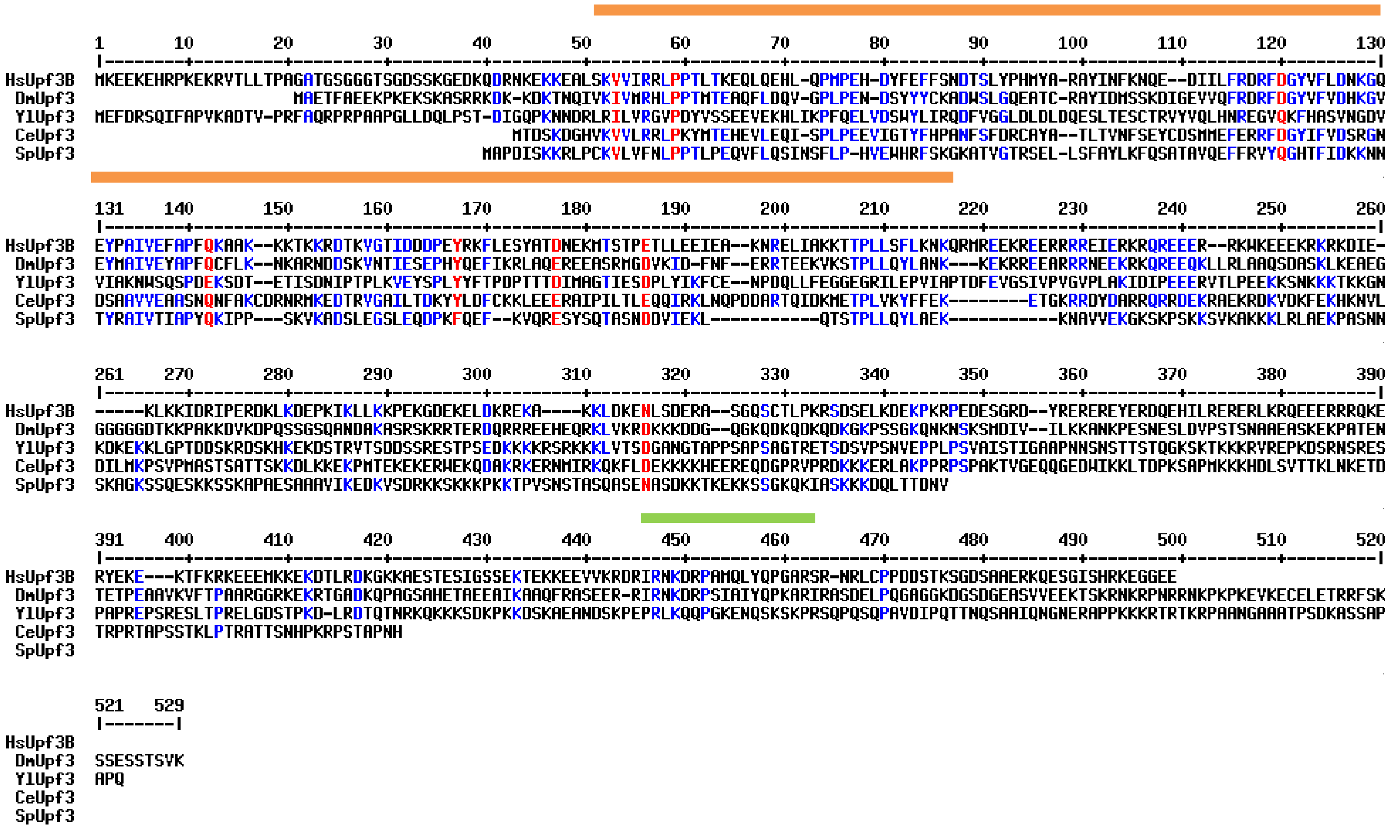

Alignment was performed with the Multalin server (http://multalin.toulouse.inra.fr/multalin/)

Amino acids that match the Smg-4/UPF3 family conserved domain (pfam03467)

Amino acids domain from I_{418} to R_{434} of human UPF3B that mediate interaction with EJC (Gehring *et al.*, 2003; Buchwald *et al.*; 2010)

B. Sequence alignment of Upf3 in Saccharomycotina

```
>YlUpf3 [Yarrowia lipolytica CLIB122]  (XP_499932.1, this study)
MEFDRSQIFAPVKADTVPRFAQRPRPAAPGLLDQLPSTDIGQPKNNDRLRILVRGVPDYVSSEEVEKHLIKPFQELVDSWYLI
RQDFVGGLDLDLDQESLTESCTRVYVQLHNREGVQKFHASVNGDVVIAKNWSQSPDEKSDTETISDNIPTPLKVEYSPLYYFT
PDPTTTDIMAGTIESDPLYIKFCENPDQLLFEGGEGRILEPVIAPTDFEVGSIVPVGVPLAKIDIPEEERVTLPEEKKSNKKK
TKKGNKDKEKKLGPTDDSKRDSKHKEKDSTRVTSDDSSRESTPSEDKKKKRSRKKKLVTSDGANGTAPPSAPSAGTRETSDSV
PSNVEPPLPSVAISTIGAAPNNSNSTTSTQGKSKTKKKRVREPKDSRNSRESPAPREPSRESLTPRELGDSTPKDLRDTQTNR
KQKKKSDKPKKDSKAEANDSKPEPRLKQQPGKENQSKSKPRSQPQSQPAVDIPQTTNQSAAIQNGNERAPPKKKRTRTKRPAA
NGAAATPSDKASSAPAPQ
```

```
>KcUpf3 [Kuraishia capsulata CBS 1993] (XP_022456435.1)
MSSIDVVMDQVLVQSGVESTLRQPVTKGDVNYNLLISNLTEQLDIHQEKHQALKLIVRLLPPLLTAEEFFNQVSSVLQPSWMK
ANYYVRGYYPSDTFKQPMHSRCYILFDSEEHLRCFVKETRSIPFHNSANLAQGHTLSEALLTKEFIPTMERATSEIMAFPSEY
DTFARADKPRVTPAFQAFENWLKITDNPAEKAQLAKQGFLTDVNWQLLKNRSKRSALKSKAKSRKDASEKKQDPQRREKSATP
DSKNQGSSKKKDDTKGTGNKSESAKKNPKKKQSGIEANGETKTEGKASRDKSASKQAKLKDKTETEKKEPKKNHGNKKSSAPK
SVGESTGVDPKAGPKADPTSSDKKPKKSKEASRPKSAPSALESEKPERPKKEKKKKVLSSGDITPGANDRKETGSEAKKPAKR
RDNKKDTKPDGNVKTETKLISRSEVPKKENGGEPKEPKPKKVLLMRRPKEVDGTTS
```

```
>CaUpf3 [Candida albicans SC5314] (XP_716346.1)
MASITPRRSGPAPGILSKSPSLSSTTTGTPTTTTTTTSSSTSVPIISPNNKFVIRLLPPSLTESEFLNQLATYYPYHASKICQF
YYIQGHYPKTNFEVPIYSRAYVNFSNQHDSLEFLNFLKEKPFENEFDSIIPVIEKALFHKMVDTSANTSQKTLKESKKNLKRD
DLVNNEIYKKFLMFINNEIDQFDLIKINKSTKKKESSKAVSSTSKSSKERKKLKAKKSSKEKATTVSREKSKEKDKSKEKTKD
KGKEKPEKSKNGEDVSKDKSVNKDTKGQEDVKLSKKSNGSKSTDEGTKEIKKRPRRSKKPKDTSEKTKKQTESKSGTTSDEKT
EVAKPSKATKSKANKPTSKSSKPESKDEQSSSTPPKKQSRKPKSQPKDSQQTTKVESQQLGLKPIKLLKRPESKE
```

>CfUpf3 [Cyberlindnera fabianii YJS4271] (CYFA0S09e01376g)
MDISSRRPPLEGPKLLTREDFEKQQQQQQQKKKTISAKSIPNASEKMSNGEDKQKRKRTRNKAKKNKGVKVVVRLLPPNLEA
DDFFKTTAPLTGPQAAFDSYYIKGHYPTKPFKEPEFSRVYISFKTPQHAEKFSKQFKDHAFEDDKESFVPNITASLLAKMPDS
QEKLMAPKTKLQDLPEFKKFMKFHAGEIEKPPSFLTSESEDKKRAEAQVSKKTDSNSGPEEDDKKTTDKKADKKEKKKKKKEK
KEKTKKKEKKKKGKPSSDPSSKPASAATEADKKERQQQPPNQKENQVESQKDNQKQSRKGKQKANRKAKDDNLKESESKKKIS
ENANIVQNQNQQSQPNNEAKKQKKKRVKPTKATRSAQTSPSPSYFPVLGESTTNSAPPKEAPKNKEQQAPATQMTKNQQDKTT
PVKKKKILMKRSSE

>LkUpf3 [Lachancea kluyveri CBS 3082] (SAKL0C08338g)
MEPVEPDKRPDALIKKNNRARGKRQDKRAQQPSERRPLPENGPTKRTDSAEGRSASGSRFRRRNRRAKKAGPPGHKLVFRSLP
PNLTESQFYSTIASIVDQGFVSTNCNDCYYVQGHYSKKPFKPPTYSRAYFTFRDMDTLQPFARKLANVKFVDDRDNAMVPTVA
ISSFVKKFRGEESNAMKRTKSHLEGSLQEDKLFQTFVKSLGLIEANRQEYQYSDLSIFSPLERELKRKQELLQKVRLQSEKAI
VELAGDGEQKKNKKNKKNKKNKKSKKSKKNNNNNGQPDVRERGHGDGDGDGDGDNNGDSGGNDGGGEKNRRRTGAEKGKGKLK
NKKNKKKKTEHDSGDPNNNNVVIIEAAGRRELEKRERLERKAAKKEKDATVAGAESGKKKQDHRKKAGRRDDGETFTGQPREY
GIPDVQLSKPNGADSVECKDMNLSSQPALTPDSKLNPKQKQKQKQKQKQKQKQQKQQKQKQQKQKQKQGQGQKSVP

>KlUpf3 [Kluyveromyces lactis CBS 2359] (KLLA0D03718g)
MLSKKRNRRRYRKRSADSSREFKLCIRSLPPNLTEEQFISTLQENNIDVHSFQKSFYYVQGHYSSKVFKKQTLSRAYIQLDSF
ETIQSLSKTIRTCRFIDDLDNSMIPDLQISPFVKKWVSPNEVNPIQGTIQKDHVFQTFVKSWKLINEDETNSLQFKNLSVIAP
LRKELEREEKMEEELNGRKQRALIELAGDPTSEEKKKRIKKIKKKLRLLEKKRKLKRAKAKNKDNPDEKDVSKDKSEVEKPKK
SKSKHKSKSKSKPKSKSEPKSSKFDTATTEKSAVTSEEKPKKKILLKPKKDDVPKPEMPADSEVTKRARRKKSKKHKDKDKDV
KKDLTSKETKETPSKVTPKVKIKSKSANVEEMKSEKRKQENTKPEKNPSSESDKKAKSKRKSRKGRQSDGPKHKDKPT

>BaUpf3 [Blastobotrys adeninivorans LS3] (ARAD1D01606g)
MTEDEPGVERRSPVESPAHTPDAESLESQKGRKSPDKPAPAPSADSTEERKTQPEAPAQARPATFRDKVVIRKLPPALSEEKF
MEYAEGGEWFNDSICSLWYYVPGRVPKKEGKVPVYSRAYVAFKSPDLLRDFFRSFKRIVLPRILEEFGLSTSSAVPTVEYAPY
QAKFEKSVAPSTAGTIESDAVYKSFVKHLEDPTNEILSLVEPSKPKAKEGQDHRKEQGKKGKSRNRKPKSKKNKEASRDGKEE
EGSKGKDPKAPKDPKAKREPKEPRAPKETKEPKEPKEPKEKKERPKKPKNKGRGAQSQHPVQSDAQSQPQSQPQGGNGLPKGP
RGEKPKAPKGDTSVEQANGDKGEAKKRQRPPRPRRKKPGNGATDVTKSDAPA

>DhUpf3 [Debaryomyces hansenii CBS 767](DEHA2B11792g)
MSSVNGNSRTPTPKVGGKSTYINLKLTVRLLPPTLTKDQFIEQLNNYTDLLKDGSIIDDYYVQGCYPTKPYEKPTYSRAYLLF
KNQTSLDQFMKEINGKSFIELETNDSLIPAIGKSLYNKMPIERPYNTATPSKKFEDDEFYKEFLSQLEANTNESFDIVSINKK
LKKRNKDKKKKERDPKKKEDKKGKAKEEKKPAKPKSGKATDKTGDSTKAPPKAKKRNRNKKPEGENTNDKQKQKQPTDAKESS
DAPAKQPPKPKNKPHDSSKGKQDPKAKSKQDSKQKPKLAQDTKQKSNQDSSASNANTPKNPKIKIKQKPKGQNPKPNTGLANN
A

>ScUpf3 [Saccharomyces cerevisiae S288c] (YGR072W1)
MSNVAGELKNSEGKKKGRGNRYHNKNRGKSKNETVDPKKNENKVNNATNATHNNSKGRRNNKKRNREYYNYKRKARLGKSTEN
EGFKLVIRLLPPNLTADEFFAILRDNNNDDGDKQDIQGKLKYSDWCFFEGHYSSKVFKNSTYSRCNFLFDNLSDLEKCANFIK
TCKFIDNKDNITIPDMKLSPYVKKFTQTSKKDAALVGTIEEDEIFKTFMNSMKQLNENDEYSFQDFSVLKSLEKEFSKSIELE
NKIAERTERVLTELVGTGDKVKNKNKKKKNKNAKKKFKEEEASAKIPKKKRNRGKKKRENREKSTISKTKNSNVVIIEEAGKE
VLKQRKKKMLLQEKLKISNSSQPQSSSAQTQPSFQPKENLFVPRVKILHRDDTKK

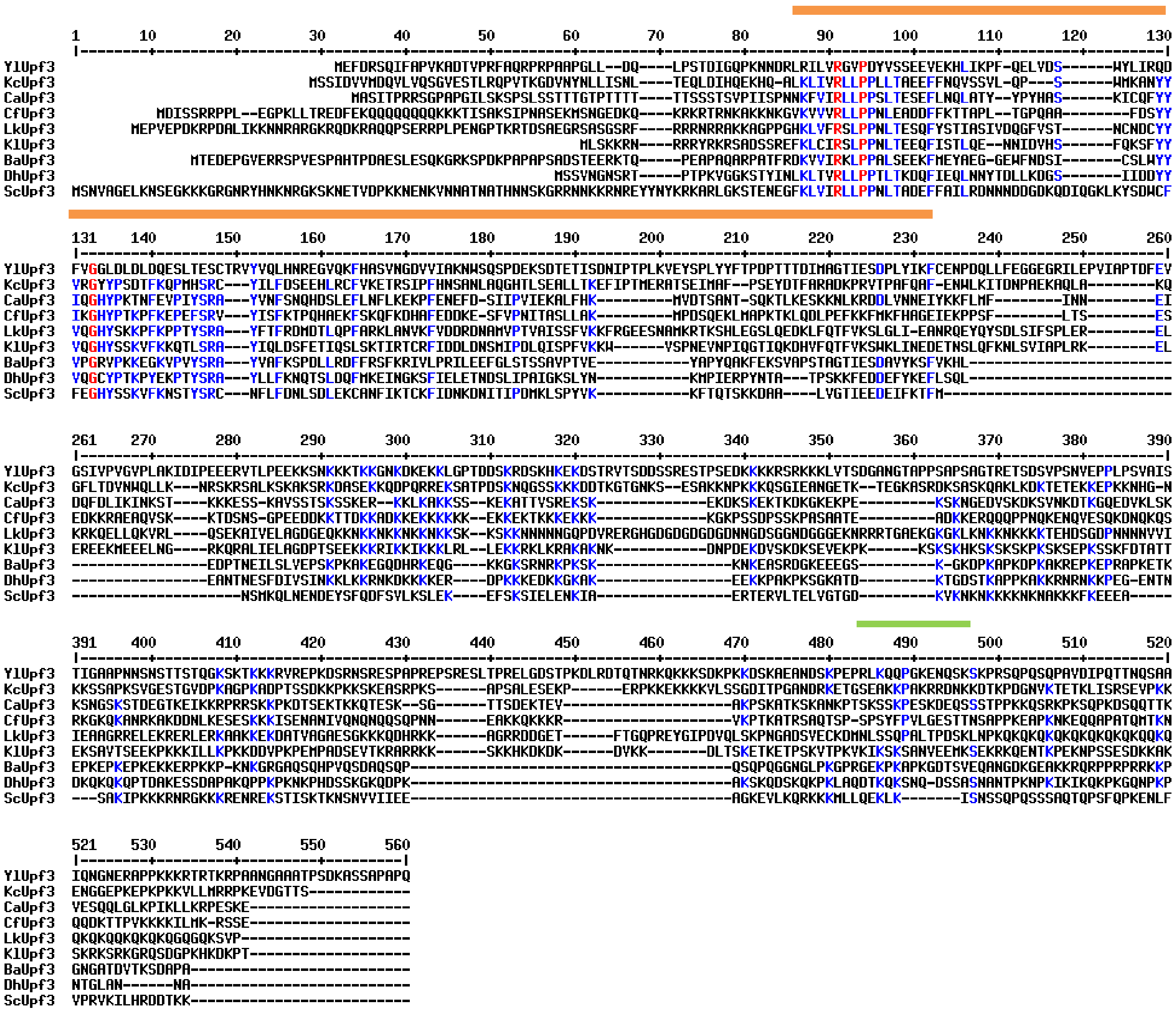

Alignments were performed with the Multalin server (http://multalin.toulouse.inra.fr/multalin/). Sequences for, *Kuraishia capsulata* (Kc), *Candida albicans* (Ca), *Cyberlindnera fabianii* (Cf), *Lachancea kluyveri* (Lk), *Kluyveromyces lactis* (Kl), *Blastobotrys (Arxula) adeninivorans* (Ba), *Debaryomyces hansenii* (Dh) and *Saccharomyces cerevisiae* (Sc) were extracted from GRYC (http://gryc.inra.fr/)

Amino acids that match the Smg-4/UPF3 family conserved domain (pfam03467)
Amino acids domain that mediate interaction with EJC

C. Conservation of the region that mediates interaction with EJC

```
HsUpf3b    418    IRNKDRPAMQLYQPGAR    432
DmUpf3     409    IRNKDRPSIAIYQPKAR    425
YlUpf3     439    PRLKQQPGKEN-QSKSK    454
```

Supplementary Figure S6: Sequences and alignment of Pym proteins

A. Sequence alignment of Pym in model genomes

```
>HsPym [Homo sapiens](NP_001137325.1)
MATPYVTDETGGKYIASTQRPDGTWRKQRRVKEGYVPQEEVPVYENKYVKFFKSKPELPPGLSPEATAPVTPSRPEGGEPGLS
KTAKRNLKRKEKRRQQQEKGEAEALSRTLDKVSLEETAQLPSAPQGSRAAPTAASDQPDSAATTEKAKKIKNLKKKLRQVEEL
QQRIQAGEVSQPSKEQLEKLARRRALEEELEDLELGL

>DmPym [Drosophila melanogaster] (NP_726372.1)
MSTYLQSSEGKFIPATKRPDGTWRKARRVKDGYVPQEEVPLYESKGKQFVAQRQAGVPPGMCPLLAAESKKEREKQERTRAKK
QEKESGRQPKAPAPGVLVMPPSTCPPPKVSQQQQQQQQQPSGSRDINSISKTLEDTLKLDAAQEVVDPAKQLKKLRKKIREIE
QIESRIQAGEQKKLDKDQLDKVKKKSEILRQIKDLESTPRS

>YlPym [Yarrowia lipolytica CLIB122] (XP_500390.1)
MASLTGTYEKDGERVVGGTVRSDGSVRPIAKVRPGYIAKEDVPKYKPRGARERDARLGIVSAESPIARVETQASGADDNKSAI
KARIQAAKAEFERKKREKEKGPRKEEKQMVEESVIFDNSGKSPDDISNMIAAVDNLDIRSTPDTTAPTKTSTTTSKLQSKHKK
REDKTERSDSTQIQVESQAESESKSQVESPTNLTTTPQSTQRIPLKSRHARKAESPTEEKSSNNANKTSGYIPPHKRK

>SpPym [Schizosaccharomyces pombe 972h-] (NP_596157.1)
METNGSFSGARLVDGKWIIPESRRKDGSVRRERAVKPGYTAPEDIKRYRPGRGNFASLEKQMKKLQLSNDASTSKSIDRPPIS
ELEKEKLERPLSNKKKEKNDHKAESLKHDYDSVGEKRISKDSVKHLDKTYSSIDSKKDFKYNFPKTQAPEWRRGAKPLSKTSE
PSVYSEKMSKRENKKSINTVDKKTGYKEKE

>CePym [Caenorhabditis elegans] (NP_499261.2)
MSTKPAAAKTKPSCSEGGDLRVETENGETFITATQRADGTWRKARRVKGGYIPQDEQPKYQNKMQLEATNGRSSVPAGVNPRA
MASGSSRKPVSAIKANVCITPQDHFQKKIDLTKKKIEDIEGMESRIASGELVPQPNQVKKIARKQEYLDEIEKLTLEMKKL
```

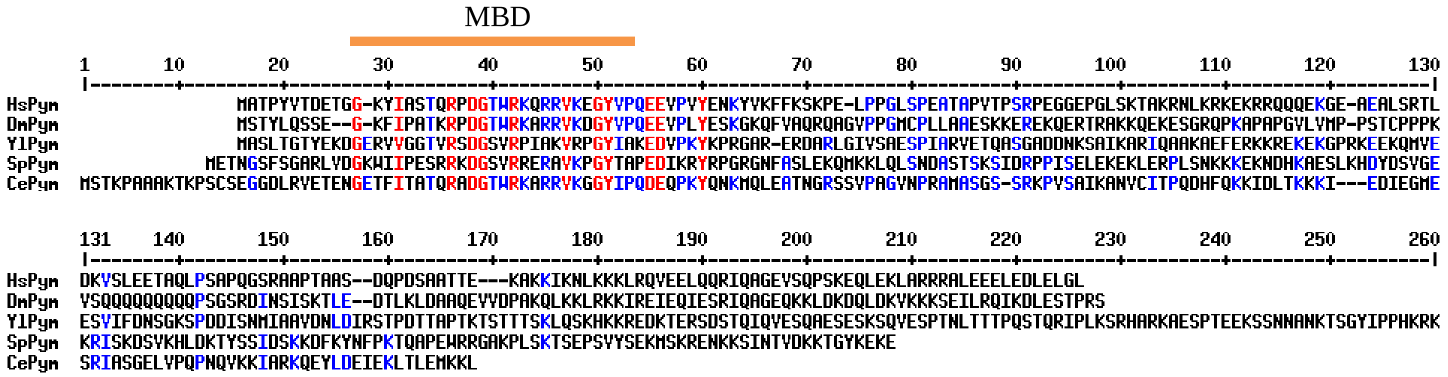

Alignment was performed with the Multalin server (http://multalin.toulouse.inra.fr/multalin/)
Mago-Binding Domain (MBD, pfam09282, Bono *et al.;* 2004)

B. Sequence alignment of Pym in Saccharomycotina

```
>YlPym [Yarrowia lipolytica CLIB122] (XP_500390.1)
MASLTGTYEKDGERVVGGTVRSDGSVRPIAKVRPGYIAKEDVPKYKPRGARERDARLGIVSAESPIARVETQASGADDNKSAI
KARIQAAKAEFERKKREKEKGPRKEEKQMVEESVIFDNSGKSPDDISNMIAAVDNLDIRSTPDTTAPTKTSTTTSKLQSKHKK
REDKTERSDSTQIQVESQAESESKSQVESPTNLTTTPQSTQRIPLKSRHARKAESPTEEKSSNNANKTSGYIPPHKRK

>BaPym [Blastobotrys adeninivorans LS3] (ARAD1B14894g)
MSGIYTSEDGSRYTGGSVRADGTVRRARKIRPGYVPQEDVPKYVPRAKRQGNEGAILKGKSDDLADSLAKLSLNQVAERERDS
EKSEKQEGPDSERGPRRKIIDPAQGPNQARSRPEAEKKPRRMIVDPEKEPTGPKAGQSLEATGPKMSETPERRPRRMLIDPSQ
EPKTIQRSGQRISQSRTQTTNSESERRLRRMRIDPEQEGSSQSPSKPTERKPTPEGSGKYVPPWKRGS

>ChPym [Candida hispaniensis CBS 9996] (OLHI0F15544g)
MSPADTHQSLDAVIGGTRRADGSLRPIVKIRPGHIAPEEKPKYQPPHLRPKKCAETEKQINKEFLSRPEQRNRESREGSSLAK
SGHHDNRSDCSISTRSLIQQKIQLAKQKFKQKQSGIDEITKDLGSLSVSTADTNQSETKPRDNGLQGEEELNPLNINDPVSDE
KFTTNASVSISGEGENKMETTRRDVDKIGSVDAIGGVDAIGGVDAIGTESTNGKECKESSQILKKPNNTSESERKNVNNT
```

```
>GcPym [Geotrichum candidum CLIB 918] (CDO57549.1)
MSDSTLPSGIREDENGTRIVGGTVRSDGTVRKTYKVRPGYVPQEDVAKYVAPKRSRVPGPDNGNRVASPHLRTINNILAGSGD
PAGGRPSRARLPKKTEDEFPALGQKPPASKPVDAVVDMLSSLDINQKDKKASSQSHADKLTGSATENPTSRVPNAEVAQSESK
PDFDKKSDSKSLTPFKAQDKPGVSSIINNTPEAPSTASNDVKKPAAGKYVPPWKRGT

>CfPym [Cyberlindnera fabianii YJS4271] (CYFA0S05e03180g)
MVSEEGKSTAGVHLDAEGNRVVGGSVRADGSVRKIVKVRPGFTPQEDVKRYNVREAREERLRSTSQIYQGGKAIQNASRDPQR
PSRFSGLNNFLKGVASEDAKGTRRSESSSGPLHIPPNHRHSVATQQSDDTDISNSLAKLSIEGDQKGDVDKKSVAKHQESSSP
DSIPNQKSKKAYVPPQRRKKYTLADLDSQKT

>WcPym [Wickerhamomyces ciferrii NRRL Y-1031 F-60-10] (XP_011276933.1)
MSSESTKSTQVSGVDSNGDGIIGGTVRADGSVRKTLKVRPGFTPKEEVKRYDVRERRRQREEEKTEEKTKNEEVIRNPGSRPN
RQFNAINNILKANSSKPQKKEDKSIDDVESSLRKLGLNSKGNGVNSVSNVNRRKETICLETTETKDQEEPTKPKKYIPPSRRN
K
```

MBD

```
       1        10        20        30        40        50        60        70        80        90        100       110       120       130
       |--------+---------+---------+---------+---------+---------+---------+---------+---------+---------+---------+---------+---------|
YlPym      MASLTGTY-EKDGERVVGGTVRSDGSVRPIAKVRPGYIAKEDVPKYKPRGARERDARLGIVSAESPIARVETQASGADDNKSAIKARIQAAKAEFERKKREKEKGPRKEE--KQMVEESVIFDNS
BaPym         MSGIYTSEDGSRYTGGSVRADGTVRRARKIRPGYVPQEDVPKYVPRAKRQGNEGAILKGKSDDLA----------DSLAKLSLN-QVAERERDSEKSEKQEGPDSERGPRRKIIDPAQGPNQ
ChPym         MSPADTHQSLDAVIGGTRRADGSLRPIVKIRPGHIAPEEKPKYQPPHLRPKKCAETEKQINKEFLSRPEQRNRESREGSSLAKSGHHDNRSDCSISTRSLIQQKIQLAKQKFKQKQSGIDEI
GcPym    MSDSTLPSGIREDENGTRIVGGTVRSDGTVRKTYKVRPGYVPQEDVAKYVAPKRSRVPGPDNGNRVASPHLRTINNILAGSGDPAGGRPSRARLPKKTEDEFPALGQKPPASKPVDAVVDMLSSLDIN
CfPym  MVSEEGKSTAGVHLDAEGNRVVGGSVRADGSVRKIVKVRPGFTPQEDVKRYNVREAREERLRS-TSQIYQGGKAIQNASRDPQRPSRFSGLN--NFLKGVASEDAKGTRRSESSSGPLHIPPNHRHSVAT
WcPym  MSSESTKSTQVSGVDSNGDGIIGGTVRADGSVRKTLKVRPGFTPKEEVKRYDVRERRRQREEEKTEEKTKNEEVIRNPGSRPNR--QFNAIN--NILKANSSKPQKKEDKSIDD-----VESSLRKLGLN

       131      140       150       160       170       180       190       200       210       220       230       240       250 254
       |--------+---------+---------+---------+---------+---------+---------+---------+---------+---------+---------+---------+---|
YlPym  GKSPDDISNMIAAVDNLDIRSTPDTTAPTKTSTTTSKLQSKHKKREDKTERSDSTQIQVESQAESESKSQVESPTNLTTTPQSTQRIPLKSRHARKAESPTEEKSSNNANKTSG-YIPPHKRK
BaPym  ARSRPEAEKKPRRMI-VDPEKEPTGPKAGQSLEATGPKMSETPERRPRRMLIDPSQEPKTIQRSGQRISQSRTQTTNSESERRLRRMRIDPEQEGSSQSPSKPTERKPTPEGSGKYVPPWKRGS
ChPym  TKDLGSLSVSTADTNQSETKPRDNGLQGEEELNPLNINDPVSDEKFTTNASVSISGEGENKMETTRRDVDKIGSVDAIGGVDAIGGVDAIGTESTNGKECKESSQILKKPNNTSESERKNVNNT
GcPym  QKDKKASSQSHADKLTGSATENPTSRVPNAEV-AQSESKPDFDKKSDSKSLTPFKAQDKPGVSSIINNTPEAPSTASNDVKKPAAGKYVPPWKRGT
CfPym  QQSDDTDISNSLAKLSIEGDQKGDVDKKSVAKHQESSSPDSIPNQKSKKAYVPPQRRKKYTLADLDSQKT
WcPym  SKGNGVNSVSNVNR------------RKETICLETTETKDQEEPTKPKK-YIPPSRRNK
```

Alignment was performed with the Multalin server (http://multalin.toulouse.inra.fr/multalin/)

Mago-Binding Domain (MBD, pfam09282, Bono *et al.;* 2004)

Supplementary Figure S7: Sequences and alignment of Aly-REF proteins

A. Sequence alignment of Aly/REF in model genomes

```
>HsAly/REF [Homo sapiens] (Q86V81.3)
MADKMDMSLDDIIKLNRSQRGGRGGGRGRGRAGSQGGRGGGAQAAARVNRGGGPIRNRPAIARGAAGGGGRNRPAPYSRPKQL
PDKWQHDLFDSGFGGGAGVETGGKLLVSNLDFGVSDADIQELFAEFGTLKKAAVHYDRSGRSLGTADVHFERKADALKAMKQY
NGVPLDGRPMNIQLVTSQIDAQRRPAQSVNRGGMTRNRGAGGFGGGGTRRGTRGGARGRGRGAGRNSKQQLSAEELDAQLDA
YNARMDTS

>DmAly/REF [Drosophila melanogaster] (NP_651968.1)
MVDKIEMSLDDIIKSTRSQKKPQAARGGPGGARKTGGQQRFAGGARRGGANAGGSPRKPGSVLKGPRGGVAAGAVQKAKFPRG
DVNSAWKHDMYDGPKRGAVGGGSGPTRLIVGNLDYGVSNTDIKELFNDFGPIKKAAVHYDRSGRSLGTADVIFERRADALKAI
KQYHGVPLDGRPMTIQLAVSDVAVLTRPVAATDVKRRVGGTAPTSFKRGGGQAGGTARRGFKRPVGGKPAAGGQRRERKAPPT
AEELDAELDSYINDMKI

>YlAly/REF [Yarrowia lipolytica CLIB122] (YALI0F07909g)
MDQALDEIIAQPSRRDDRPPRPERSRRSDRGDRRGRDYDDRRSARGRGDRERGRFVDKRYRDALPNTVLLIRNLHYELTENDL
YDLFNKVGRVDDVEIHYDRSGRSLGDANVVFSRPEDAQDAIDKFDGKRAAGLKIEVKIDHRRDPALDLSERFGPRDRSLSPGR
DRPYGGYREDRPPRRGRGGRGGRGRREGGSSTGRIPKEKKTVEELDAELDSYMQD

>CeAly/REF [Caenorhabditis elegans] (NP_001076697.1)
MVKATNVDLSLEDIISKTRKTTGSIQKKSFGGARRGNTRPTGLPRRSGGSGGWKDLDAVQNHGISSRGNDSKVIRVNISNLAP
TVISSDLEELFGDYRLSSVSVNFNEHGDSLGTGDISLTKRDAERLVQKFSGVALDGKIMKFAVIDSSNMAGRVDFGNRSRSSP
GRSSRGFQSGPRRNNGKPEDFLRDGVHAGDIKRGGAGRGGFRKGGRGGARDAKPKKTEAELDAELEAYMAKRNA

>SpAly/REF [Schizosaccharomyces pombe 972h-] (NP_595161.1)
MSLEKSLDEIINERTNGFDHKHSRRRGSQNRISKKSRLTYKFKRASKEHNSSPDDGPWQHDLDQEQDAHPRTTHLQKRQHSEN
RFGVRVENLHYQVLEKDVLSLFENFHPIRVIMNYDRAGRSEGSCDVYFETSQDAEDAQKTLQSTNLKGSEIQISKKSPPSLFD
RISDMPHSARKPSRSSRSNRGFNRSSKKDDRSFRSSSKKSSNNSISHEDLDKELDEYAMSFHAASTVSSHSSQDFTPSIANAH
EKNEPVAPSKDSNLTEEMDLQMEAV
```

```
           1       10        20        30        40        50        60        70        80        90        100       110       120       130
           |--------+---------+---------+---------+---------+---------+---------+---------+---------+---------+---------+---------+---------|
HsAly/REF  MADKMDMSLDDIIKLNRSQRGGRGGGRGRGRAGSQGGRGGGAQAAARVN-RGGGPIRNRPAIARGAAGGGGRNRPAPYSRPK-QLPDKWQHDLFDSGFGGGAGVETGG-KLLVSNLDFGVSDADIQELFA
DmAly/REF  MVDKIEMSLDDIIKSTRSQKKPQAARGGPGGARKTGGQQRFAGGARRGGANAGGSPRKPGSVLKGPRGGVAAGAVQKAKFPRGDVNSAWKHDMYDGPKRGAVGGGSGPTRLIVGNLDYGVSNTDIKELFN
YlAly/REF  M----DQALDEII-AQPSRRDDRPPRPERSRRSDRGDR-------------------------RGRDYDDRRSARGRGDRERGRFVDKRYRDALPNTV------------LLIRNLHYELTENDLYDLFN
CeAly/REF  MVKATNVDLSLEDIISKTRKTTGSIQKKSFGGARRGNT---------------------------RPTGLPRRSGGSGGWKDLDAVQNHGISSRGNDSK--------VIRVNISNLAPTVISSDLEELFG
SpAly/REF  M--SLEKSLDEIINERTNGFDHKHSRRRGSQNRISKKS------------------RLTYKFKRASKEHNSSPDDGPWQHDLDQEQDAHPRTTHLQKRQHS----ENRFGVRVENLHYQVLEKDVLSLFE

           131     140       150       160       170       180       190       200       210       220       230       240       250       260
           |--------+---------+---------+---------+---------+---------+---------+---------+---------+---------+---------+---------+---------|
HsAly/REF  EFGTLKKAAVHYDRSGRSLGTADVHFERKADALKAMKQYNGVPLDGRPMNIQLVTS-----QIDAQRRP--AQSVNR--GGMTRNRGAGGFGG------GGGT---------------------RRGTRG
DmAly/REF  DFGPIKKAAVHYDRSGRSLGTADVIFERRADALKAIKQYHGVPLDGRPMTIQLAVS-----DVAVLTRPVAATDVKRRVGGTAPTSFKRGGGQ------AGGT--------------------ARRGFKR
YlAly/REF  KVGRVDDVEIHYDRSGRSLGDANVVFSRPEDAQDAIDKFDGKRAAGLKIEVKIDHR-----RDPALDLSERFGPRDRSLSPGRDRPYGGYRED------RPPR--------------------RGRGGRG
CeAly/REF  DY-RLSSVSVNFNEHGDSLGTGDISLTKR-DAERLVQKFSGVALDGKIMKFAVIDSSNMAGRVDFGNRSRSSPGRSSRGFQSGPRRNNGKPEDFLRDGVHAGD--------------------IKRGGAG
SpAly/REF  NFHPIR-VIMNYDRAGRSEGSCDVYFETSQDAEDAQKTLQSTNLKGSEIQISKKSPPSLFDRISDMPHSARKPSRSSRSNRGFNRSSKKDDRSFRSSSKKSSNNSISHEDLDKELDEYAMSFHAASTVSS

           261     270       280       290      299
           |--------+---------+---------+--------|
HsAly/REF  GARGRGRGAG--RNSKQQLSAEELDAQLDAYNA-RMDTS
DmAly/REF  PVGGKPAAGGQRRERKAPPTAEELDAELDSYIN-DMKI-
YlAly/REF  GRGRREGGSSTGRIPKEKKTVEELDAELDSYMQ-D----
CeAly/REF  RGGFRKGGRGGARDAKPKKTEAELDAELEAYMA-KRNA-
SpAly/REF  HSSQDFTPSIANAHEKNEPVAPSKDSNLTEEMDLQMEAV
```

Alignment was performed with the Multalin server (http://multalin.toulouse.inra.fr/multalin/)
Amino acids that match a conserved a RNA recognition motif found in Aly/REF protein (cd12418)

B. Sequence alignment of Aly/REF in Saccharomycotina

```
>YlAly/REF [Yarrowia lipolytica CLIB122] (YALI0F07909g)
MDQALDEIIAQPSRRDDRPPRPERSRRSDRGDRRGRDYDDRRSARGRGDRERGRFVDKRYRDALPNTVLLIRNLHYELTENDL
YDLFNKVGRVDDVEIHYDRSGRSLGDANVVFSRPEDAQDAIDKFDGKRAAGLKIEVKIDHRRDPALDLSERFGPRDRSLSPGR
DRPYGGYREDRPPRRGRGGRGGRGRREGGSSTGRIPKEKKTVEELDAELDSYMQD

>ChAly/REF [Candida hispaniensis CBS 9996](OLHI0F36818g1)
MDQALDEIIARNPSAQDRRGEGRRSRGRSRRPARDHWDSSERRERYPDSRGSREVNDRDDYQGRRGRGQGYRRSEHKGTSRYR
```

DVEPGTVLLARNLHYELTENDLFDLFSKVGNVNDVEIHYDSSGRSLGTANVVYSSSQEAKEAIERFDGKKAAGLIIDVMVDSR
RDPQGDFSNRISNRERSRSPLSRPPKAGRKARKSKEELDAELDSYMGADQ

>GcAly/REF [Geotrichum candidum CLIB 918](CDO52513.1)
MDKSLDEIIEGRQHDRRYAGGRSDSRISKSHGRDFHTRRRPAPRITSMIKISNLHYELTEDDIYDLLSKVGEVRSVRLDYDIS
GRSIGTAIANFNSPYEAEEAVRRFNGRKAAGVIISVELYYHNSDRRAPRYDSRGGASSSHRRREPQPRRGNDDRDRSDAKPFR
RGERAPRKAKKTLEELDAELTSYMNGGSGDAPDTETSNTTSHHDNNEPSSNDHSNDNNNSSIQPNNSSNFGDSNASSTAAESS
NDQHPPAHKDDDEMVLD

>CfAly/REF [Cyberlindnera fabianii YJS4271] (CYFA0S20e02234g)
MSSLLEKSLDEIIGNDKPQRQSRGPRRDRYSRDTGYRSRRSRSRSGERGGSRPGDYRDRDSDRRPKRRGLEIEEGPLNSRSRA
QRLMQGRYYIKITNLKYDITESELELLLDRIGKLTFCYIEFDRSGRSTGTAYAQYYKVDDNTAAVESYNNRKAGGQIITVELI
KPLRIVALPSTEREERRGGRRERPRAKTAQDLDNELDAYMRGDEPATDRESRGSRDGGRRGGRPPREKKKTTEDLDRELEEYM
DNKPFASAE

>WcAly/REF [Wickerhamomyces ciferrii NRRL Y-1031 F-60-10] (this study)
MSNELERSLDELIHGGDEGSSRTKKFQRTRHRDRSRERYNNNRSNDSYRERYEAPRQTKIQRLSKGKSYLKIRNLHYEITEED
LKTLFEKVGETSFVYIEYNSTGRSTGVAYVGYLDLENNKVAVEKFDGKKAVGERISVEEIRPLNILSIAPRGPRGSRDDRDSR
GSRGRDVRGPRRDTRPKKPTLEDLDAELSSYMNGEELPKKEEHTNGDSRNPSRPRRQAKPTVEDLDKELDSYMNNKPFPATGS
E

>BaAly/REF [Blastobotrys adeninivorans LS3](ARAD1D33352g)
MDRTLDEIIASKPKGGVSKDTHRRGGRNAHRRDHRDPRARPRSRSEGLKTIMITNLFYELNEEDLESLFSKVGEVKKVKIQYD
RSGRSEGVAWVTFGDAHSAREAIELYNGKPAAGQRITITIAHGYGYERFSGNDFSERLAADRERDRRGTRRNRSSRRESRRPR
KKTQEELDAELDAYMNPNSVGNGSTEEPAPQQASEQPEQAPVQESTETAPTENAMALD

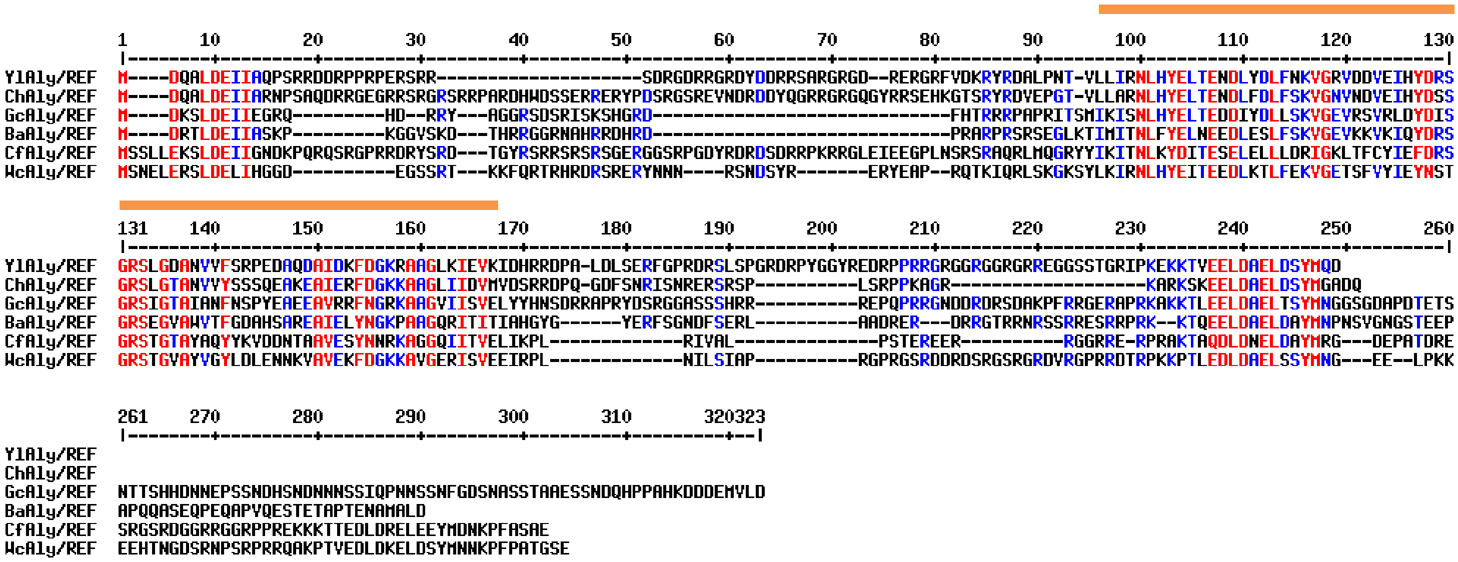

Alignment was performed with the Multalin server (http://multalin.toulouse.inra.fr/multalin/)
Amino acids that match a conserved a RNA recognition motif found in Aly/REF protein (cd12418)

Supplementary Figure S8: Intron retention in NMD and EJC mutants

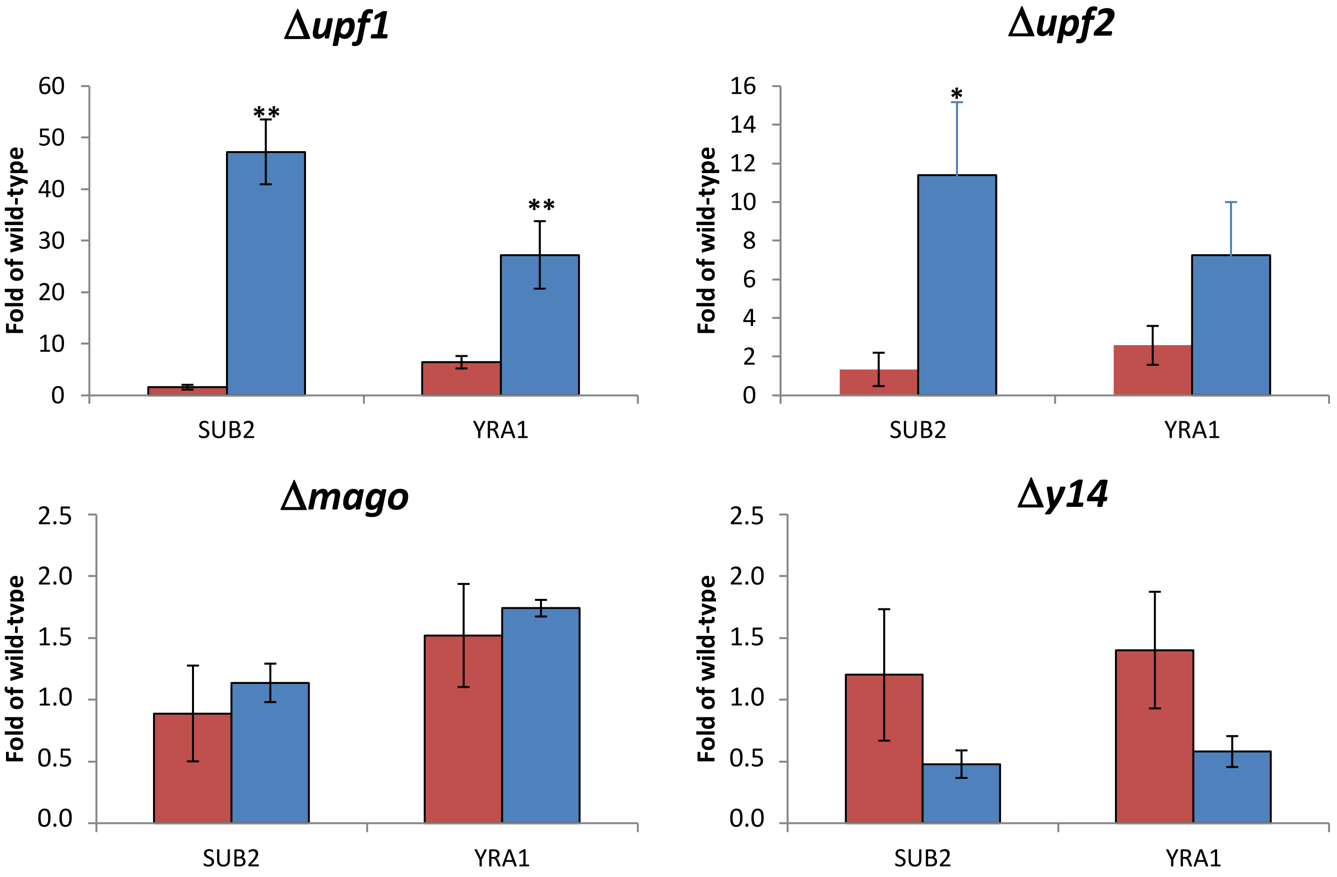

Quantitative reverse transcription PCR analysis of splice variants generated from the *YRA1*/YALI0A20867g and *SUB2*/YALI0A11157g genes in Δ*upf1*, Δ*upf2*, Δ*mago* and Δ*y14*. The transcript levels are normalized to the constitutively expressed *TFC1*/YALI0E14663g and are expressed relative to the wild-type strain. Three biological replicates were used and the error bars represent the standard error of the mean. Asterisks denote a significant difference, (*) $P \leq 0.05$ and (**) $P \leq 0.005$ comparing each sample with wild type. Levels of retained introns of *SUB2* and *YRA1* are represented in blue and exons in red.

Supplementary Figure S9: Heatmap of the fold-changes (in log2 scale) of the 199 differentially retained introns between the mutant and control strains

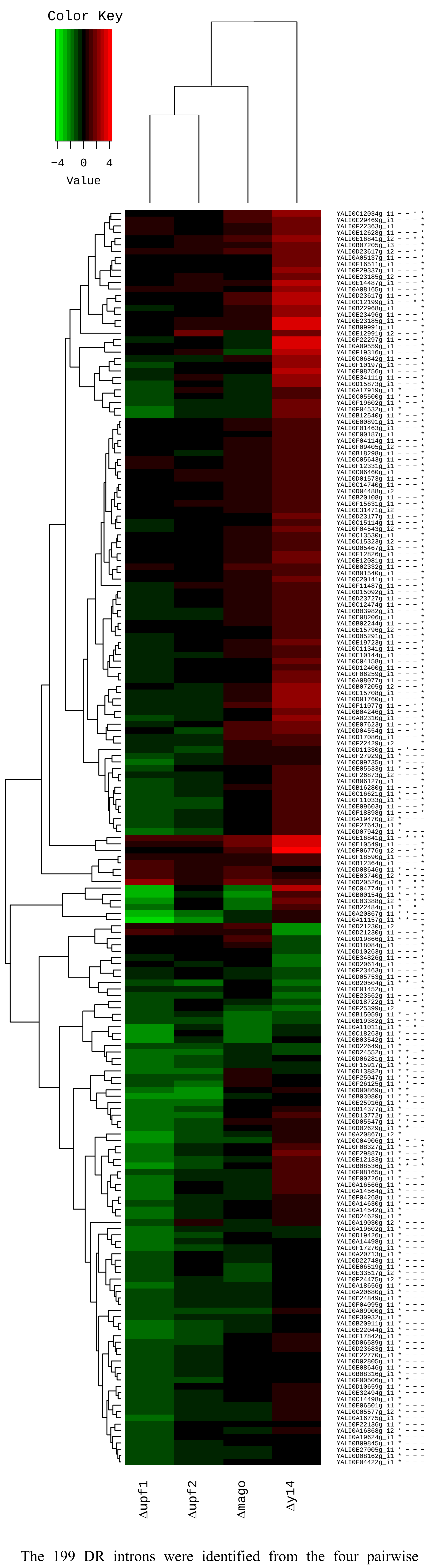

The 199 DR introns were identified from the four pairwise comparisons between the mutant and control strains, when decreasing significant fold-change threshold to 1. Red (green) indicates weaker retention (higher) in the mutant than in the control strain. Star and minus signs on the right, next to the intron names, indicate whether the observed variation is significant or not, respectively. Introns and mutants are clustered according to the fold-change values (distance: Euclidean, link: complete).

Supplementary Figure S10: Fold-changes (in log2 scale) of the 31 DR introns between Δupf1 and the control strain and whose corresponding genes are not differentially expressed

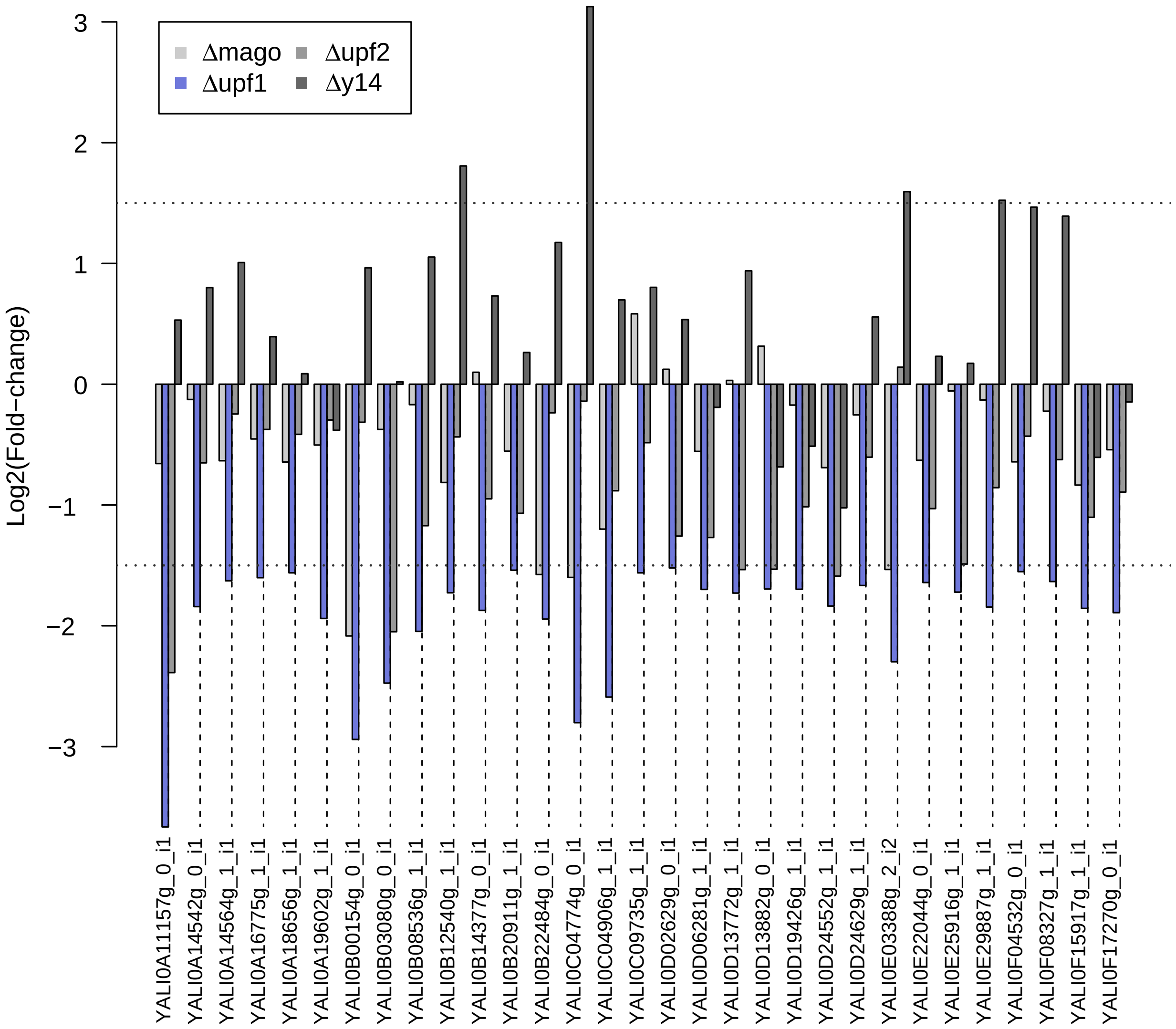

In blue, the fold-change values between Δupf1 and Δku70LEU2, in grey scale, the fold-change values for the three other pairwise comparisons. Horizontal dotted lines indicate the fold-change threshold considered to be significant (1.5).

Supplementary Figure S11: Intron structural parameters with respect to intron retention categories

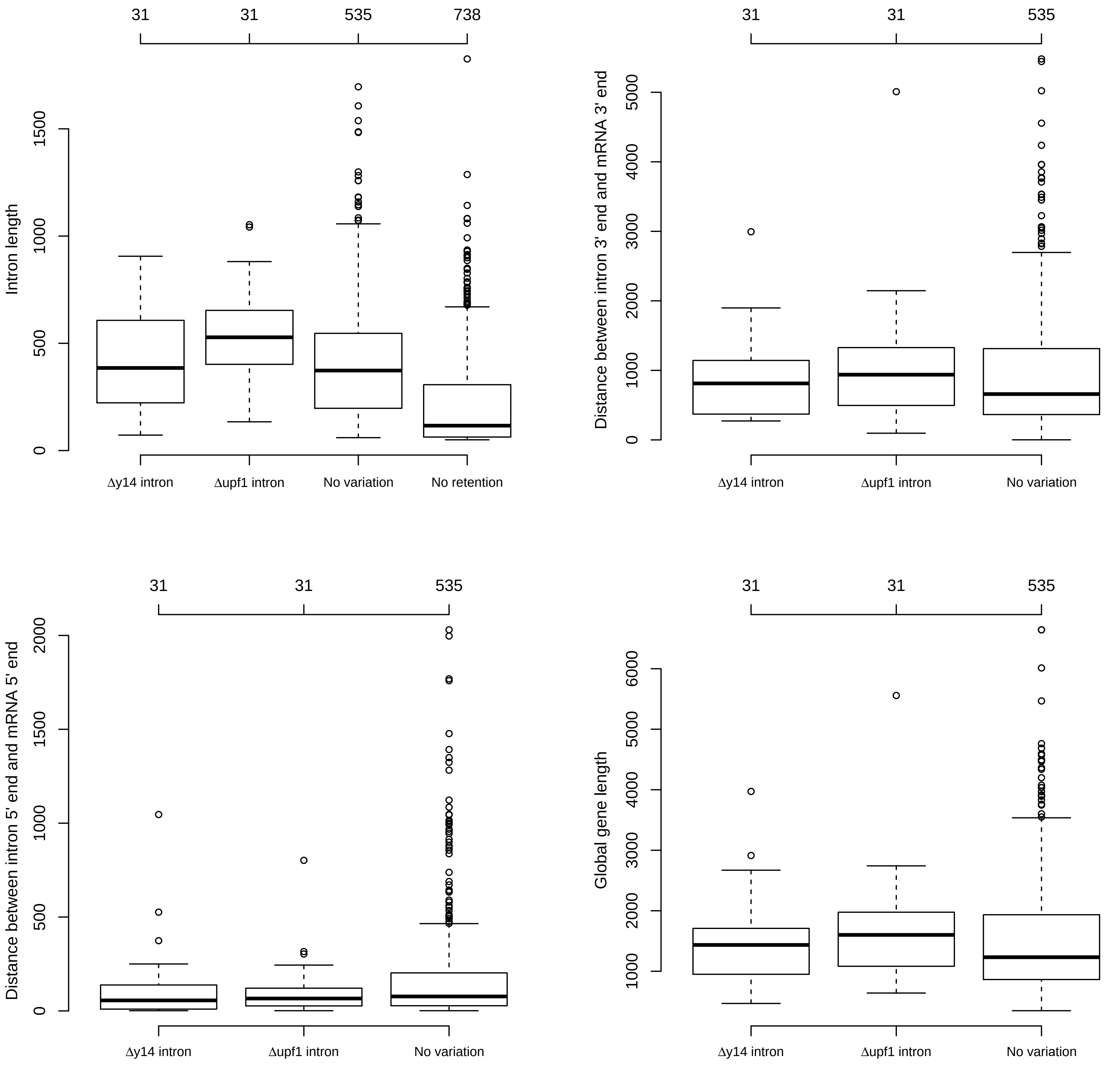

"Δy14 intron" corresponds to DR introns associated with non-differentially expressed genes when comparing Δy14 and the control strain. "Δupf1 intron" is the same for Δupf1 mutant. "No variation" corresponds to the 535 introns that are not DR in at least one of the four pairwise comparisons. "No retention" corresponds to the 738 introns filtered at the beginning of the analysis. Four intron structural parameters were considered: intron length (top left corner), distance between introns 3' end and mRNA 3' end (top right corner), distance between intron 5' end and mRNA 5' end (bottom left corner), and gene length (the bottom right corner).

Supplementary Table S1: Genome sequences and annotation used in this study

Species	Strains	Database	Reference
Blastobotrys adeninivorans	LS3	GRYC	Kunze et al. 2014
Candida albicans	SC 5314	CGD	Jones et al. 2004
Candida glabrata	CBS 138	GRYC	Dujon et al. 2004
Candida parapsilosis	CDC317	Broad Institute	Butler et al. 2009
Candida tenuis	ATCC 10573	NCBI	Wohlbach et al. 2011
Candida tropicalis	MYA-3404	Broad Institute	Butler et al. 2009
Clavispora lusitaniae	ATCC 42720	Broad Institute	Butler et al. 2009
Cryptococcus neoformans var. grubii	H99	NCBI	Janbon et al. 2014
Cyberlindnera fabianii	YJS 4271	GRYC	Freel et al. 2014
Debaryomyces hansenii	CBS 767	GRYC	Dujon et al. 2004
Emericella nidulans	FGSC A4	NCBI	Galagan et al. 2005
Eremothecium gossypii [1]	ATCC 10895	GRYC	Dietrich et al. 2004
Eremothecium cymbalariae	DBVPG 7215	NCBI	Wendland & Walther 2011
Fusarium graminearum	PH-1	NCBI	Cuomo et al. 2007
Geotrichum candidum	CLIB 918	NCBI	Morel et al. 2015
Kazachstania naganishii	CBS 8797	NCBI	Gordon et al. 2011
Kluyveromyces lactis	CLIB 210	GRYC	Dujon et al. 2004
Kluyveromyces marxianus	DMKU3-1042	NCBI	Lertwattanasakul et al. 2015
Kodameae ohmeri [6]	CBS 2037		Morales et al. unpublished
Komagataella phaffii	GS115	Gent University[3]	De Schutter et al. 2009
Kuraishia capsulata	CBS 1993	GRYC	Morales et al. 2013
Lachancea kluyveri	CBS 3082[T]	GRYC	Souciet et al. 2009; Vakirlis et al. 2016
Lachancea thermotolerans	CBS 6340	GRYC	Souciet et al. 2009; Vakirlis et al. 2016
Lachancea waltii [4]	NCYC 2644	GRYC	Kellis et al. 2004; Di Rienzi et al. 2011
Naumovozyma dairenensis	CBS 421	NCBI	Gordon et al. 2011
Naumovozyma castellii	CBS 4309	NCBI	Gordon et al. 2011
Neurospora crassa	OR74A	NCBI	Galagan et al. 2003
Ogataea polymorpha	NCY 495 leu1.1	JGI	Riley et al. 2016
Oleozyma hispaniensis	CBS 9996	GRYC	Michely et al. unpublished
Penicillium oxalicum	114-2	NCBI	Liu et al. unpublished
Rhodosporidium toruloides	CECT1137	NCBI	Morin et al. 2014
Saccharomyces cerevisiae	S288c	SGD	Goffeau et al. 1996
Scherffersomyces stipitis	CBS 6054	JGI	Jeffries et al. 2007
Schizosaccharomyces pombe	972h-	PomBase	Wood et al. 2002

Sugiyamaella lignohabitans	CBS 10342	NCBI	Bellasio et al. 2016
Taphrina deformans	PYCC 5710	NCBI	Cissé et al. 2013
Torulaspora delbrueckii	CBS 1146	NCBI	Gordon et al. 2011
Torulaspora microellipsoides	CLIB 830[T]	GRYC	Galeote et al. 2018
Wickerhamomyces ciferrii	NRRLY-1031	NCBI	Schneider et al. 2012
Yarrowia lipolytica [5]	CLIB 122	GRYC	Dujon et al. 2004
Zygosaccharomyces rouxii	CBS 732	GRYC	Souciet et al. 2009
Zygosaccharomyces bailii	CBS 680	GRYC	Galeote et al. 2013

CGD, Candida Genome Database, http://www.candidagenome.org/
GRYC, Genome Resources for Yeast Chromosomes, http://gryc.inra.fr
JGI, Joint Genome Institute, http://genome.jgi-psf.org/
SGD, Saccharomyces Genome Database, http://www.yeastgenome.org/
[1] Sequence from Dietrich et al. (2004), reannotated by the Génolevures consortium
[3] Gent University, https://bioinformatics.psb.ugent.be/gdb/pichia/
[4] Sequence from the Whitehead Institute/MIT, rearranged by Di Rienzi et al. (2011), reannotated in Neuvéglise's lab
[5] Sequence from Génolevures reannotated in Neuvéglise's lab with RNAseq data, available on GRYC
[6]Morales et al., unpublished; Dikaryome project. The genomes have been sequenced with a combination of Roche 454 and Illumina technologies as described in Morales et al., 2013. The genome of *K. ohmeri* was assembled with Newbler in 10 scaffolds with a cumulative length of 12.32 Mb and a N50 of 1.81 Mb. Gene models were automatically built by GAZE (Howe et al. 2002) using four different resources, i.e. protein sequence alignments, RNA sequencing, expressed sequence tags, and ab initio predictions. The number of predicted protein-coding genes 5,823.

Supplementary Table S3: Percentages of identity and similarity between *Y. lipolytica* Mago, Y14 and Pym proteins and proteins of Yarrowiaceae and Phaffomycetaceae families

**Mago**	**Identity**	**Similarity**
Candida hispaniensis	63% (100/160)	80% (128/160)
Blastobotrys adeninivorans	65% (103/158)	81% (128/158)
Geotrichum candidum	67% (101/151)	84% (128/151)
Wickerhamomyces ciferrii	66% (105/160)	80% (129/160)
Cyberlindnera fabianii	54% (81/151)	74% (112/151)
Sugiyamaella lignohabitans	72% (108/149)	86% (128/149)

**Y14**	**Identity**	**Similarity**
Candida hispaniensis	56% (63/112)	81% (91/112)
Blastobotrys adeninivorans	53% (59/112)	68% (77/112)
Geotrichum candidum	41% (51/123)	60% (74/123)
Wickerhamomyces ciferrii	39% (43/109)	63% (69/109)
Cyberlindnera fabianii	41% (47/115)	65% (75/115)
Sugiyamaella lignohabitans	51% (46/90)	74% (67/90)

**Pym**	**Identity**	**Similarity**
Candida hispaniensis	34% (36/106)	53% (57/106)
Blastobotrys adeninivorans	54% (27/50)	74% (37/50)
Geotrichum candidum	65% (24/37)	83% (31/37)
Wickerhamomyces ciferrii	57% (20/35)	80% (28/35)
Cyberlindnera fabianii	57% (28/49)	71% (35/49)
Sugiyamaella lignohabitans	69% (24/35)	80% (28/35)

Supplementary Table S4: Yeast strains used in this study

Strain	Genotype	Reference
Y. lipolytica strains		
E150/CLIB122	Mat B, *his-1, ura3-302, leu2-270, xpr2-322*	Barth & Gaillardin, 1996
Δku70	E150, *ku70::URA3*	This study
Δku70LEU2	E150, *ku70::URA3, LEU2*	This study
Δupf1	E150, *ku70::URA3, upf1::LEU2*	This study
Δupf2	E150, *ku70::URA3, upf2::LEU2*	This study
Δmago	E150, *ku70::URA3, mago::LEU2*	This study
Δy14	E150, *ku70::URA3, y14::LEU2*	This study
Δy14+Y14	E150, *ku70::URA3, y14::LEU2, Y14, HPH*	This study
E150+Y14-V5	E150, *PromTEF1-Y14V5-TermLIP2, LEU2*	This study
E150+MAGO-FLAG	E150, *PromTEF1-MAGOFLAG-TermLIP2, HPH*	This study
E150+Y14-V5+MAGO-FLAG	E150, *PromTEF1-Y14V5-TermLIP2, LEU2, PromTEF1-MAGOFLAG-TermLIP2, HPH*	This study
E150+UPF3-HA	E150, *PromTEF1-UPF3HA-TermLIP2, URA3*	This study
E150+Y14-V5+UPF3-HA	E150, *PromTEF1-Y14V5-TermLIP2, LEU2, PromTEF1-UPF3HA-TermLIP2, URA3*	This study
E150+MAGO-FLAG+UPF3-HA	E150, *PromTEF1-MAGOFLAG-TermLIP2, HPH, PromTEF1-UPF3HA-TermLIP2, URA3*	This study
E150+Y14-V5+MAGO-FLAG+UPF3-HA	E150, *PromTEF1-Y14V5-TermLIP2, LEU2, PromTEF1-MAGOFLAG-TermLIP2, HPH, PromTEF1-UPF3HA-TermLIP2, URA3*	This study
E150+Y14-V5+PYM-HA	E150, *PromTEF1-Y14V5-TermLIP2, LEU2, PromTEF1-PYMHA-TermLIP2, URA3*	This study
E150+MAGO-FLAG+PYM-HA	E150, *PromTEF1-MAGOFLAG-TermLIP2, HPH, PromTEF1-PYMHA-TermLIP2, URA3*	This study
E150+Y14-V5+MAGO-FLAG+PYM-HA	E150, *PromTEF1-Y14V5-TermLIP2, LEU2, PromTEF1-MAGOFLAG-TermLIP2, HPH, PromTEF1-PYMHA-TermLIP2, URA3*	This study
E150+Y14-V5+PYMΔMBD-HA	E150, *PromTEF1-Y14V5-TermLIP2, LEU2, PromTEF1-PYMΔMBDHA-TermLIP2, URA3*	This study
E150+MAGO-FLAG+PYMΔMBD-HA	E150, *PromTEF1-MAGOFLAG-TermLIP2, HPH, PromTEF1-PYMΔMBDHA-TermLIP2, URA3*	This study
E150+Y14-V5+MAGO-FLAG+PYMΔMBD-HA	E150, *PromTEF1-Y14V5-TermLIP2, LEU2, PromTEF1-MAGOFLAG-TermLIP2, HPH, PromTEF1-PYMΔMBDHA-TermLIP2, URA3*	This study
E150+Y14-V5+MAGO-FLAG+ALYREF-HA	E150, *PromTEF1-Y14V5-TermLIP2, LEU2, PromTEF1-MAGOFLAG-TermLIP2, HPH, PromTEF1-ALREFHA-TermLIP2, URA3*	This study
S. cerevisiae strains		
PJ694α	*MATα*, *trp1*-901, *leu2*-3,112, *ura3*-52, *his3*-200, *gal4Δ*, *gal80Δ*, *LYS2::GAL1-HIS3*, *GAL2-ADE2*, *met2::GAL7-lacZ*	Fields & Song, 1989

Supplementary Table S5: Quantitative data on RNA-Seq experiments

Strain	Replicate	Raw reads	Filtered reads[1]	Mapped reads[2]
Δku70LEU2	A	24,508,837	21,813,769 (89,0%)	21,359,103 (97.9%)
Δku70LEU2	B	27,976,611	24,913,901 (89,1%)	24,403,168 (98.0%)
Δku70LEU2	C	24,618,813	22,750,946 (92,4%)	22,310,023 (98.1%)
Δy14	A	18,270,603	16,349,962 (89,5%)	15,997,881 (97.9%)
Δy14	B	27,944,085	25,742,558 (92,1%)	25,123,828 (97.6%)
Δy14	C	26,553,747	24,576,145 (92,6%)	24,060,150 (97.9%)
Δmago	A	23,923,505	21,346,156 (89,2%)	20,926,916 (98.0%)
Δmago	B	26,590,344	23,911,883 (89,9%)	23,409,392 (97.9%)
Δmago	C	28,066,381	25,259,806 (90,0%)	24,727,374 (97.9%)
Δupf1	A	27,175,401	24,311,540 (89,5%)	23,818,816 (98.0%)
Δupf1	B	26,395,206	23,580,816 (89,3%)	23,059,809 (97.8%)
Δupf1	C	28,137,998	25,256,168 (89,8%)	24,717,790 (97.9%)
Δupf2	A	26,132,423	23,183,845 (88,7%)	22,714,278 (98.0%)
Δupf2	B	25,231,347	22,405,721 (88,8%)	21,954,688 (98.0%)
Δupf2	C	29,493,368	26,393,768 (89,5%)	25,839,641 (97.9%)

[1] Within brackets, the percentage of filtered reads compared to raw reads

[2] Within brackets, the percentage of Mapped reads compared to filtered reads

Supplementary Table S8: List of primers used in this study

Number	Name	Sequence
**Construction of the pAS2ΔΔ and pACT2 recombinant vectors expressing Y14 and Mago proteins**		
1	Y14_171_NcoI	GG**CCATGG**CCACGGAGGGATGGATC
2	Y14_497_BamHI	CC**GGATCC**TATCGTCTTCGTCCAGG
3	Mago_0_NcoI	GG**CCATGG**CGGAAGAAGAGGAAT
4	Mago_585_BglII	CC**AGATCT**AAATTGGCTTAATCTTGAAAT
**Construction of the p*TEF* recombinant vectors expressing tagged proteins**		
5	Y14_ATGV5_BamHI	CC**GGATCC**CACAATGGGTAAACCAATTCC
6	Y14_3V5R	ACCCAACAATGGATTTGGAATTGGTTTACCCATTGTGTCGTGATGCCGTTG
7	Y14_4V5F	CCAAATCCATTGTTGGGTTTGGATTCAACTGGTATTACAGAAAACGAACCC
8	Y14_STOP_AvrII	GG**CCTAGG**CTATCGTCTTCGTCCAGGAC
9	Mago_ATG_BglII	GG**AGATCT**CAACATGGCGGAAGAAGAGG
10	Mago_FLAG_STOP_SpeI	GG**ACTAGT**TATTTATCATCATCATCTTTATAATCCCCTAGGATTGGCTTAATCTTGAAATGCAG
11	Pym_ATG_BamHI	CC**GGATCC**CACAATGGCGTCCCTCACAGGAAC
12	Pym_HA_STOP_SpeI	CC**ACTAGT**TAAGCGTAATCTGGAACATCGTATGGGTATCCTAGGTTTCTTTTGTGTGGAGGAATG
13	PymdeltaMBD_F	CACAGGAACATATGAAAAGGATATCAAAGAAGATGTTCCAAAGTACA
14	PymdeltaMBD_R	TGTACTTTGGAACATCTTCTTTGATATCCTTTTCATATGTTCCTGTG
15	ALYREF_ATG_BamHI	CC**GGATCC**CACAATGGATCAGGCCCTGGACG
16	ALYREF_STOP_AvrII	GG**CCTAGG**TCCTGCATATAACTGTCGAGC
17	Upf3_ATG_BamHI	CC**GGATCC**ACACAATGGAATTCGACCGTTCGC
18	Upf3_STOP_NheI	CC**GCTAGC**TGCGGAGCAGGTGCAGATG
19	PromTef1F	GGTATAAAAGACCACCGTCC
20	TermLip2R	AGATACCACAGACACCCTAG
***Ku70* deletion**		
21	Ku70Ver1	CAGATATCAATGAATTAAGTCTCCGTGTTACCATC
22	Ku70Ver2	GAGGTCCAGGTCAAGAGTCTGTTGAAGATG
***Mago* and *Y14* deletion**		
23	Mago-P1	TTCGGTTGGAACAGCTGTTGGAGAC
24	Mago-P2ura	ATGGCTCTCTGGGCGGAATTCGAAGATGTCGTGCAGTGCAGCAATC
25	Mago-P2leu	ATGATGACTCAGGCGGAATTCGAAGATGTCGTGCAGTGCAGCAATC
26	Mago-T1ura	GTTGTTGTGTTTCTCGGAATTCAAGGTCGAGATTCGACAGGAGGTTC
27	Mago-T1leu	CTTGTTACTGTATATTCGAATTCAAGGTCGAGATTCGACAGGAGGTTC
28	Mago-T2	GACTACTGGCTTCTCAAGCTCTACC
29	Mago-V1	TTCATAGGCAGGTCTGCCACTGTC
30	Mago-V2	GGGATATCAATGAGGAGCGAATCG
31	Y14-P1	AAGCCAGGAGTCAGCAGTCGATCAG
32	Y14-P2ura	ATGGCTCTCTGGGCGGAATTCTCAAGTCGTCCAGTGTGGTTGCATG
33	Y14-P2leu	ATGATGACTCAGGCGGAATTCTCAAGTCGTCCAGTGTGGTTGCATG
34	Y14-T1ura	GTTGTTGTGTTTCTCGGAATTCCAGGAGTCCTGGACGAAGACGATAG
35	Y14-T1leu	CTTGTTACTGTATATTCGAATTCCAGGAGTCCTGGACGAAGACGATAG
36	Y14-T2	GTCGTGCTTCTGTGTATCGAGAATC
37	Y14-V1	AAGTCTGACGCTTTGAAGGATCGG
38	Y14-V2	ACGATAGCGGTCGCCATTTCAATG
39	URA-A	GAATTCCGCCCAGAGAGCCATTGACGTTC
40	URA-B	GAATTCCGAGAAACACAACAACATGCCCC
41	LEU-A	GAATTCCGCCTGAGTCATCATTTATTTACC
42	LEU-B	GAATTCGAATATACAGTAACAAGCTACCAC
***Y14* Complementation**		
43	Y14_PROM_ClaF	CC**ATCGAT**AAGAGTGCTAGGCACGGC
44	Y14_TERM_SacIIR	GG**CCGCGG**GACTCGTCAACGAAGACGCAG
**qPCR**		
45	TFC1-F	AGATGCTGAGATGGACACTT
46	TFC1-R	GTAATGACGCAGTCCCTGG
47	YRA1-4F: Exon1-Exon2	CATTCGAGACTACTTCAAGACTGAGAT
48	YRA1-4R: Exon1-Exon2	CGGTCGATGGAAGTACCGT
49	YRA1-5F: Intron1	CATGCTTATATGGCCAACCGAC
50	YRA1-5R: Intron1	GCTTTCAATCCGGCTCGT
51	SUB2-3F: UTR-Exon1	TCACACTCAACAATGTCCCACG
52	SUB2-3R: UTR-Exon1	GCTTGAGCAAGAAGTCTCGGAAA
53	SUB2-2F: Intron1	TGGGTACAGATAGTTGTTGAGAC
54	SUB2-2R: Intron1	TTGCATCATAATCATGGTAACGAGAG

References for supplementary files

Bellasio M et al. (2016) Complete genome sequence and transcriptome regulation of the pentose utilizing yeast *Sugiyamaella lignohabitans*. *FEMS Yeast Res*. **16**(4) pii: fow037.

Bono F, Ebert J, Unterholzner L, Güttler T, Izaurralde E, Conti E (2004) Molecular insights into the interaction of PYM with the Mago-Y14 core of the exon junction complex. *EMBO Rep*. **5**(3):304–10.

Borneman AR, Forgan AH, Pretorius IS, Chambers PJ (2008) Comparative genome analysis of a *Saccharomyces cerevisiae* wine strain. *FEMS Yeast Res*. **8**:1185–1195.

Buchwald G, Ebert J, Basquin C, Sauliere J, Jayachandran U, Bono F, Le Hir H, Conti E (2010) Insights into the recruitment of the NMD machinery from the crystal structure of a core EJC-UPF3b complex. *Proc Natl Acad Sci U S A* **107**: 10050–5

Buchwald G, Schussler S, Basquin C, Le Hir H, Conti E (2013) Crystal structure of the human eIF4AIII-CWC22 complex shows how a DEAD-box protein is inhibited by a MIF4G domain. *Proc Natl Acad Sci U S A* **110**: E4611–8

Butler G et al. (2009) Evolution of pathogenicity and sexual reproduction in eight *Candida* genomes. *Nature*. **459**:657–662.

Chuang TW, Lee KM, Lou YC, Lu CC, Tarn WY (2016) A Point Mutation in the Exon Junction Complex Factor Y14 Disrupts Its Function in mRNA Cap Binding and Translation Enhancement. J *Biol Chem* **291**(16):8565–74.

Cissé OH et al. (2013) Genome sequencing of the plant pathogen *Taphrina deformans*, the causal agent of peach leaf curl. *mBio*. **4**:e00055–00013.

Dietrich FS et al. 2004. The Ashbya gossypii genome as a tool for mapping the ancient Saccharomyces cerevisiae genome. *Science*. **304**:304–307.

Dietrich FS, Voegeli S, Kuo S, Philippsen P. 2013. Genomes of *Ashbya* fungi isolated from insects reveal four mating-type loci, numerous translocations, lack of transposons, and distinct gene duplications. *G3 Bethesda Md*. **3**:1225–1239.

Dujon B et al. 2004. Genome evolution in yeasts. *Nature*. **430**:35–44.

Gehring NH, Neu-Yilik G, Schell T, Hentze MW, Kulozik AE (2003) Y14 and hUpf3b form an NMD-activating complex. *Mol Cell* **11**(4):939-49.

Fribourg S1, Gatfield D, Izaurralde E, Conti E (2003) A novel mode of RBD-protein recognition in the Y14-Mago complex. *Nat Struct Biol* **10**(6):433-9.

Galeote V, Bigey F, Devillers H, Neuvéglise C, Dequin S (2013) Genome Sequence of the Food Spoilage Yeast *Zygosaccharomyces bailii* CLIB 213T. *Genome Announc*. 1. doi: 10.1128/genomeA.00606-13.

Goffeau A et al. (1996) Life with 6000 genes. *Science*. **274**:546, 563–567.

Gordon JL et al. (2011) Evolutionary erosion of yeast sex chromosomes by mating-type switching accidents. *Proc. Natl. Acad. Sci. U. S. A.* **108**:20024–20029.

Howe KL, Chothia T, Durbin R (2002) GAZE: a generic framework for the integration of gene-prediction data by dynamic programming. *Genome Res*. **12**:1418–1427.

Jeffries TW et al. (2007) Genome sequence of the lignocellulose-bioconverting and xylose-fermenting yeast Pichia stipitis. *Nat. Biotechnol*. **25**:319–326.

Jones T et al. (2004) The diploid genome sequence of *Candida albicans*. *Proc. Natl. Acad. Sci. U. S. A.* **101**:7329–7334.

Kellis M, Birren BW, Lander ES (2004) Proof and evolutionary analysis of ancient genome duplication in the yeast *Saccharomyces cerevisiae*. *Nature*. **428**:617–624.

Morales L et al. (2013) Complete DNA sequence of *Kuraishia capsulata* illustrates novel genomic features among budding yeasts (Saccharomycotina). *Genome Biol Evol.* 5:2524-39.

Di Rienzi SC et al. (2011) Genetic, genomic, and molecular tools for studying the protoploid yeast, *L. waltii*. *Yeast Chichester Engl.* **28**:137–151.

De Schutter K et al. (2009) Genome sequence of the recombinant protein production host *Pichia pastoris*. *Nat. Biotechnol.* **27**:561–566.

Souciet J-L et al. (2009) Comparative genomics of protoploid *Saccharomycetaceae*. *Genome Res*. **19**:1696–1709.

Steckelberg AL, Boehm V, Gromadzka AM, Gehring NH (2012) CWC22 connects pre-mRNA splicing and exon junction complex assembly. *Cell Rep* **2**: 454-61

Wendland J, Walther A (2011) Genome evolution in the *eremothecium* clade of the *Saccharomyces* complex revealed by comparative genomics. *G3 Bethesda Md*. **1**:539–548.

Wohlbach DJ et al. (2011) Comparative genomics of xylose-fermenting fungi for enhanced biofuel production. *Proc. Natl. Acad. Sci. U. S. A.* **108**:13212–13217.

Wood V et al. (2002) The genome sequence of *Schizosaccharomyces pombe*. Nature. **415**:871–880.
